# Soft Robots Powered by Sustainable Energy

**DOI:** 10.1021/acs.chemrev.5c00258

**Published:** 2026-04-24

**Authors:** Stefano Mariani, Ruowen Tu, Kliton Cikalleshi, Luca Cecchini, Fabian Meder, Laura Margheri, Barbara Mazzolai

**Affiliations:** ‡ Bioinspired Soft Robotics Laboratory, 121451 Istituto Italiano di Tecnologia, Via Morego 30, Genova 16163, Italy; ∥ The Biorobotics Institute, Scuola Superiore Sant’Anna, Pontedera 56025, Italy; ¶ Laboratory for Bioinspired, Bionic, Nano, Meta Materials and Mechanics, Department of Civil, Environmental and Mechanical Engineering, University di Trento, Via Mesiano 77, Trento 38123, Italy; ⊗ Surface Phenomena and Integrated Systems Lab, The BioRobotics Institute, Scuola Superiore Sant’Anna, Piazza Martiri della Libertà 33, Pisa 56127, Italy

## Abstract

Soft robots powered by sustainable energy abundantly available on Earth, such as heat, humidity, sunlight, osmotic potential, pH variation, triboelectricity, and wind, represent a promising shift toward eco-friendly and autonomous robotic systems. Efficiency depends on selecting and engineering responsive materials that directly transform environmental stimuli into mechanical actuation and motion, or harvest and store environmental energy to power actuators. Thermo-responsive materials undergo shape changes with temperature variations, while hygroscopic materials leverage moisture adsorption to induce actuation. Photothermal materials convert sunlight into heat and can combine thermal or hygroscopic actuators for controlled deformation. Osmotic processes drive movement through fluidic interactions, whereas pH-sensitive hydrogels respond to chemical gradients, facilitating controlled motion. Triboelectric materials generate electricity via contact-induced charge transfer, enabling self-powered sensing and actuation, while wind-dispersed structures exploit aerodynamic forces for unique movements. This review explores the critical roles of chemical, physical, mechanical, and environmental properties of materials in designing soft robots for sustainable and autonomous operation. Importantly, the review distinguishes between the broad concept of environmental energy and operation that is energetically sustainable. It systematically evaluates reported actuators and soft robotic systems based on whether their required energy sources and operating conditions are naturally occurring and regenerable, or instead depend on restricted environmental ranges, auxiliary inputs, or laboratory-controlled conditions. By examining material behavior, integration into multifunctional composites, and mechanism design for exploiting sustainable energy, this review identifies both established and emerging pathways toward environmentally realistic, autonomous, and long-lived soft robotic systems, with potential applications in environmental monitoring, reforestation, and other robotic domains.

## Graphical abstract

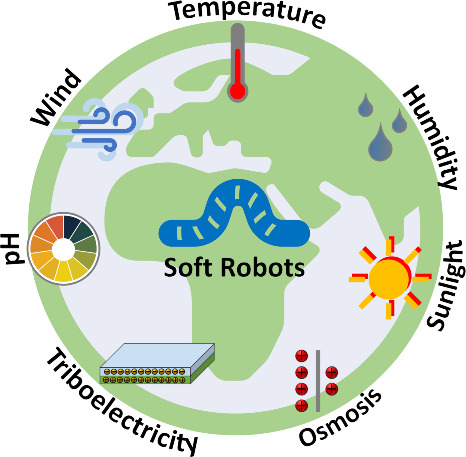

## 1 Introduction

Soft robotics is grounded in advanced technologies that enable robots to move with exceptional dexterity, interact with objects and their surroundings in a compliant and safe manner, and adapt rapidly to dynamic and unpredictable environments.^1–4^ This flexibility allows soft robots to perform complex tasks that require delicate handling and precise movements, making them highly effective in a wide range of applications. These capabilities have enabled soft robotics to expand into diverse fields such as the exploration of natural environments,^5–8^ industrial automation for grasping and manipulation,^9–13^ and the medical field.^14–16^

While advancements in robotics technology offer such a variety of opportunities, progress toward green and renewable energy sources is necessary to ensure a sustainable approach to innovation design.^17–19^ Traditional power sources, such as bulky and heavy batteries, pose significant limitations for soft robotics. These conventional power solutions not only have limited operational lifespans, but they also require frequent recharging or replacement. Additionally, these power sources can contribute to environmental concerns through their materials and disposal methods. Furthermore, their size and weight can hinder the overall performance of soft robots, affecting their agility, adaptivity, and efficiency.

In contrast, directly utilizing renewable and environmental energy offers a promising pathway for powering soft robotic systems. Passive and adaptive actuation strategies, such as those driven by ambient stimuli such as light, heat, or humidity, allow robots to respond intelligently to their surroundings without relying on bulky external power supplies or batteries. This embodiment of physical intelligence leverages material properties and variations of environmental stimuli to achieve autonomous behavior with minimal computational considerations.^20^ Therefore, a growing movement in robotics focuses on developing environmentally responsive and bioinspired robotic systems. These robots, often called “ecorobots”, are designed to interact harmoniously with natural ecosystems and can be sustainably managed at the end of their lifecycle.^18^ By drawing inspiration from nature,^21^ chemists, material scientists, and engineers can create systems that optimize energy efficiency, incorporate green power sources, introduce self-healing capabilities, and utilize biohybrid or naturally derived materials. By leveraging natural energy sources and responsive smart materials, soft robots can significantly represent a more sustainable and eco-friendly solution in the long term.

Complementing passive environmental actuation, advances in flexible and stretchable electronics have enabled soft robots to harvest and store natural energy in a sustainable and autonomous manner.^22,23^ These developments go parallel with the design of environmentally friendly, soft robot-compatible energy harvesting technologies, such as nanogenerators. By integrating these strategies, soft robotic systems can reduce their environmental impact while extending operational lifetimes and enhancing autonomy, particularly in scenarios that demand continuous or untethered performance.^18,19^

In this review, it is important to distinguish between environmental energy and sustainable energy. Environmental energy broadly refers to energy and stimuli originating from the surrounding environment (e.g., light, heat, humidity, chemical gradients, or mechanical motion), irrespective of their magnitude or how they are supplied. In the context of this review, sustainable energy is operationally defined as a subset of environmental energy, referring specifically to energy sources whose required levels and operating conditions are naturally occurring, regenerable, and accessible, such that they can support long-term or autonomous operation in real-world environments without external intervention or consumable inputs. Consequently, while many soft robotic systems interact with environmental stimuli, only those operating within naturally available and self-sustaining stimulus ranges are considered energetically sustainable in this review. Based on this distinction, an energetic sustainability classification framework is adopted throughout the review. Systems capable of operating entirely within naturally occurring and regenerable environmental conditions are classified as fully sustainable, whereas those limited to specific environmental ranges or transient environmental conditions are considered partially sustainable. Systems that rely on environmental energy but require auxiliary external inputs to sustain operation and function are categorized as hybrid-driven, while demonstrations dependent on artificially imposed or non-natural conditions are classified as laboratory-level. This framework provides a consistent basis for evaluating and comparing diverse energy-driven soft robotic systems across different application contexts.

Building on these principles, this review explores the crucial role of the chemical, physical, and mechanical properties of materials in designing soft robots for sustainable and autonomous operation. Specifically, this review examines how the selection and engineering of responsive materials,^24,25^ which convert environmental stimuli into mechanical actuation or other usable energy forms, can serve as a viable approach for sustainable robotics.

The following sections will explore a range of mechanisms that can power soft robots, including thermal, hygroscopic, solar, osmotic, pH-responsive, triboelectric, and wind-dispersal technologies. The primary focus is on direct, passive actuation through physical intelligence in soft robots, but in some sections, advanced energy harvesting, storage and consequent actuation in soft robots are also discussed. Each energy-specific section begins by describing the environmental energy source, highlighting how materials in nature interact with this energy, which leads to the development of bioinspired actuators, systems, and soft robots. Subsequently, the chemical, physical, and mechanical properties and sustainability of naturally derived and artificial materials will be explored, with an emphasis on how these properties can be designed and integrated into soft robots. At the end of each section, a comprehensive review of soft actuators and robots reported in scientific literature will follow, offering a comparative analysis of their performance and applications. The reported actuators and robots are also systematically evaluated using the energetic sustainability framework defined above, explicitly distinguishing systems that operate within fully sustainable environmental conditions from those requiring restricted, hybrid, or laboratory-controlled operation.

## 2 Soft Robots Powered by Temperature

In this section, we examine soft materials and robots driven by ambient temperature variations. We first introduce environmental temperature fluctuations and the mechanisms by which some natural systems can passively actuate in response to thermal changes. We then explore stimulus-responsive materials that adapt to temperature variations, concluding with a comparative analysis of soft robots actuated by thermo-responsive materials.

Earth’s environmental temperature varies widely, but most habitable and practically relevant conditions for biological systems and materials fall between −20 and 60 °C.^26^

Beyond absolute values, dynamic variations are important: daily temporal fluctuations commonly reaching 20–30 °C. In addition to temporal variations, spatial temperature gradients, such as those between air and sunlit or shaded surfaces, can locally exceed 20–40 °C and provide usable driving forces for thermo-responsive systems. These naturally occurring temperature windows and gradients define the operational envelope within which temperature-driven mechanisms must be designed to achieve energetically sustainable actuation.

Biological systems provide instructive examples of how to utilize modest but recurring temperature variations in the environment: *Banksia* seedpods release seeds via temperature-induced softening of specific tissues, which is a natural example of thermally triggered actuation.^27^ This principle is directly transferable to engineered soft actuators, where temperature-induced phase transitions, modulus changes, or volumetric expansion can be exploited to generate motion without external control.

Thermo-responsive soft actuators achieve actuation through several material-level mechanisms, including phase transitions in shape-memory materials, liquid crystalline elastomers (LCEs), and thermo-responsive hydrogels. In contrast to approaches that rely on active heating methods such as Joule heating, magnetic induction, or electromagnetic irradiation, this section focuses on systems that directly harvest low-grade but abundant thermal energy from the environment via passive heat transfer. In addition, other types of sustainable energy sources can combine with thermal actuation through transport and energy conversion, such as thermo-humidity actuation and photothermal actuation, but will be discussed in Section 3 and Section 4, respectively. In this section, the following subsections will review key thermo-responsive materials, present examples of soft actuators and robots powered by environmental temperature changes and thermal gradients, and point out the challenges and gaps toward real-world applications.

### 2.1 Thermo-Responsive Materials

Thermo-responsive soft actuators can undergo mechanical deformation and force generation in response to temperature changes or heat exchange with the external environment. The primary classes of actuating materials used in temperature-driven soft actuators and robots include shape-memory alloys (SMAs), shape-memory polymers (SMPs), thermo-responsive LCEs, and thermo-responsive hydrogels, each exhibiting distinct thermally induced deformation mechanisms, as illustrated in Figure 1a–d.^28^ In addition to material innovations, multimaterial composite designs using conventional materials can also achieve thermally driven actuation. Examples include encapsulated phase change material (PCM) actuators (Figure 1e) and bilayer structures with mismatched coefficients of thermal expansion (CTE). The following subsections introduce the working principles and key material properties of these thermo-responsive materials and provide guidance for selecting suitable materials in the design of temperature-driven, autonomous soft robots.

**Figure 1:**
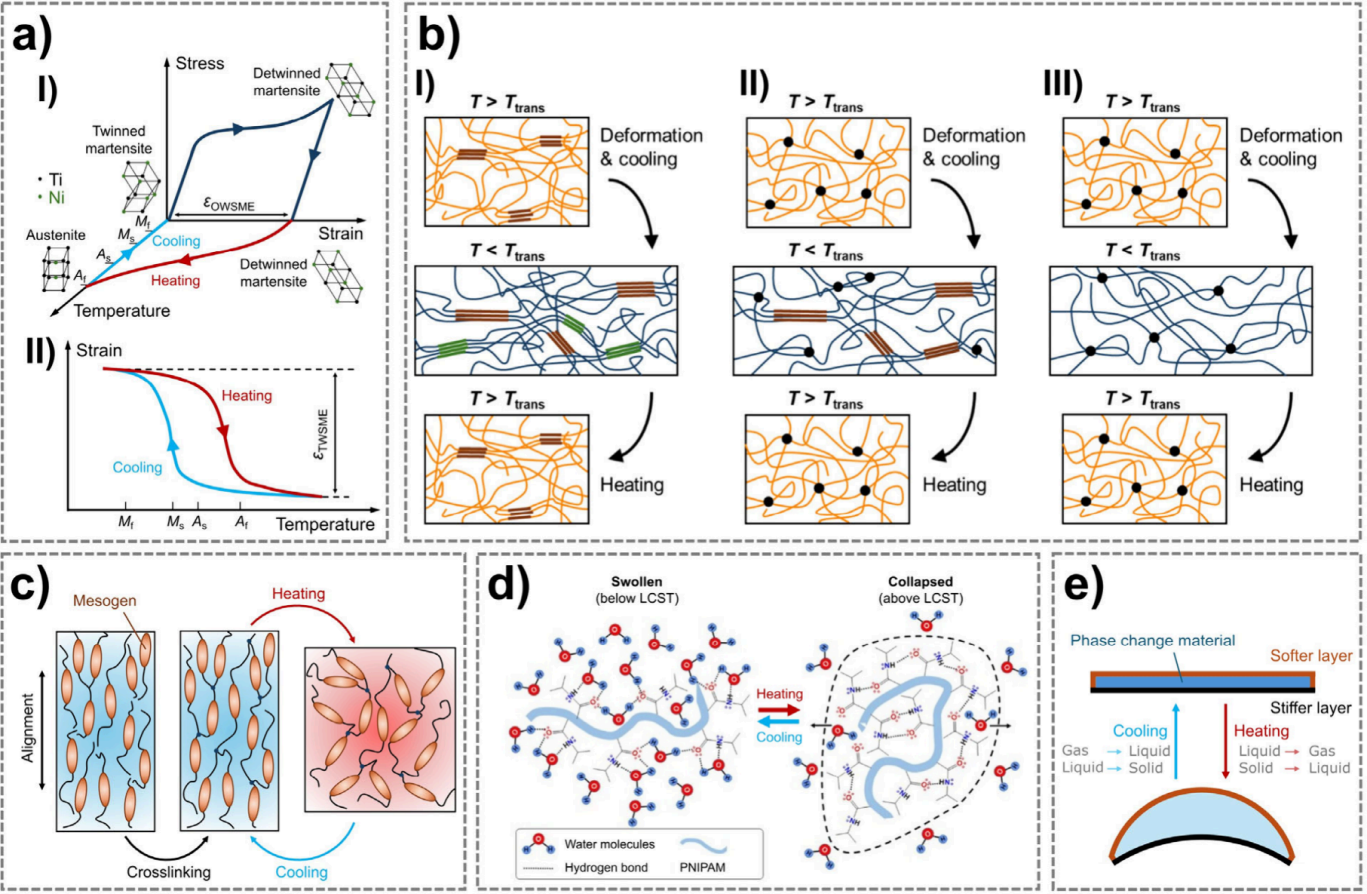
Mechanisms of thermo-responsive materials. a) Working mechanism of a typical NiTi SMA: I) stress–strain–temperature curve in an OWSME cycle, and II) strain-temperature curve in a TWSME cycle. b) Schematics of three molecular mechanisms of SMPs: I) a semicrystalline block copolymer with *T*_trans_ = *T*_m_, II) a cross-linked semicrystalline polymer with *T*_trans_ = *T*_m_, and III) an amorphous polymer network with *T*_trans_ = *T*_g_. c) Reversible actuation of thermo-responsive LCEs after mesogen alignment and cross-linking. d) Schematic representation of the coil-to-globule transition mechanism of a thermo-responsive hydrogel at its LCST. Reprinted with permission from ref^29^ under CC BY NC ND 4.0, Copyright 2023 John Wiley & Sons. e) Working principle of an encapsulated PCM actuator.

#### 2.1.1 Shape-Memory Materials

The first class of functional materials commonly used in thermo-responsive soft robotics is shape-memory materials. These materials can recover their original shape after mechanical deformation when heated above a phase transition temperature, enabling molecular rearrangement. Their behavior is programmable by controlling the heating–cooling cycle and the predefined deformed state, making them widely applicable in deployable structures and soft robotics. The two most common types of shape-memory materials are SMAs and SMPs.

SMAs are metallic alloys that have a reversible martensitic phase transformation. This transformation enables SMAs to transition between a low-temperature, ductile martensitic phase and a high-temperature, rigid austenitic phase (Figure 1a, case I).^30,31^ Nickel-titanium (NiTi) alloys are the most extensively studied SMAs due to their exceptional shape-memory properties, long fatigue life, and biocompatibility.^32^ In addition to NiTi-based SMAs, copper-based (e.g., Cu-Al-Ni, Cu-Zn-Al) and iron-based (e.g., Fe-Mn-Si) alloys provide cost-effective alternatives with tunable transformation temperatures. However, these materials often exhibit drawbacks such as brittleness and reduced fatigue resistance.^33^

SMAs can be classified into two categories based on the reversibility of the shape-memory effect (SME): the one-way shape-memory effect (OWSME) and the two-way shape-memory effect (TWSME). In the OWSME, SMAs can be deformed at low temperatures and retain the deformed shape after the external force is removed. When heated above the phase transition temperature range, they recover their original shape. However, an external force is required to restore the SMA to its deformed state for subsequent cycles. In contrast, SMAs exhibiting TWSME can reversibly switch between two preprogrammed shapes solely through thermal cycling, without the need for mechanical resetting.^34^

To date, the majority of SMAs used in practical applications, such as deployable structures, medical devices, and aerospace components, are based on OWSME due to their high force output, high strain (8%–10%)^31^, and excellent fatigue resistance. Given their high elastic modulus (10–100 GPa), SMAs are often embedded into an elastomer matrix (e.g., silicone rubber), or integrated into an elastomeric structure with preprogrammed shapes (straight, curved, or coiled) to create composite actuators for soft robotic applications.^35–38^ However, the requirement of using mechanical force to reset the original shape of this type of SMAs poses more challenges to their applications in reversible actuation. To solve this problem, antagonistic designs are introduced to one-way SMA-driven soft robots, such as the incorporation of pairs of antagonistic SMAs (using the actuation force of one SMA to reset the other), and coupling with elastic structures to provide the restoration force when the SMAs are turned off.^39,40^ SMAs with TWSME (Figure 1a, case II) are highly desirable for applications requiring periodic motion and simple design. However, achieving this reversible behavior necessitates extensive thermomechanical training, which also reduces their strain output (1%–4%) compared to SMAs with OWSME.^41,42^ To overcome the strain limitation of TWSME wires, they are often coiled into springs, which can amplify the effective length change to values as high as 20%–40%,^43,44^ at the expense of reduced force output. More importantly, the phase transition temperature of SMAs is the key factor to determine if an SMA-based system can be used for environmental temperature-driven actuation without the need for Joule heating or induction heating. Many medical-grade NiTi SMAs have an austenite finish temperature (*A*_f_) below human body temperature (20–36 °C) and a martensite finish temperature *M*_f_ < 0 °C, which can also be actuated in the environmental temperature range. Composition change and thermal aging can fine-tune the transition temperature of NiTi SMAs. For example, Ti-rich NiTi and thermally aged Ni-rich NiTi can increase *A*_f_ to 50–100 °C and be used in a hot outdoor environment,^45^ while Ni_47_Ti_50_Fe_3_ can reduce the transition temperature to *A*_f_ = 11 °C and *M*_f_ = −28 °C for cold environment applications.^46^ Although the transition temperatures of SMAs typically fall within the environmental temperature range (−20–60 °C) on Earth, conventional NiTi SMAs exhibit a thermal hysteresis of about 20–50 °C. This relatively large thermal hysteresis leads to their practical applications mainly relying on Joule heating or induction heating to achieve rapid and significant temperature changes. In contrast, specialized low-hysteresis (*ΔT* = 2–5 °C) SMA compositions, often based on R-phase transformation, have been developed for environmental temperature-triggered actuation, such as thermal switches in space applications.^47^

SMPs are polymeric materials that exhibit a programming-recovery mechanism similar to that of SMAs. While SMPs encompass a wide range of polymers responsive to various external stimuli, this section focuses specifically on thermo-responsive SMPs. The SME in thermo-responsive SMPs is governed by a transition temperature or switching temperature (*T*_trans_), which is typically either the glass transition temperature (*T*_g_) or the melting temperature (*T*_m_), depending on the polymer’s crystallinity. In addition to possessing a distinct *T*_trans_, SMPs require a soft segment in their polymer structure (such as polyolefin, polyether, or polyester) that can deform elastically above *T*_trans_ to achieve high shape recovery efficiency.

A typical programming-recovery cycle of an SMP proceeds as follows:^48^ Initially, after processing (through melt extrusion, casting, spinning, *etc*), the SMP is set into its permanent shape without external forces. To program it into a temporary shape, the SMP is heated above *T*_trans_, deformed under an applied force, and subsequently cooled below *T*_trans_ while maintaining the deformation. Upon removal of the external force, the temporary shape is retained at the lower temperature. When heating above *T*_trans_ again, the SMP recovers its permanent shape, allowing the initiation of a new programming-recovery cycle.

Based on their phase transition mechanisms, SMPs can be classified into three main categories. The first are melting-based SMPs, which utilize crystallization to fix the temporary shape and recover upon heating above *T*_m_ (cases I and II in Figure 1b). The second is glass transition-based SMPs that rely on the transition of the amorphous phase from a rubbery to a glassy state under external load for shape programming, with shape recovery triggered by heating above *T*_g_ (case III in Figure 1b). Finally, reversible bond-based SMPs stabilize their temporary shape through the formation of covalent or noncovalent bonds below a critical temperature, while heating induces bond breakage and restoration of the permanent shape.

The general design principle for SMPs involves the incorporation of three key molecular structure components: a soft, elastic segment that acts as a spring, a switching segment responsible for phase transformation, and a rigid segment that maintains structural integrity above *T*_trans_. Melting-based SMPs are typically composed of cross-linked semicrystalline polymers, block copolymers, or polymer blends, where the low *T*_m_ serves as the switching mechanism.^49^ A common example is chemically- or radiation-cross-linked polyethylene (PE), widely used as a heat-shrink material. In this application, PE is stretched above its *T*_m_ (105–135 °C, depending on the types of PE) and subsequently cooled to induce recrystallization, enabling its heat-shrinkable properties.^50^ Similarly, cross-linked poly­(ε-caprolactone) (PCL) can utilize its melting transition (*T*_m_ ≈ 45–65 °C) as an elastic shape-memory switch, while adding reversible bonds (e.g., a transesterification system) in the cross-linker can also provide plastic (permanent) shape reconfiguration capabilities (Figure 2a).^51^ In block copolymers, low-*T*_m_ polyethers and polyesters are popular candidates for the soft and elastic switching phase in SMPs, since their *T*_g_ is usually far below room temperature. For instance, poly­(ethylene glycol) (PEG) has been integrated into poly­(ethylene terephthalate) (PET),^52^ polyurethane (PU),^53^ and poly­(lactic acid) (PLA)^54^ to form shape-memory block copolymers, utilizing PEG’s melting transition (*T*_m_ ≈ 40–60 °C) as the switching phase. Polyesters such as PCL have also been used in shape-memory block copolymers, combined with rigid segments such as PU,^55–57^ poly­(3-hydroxybutyrate-*co*-3-hydroxyvalerate) (PHBV),^58^ and polyamide (PA).^59^ Polymer blending provides a versatile and tunable approach for developing elastomeric SMPs by combining a thermoplastic elastomer with a low-*T*_m_ switching polymer. Examples include poly­(styrene-*b*-butadiene-*b*-styrene) (SBS)/PCL blend^60^ and polystyrene-*b*-poly­(ethylene-*co*-butylene)-*b*-polystyrene (SEBS)/paraffin blend,^61^ which leverage the elastomeric properties of the thermoplastic phase while incorporating a responsive switching component.

**Figure 2:**
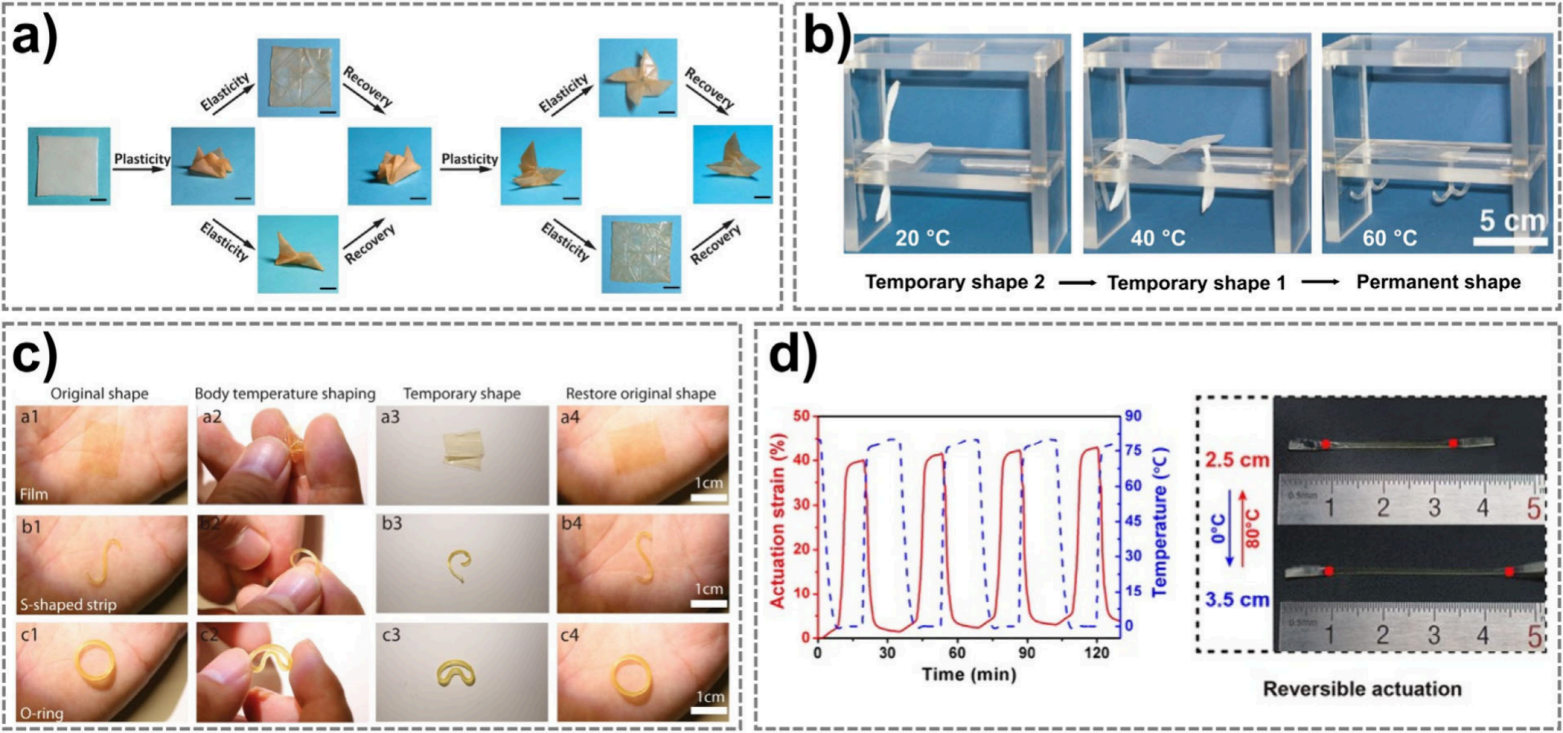
a) Shape manipulation of *T*_m_-based SMP origami structures with thermally distinct elasticity and plasticity. Scale bars, 10 mm. Reproduced from Zhao et al., Science Advances, DOI: 10.1126/sciadv.1501297 [2016], AAAS.^51^ b) A plate with two anchors made of a SMP with two temporary shapes for two-stage shape recovery. Reprinted with permission from ref 68 Copyright 2006 National Academy of Sciences. c) Human palm-triggered shape recovery processes of halogen-bonded SMPs. Reprinted with permission from ref 75 under CC BY 4.0, Copyright 2022 The Author(s). d) Reversible actuation of a SMP with TWSME between 0 and 80 °C, which can generate up to 40% strain. Reproduced from Jin et al., Science Advances, DOI: 10.1126/sciadv.aao3865 [2018], AAAS.^93^

Glass transition-based SMPs can be achieved using polymers with a *T*_g_ above room temperature; however, their shape recovery temperature range is broader compared to melting-based SMPs. In *T*_g_-based SMPs, the soft segment facilitates elastic shape recovery, while an amorphous segment with a *T*_g_ above room temperature governs the switching behavior. For instance, neat ABA triblock-architected styrenic block copolymer (SBC) like SEBS can exhibit significant shape fixity and recovery when programmed slightly below the *T*_g_ of polystyrene (100 °C).^62^ Another category of *T*_g_-based SMPs includes shape-memory copolymer networks, which are covalently cross-linked materials that offer excellent shape stability and creep resistance. Extensive research has been conducted on methacrylate-based SMP networks with *T*_g_-mediated switching, such as poly­(methyl methacrylate)-*graft*-poly­(ethylene glycol) (PMMA-*g*-PEG),^63^ poly­(methyl methacrylate)/star poly­(ethylene glycol) (PMMA/SPEG)^64^ and methyl methacrylate-*co*-poly­(ethylene glycol) dimethacrylate (MMA-*co*-PEGDMA).^65^ In the case of PUs with a soft and amorphous segment, cross-linked ester-type PUs^66^ and amorphous copolyester-urethane networks^67^ have also been developed as *T*_g_-based SMPs. Furthermore, by integrating multiple segments or phases with both *T*_m_- and *T*_g_-mediated switching within a polymer network, SMPs capable of storing and recovering multiple temporary shapes can be designed (Figure 2b).^68,69^

The third category of SMPs utilizes reversible bonds for shape transformation, including both dynamic covalent bonds and noncovalent interactions. Dynamic covalent bonds, which can reversibly break and reform at specific temperatures, are widely used in self-healing polymers and exhibit similar functionality as switching mechanisms in SMPs. Consequently, researchers have developed SMPs incorporating thermo-reversible dynamic covalent bonds, including Diels–Alder reaction,^70^ disulfide bonds,^71^ and imine bonds.^72^ Noncovalent interactions such as hydrogen bonds,^73^ metal–ligand coordination bonds,^74^ and halogen bonds^75^ have also been employed as thermo-responsive switches in SMPs (Figure 2c). Beyond serving as shape-memory switches, these reversible, dynamic bonds have been increasingly explored as network cross-linking points in SMP systems to enhance high-temperature reconfigurability and recyclability.^51,76,77^

The aforementioned SMPs primarily exhibit OWSME and have been widely applied in fields such as heat-shrink tubing, deployable aerospace structures, and biomedical devices.^78–80^ However, for applications in robotics, particularly as actuators or artificial muscles, SMPs with the reversible TWSME, capable of switching between two programmed shapes through heating and cooling, are highly desirable. Thermo-responsive LCEs represent a distinct class of smart materials that inherently exhibit TWSME, which will be discussed in the following section. Beyond LCEs, certain semicrystalline polymer networks also demonstrate TWSME through crystallization-induced elongation and melting-induced contraction. This behavior can be achieved by prestretching the material to induce oriented crystallization. Examples of such systems include cross-linked poly­(cyclooctene),^81^ block PU,^82,83^ ethylene/1-octene copolymers,^84^ poly­(ethylene-*co*-vinyl acetate),^85^ PCL-based networks,^86,87^ and biobased materials.^88^ Another approach for achieving two-way SMPs involves the formation of dual-cross-linking networks (or interpenetrating networks) via photocuring.^89,90^ In this method, a polymer mixture undergoes an initial curing stage followed by mechanical stretching, after which a second-stage photocuring is applied to establish additional cross-links. The resulting dual-cross-linking networks typically consist of an elastomeric phase acting as a spring and a semicrystalline phase serving as the switching component.^91,92^ For instance, a dual-cross-linking network with photoreversible bonds for chain alignment fixation after stretching has been developed, enabling reversible actuation up to 40% strain between 0 and 80 °C (Figure 2d).^93^ In both types of cross-linked networks exhibiting TWSME, the stretching process at elevated temperatures functions as a training step, analogous to the training of two-way SMAs, but typically requiring only a single cycle.

For temperature-driven sustainable soft robotics, shape-memory materials are highly attractive as functional components in deployable, reconfigurable, and bioinspired adaptive systems due to their large actuation strain and the ability to realize multiple programmable states through OWSME. In such one-way programmable applications, both SMAs and SMPs serve as excellent candidates. In addition, many types of SMPs are biodegradable, especially those having ester linkages throughout the main chain, making them highly sustainable options for outdoor disposable applications. By contrast, reversible two-way SMAs and SMPs typically exhibit much more limited actuation strain, which constrains their effectiveness as temperature-driven motors for cyclic soft robotic motion. Specifically, the reversible actuation strain of two-way SMAs is typically only 1%–4% ^41,42^ compared to 8%–10% in one-way SMAs,^31^ while two-way SMPs provide 5%–40%,^86,91,93^ significantly less than the 100%–400% achievable in one-way SMPs.^31,51,68^ To address this limitation, geometric design strategies, such as bending, coiling, and kirigami structures, are often employed to amplify the apparent length change in reversible actuators fabricated from TWSME materials.^43^ Nevertheless, for practical reversible actuation driven by ambient temperature variations or gradients, SMAs offer key advantages over SMPs, including better alignment of *T*_trans_ with environmental conditions, smaller thermal hysteresis (i.e., the difference between heating and cooling *T*_trans_), sharper phase transitions, and faster actuation response. These features render SMAs more suitable for the development of temperature-driven robotic systems.

#### 2.1.2 Thermo-Responsive Liquid Crystalline Elastomers

LCEs are unique materials that merge the reversible ordering characteristics of liquid crystal molecules, known as mesogens, with the flexibility of a cross-linked polymer network. While LCEs can be designed to respond to different stimuli depending on their chemistry, thermo-responsive LCEs based on the nematic–isotropic transition are the most common type. It should be noted that shape-morphing LCEs can also be photothermal-triggered, relying on the same nematic–isotropic transition, but these LCEs will be discussed in the section of sunlight-powered soft robots. Thermo-responsive LCEs are classified as monodomain when the mesogens exhibit uniform alignment throughout the entire sample. In this case, these LCEs are capable of exhibiting TWSME. When they are heated above the nematic–isotropic transition temperature (*T*_NI_), the mesogens lose their orientational order, causing the LCE network to undergo a structural reconfiguration (Figure 1c).^94^ Upon cooling, they can revert to their original form as the elastic properties and self-organizing nature of the LCE restore the network’s initial configuration.^95^ In contrast, when the mesogens organize into smaller, ordered regions with random orientations, the material is referred to as a polydomain LCE. Polydomain LCEs typically do not exhibit substantial shape transformations when activated.^95^

From a chemical perspective, LCEs can be synthesized based on: i) one-step cross-linking; ii) two-step cross-linking; iii) click chemistry.^96^ In one-step cross-linking approaches, polymerization and cross-linking occur in a single reaction step, such as hydrosilylation or free radical polymerization from acrylates. These methods are straightforward and rapid, allowing the production of side-chain LCEs, where mesogens are attached as pendant groups to the polymer backbone. However, they often result in randomly distributed cross-links, which can disrupt the nematic alignment and lead to uncontrollable cross-link densities.^97^ Two-step cross-linking methods involve an initial partial cross-linking to establish an isotropic system, followed by a mesogen alignment process, and a second cross-linking step to stabilize the aligned LCE network. This approach allows for the synthesis of main-chain LCEs, where mesogens are integrated into the polymer backbone. Common reactions used in two-step cross-linking of LCEs include hydrosilylation from polyhydrosiloxane or cyclosiloxane, and step-growth polymerization (polycondensation or polyaddition) from carboxylic acid, hydroxyl, amine, isocyanate, or epoxy.^98,99^ More recently, click chemistry-based cross-linking has gained significant attention due to its ability to create highly uniform networks with precise control. For example, thiol-ene, thiol-acrylate reactions, and azide-alkyne cycloaddition have been employed to construct highly ordered LCE networks with tunable thermo-mechanical properties.^100–102^ The fabrication of LCEs can be achieved under different polymerization conditions. Thermal curing, commonly employed in the two-step cross-linking of bulk LCEs after mesogen alignment, is applicable to peroxide radical cross-linking or thiol–acrylate click reactions. However, thermomechanical relaxation and shape distortion may occur as the aligned material cools. Photocuring with UV or visible light is often used in one-step cross-linking via acrylate-based free radical polymerization or thiol-ene click reactions; this approach is rapid but limited by the shallow penetration depth of light, typically restricting the film thickness to a few hundred micrometers. Solvent-induced curing, in which LCE precursors are dissolved in a solvent, followed by thermal or photoinitiated curing and subsequent solvent evaporation, is primarily applied for solvent casting of LCE films or spinning of LCE fibers, where shear flow facilitates mesogen alignment.

For soft actuator applications, LCEs need a high concentration of mesogenic units and a well-organized polymer network to retain liquid crystal properties. The shape programming and actuation of thermo-responsive LCEs involve the interaction between the elasticity of the polymer network and the orientation of the mesogens. Therefore, mesogen alignment is a critical process for achieving highly responsive LCEs. Mechanical stretching is the most common and established process to align mesogens in the loading direction, leading to reversible actuation of LCEs after cross-linking. For example, stretching of electrospun LCE microfibers can produce monodomain LCE fiber actuators for reversible actuation along the fiber direction (Figure 3a).^103^ Alternatively, other alignment techniques have been developed based on surface alignment techniques,^95,104^ field-assisted alignment techniques^105,106^ and rheology-based three-dimensional (3D) printing processes such as direct ink writing (DIW).^107,108^

**Figure 3:**
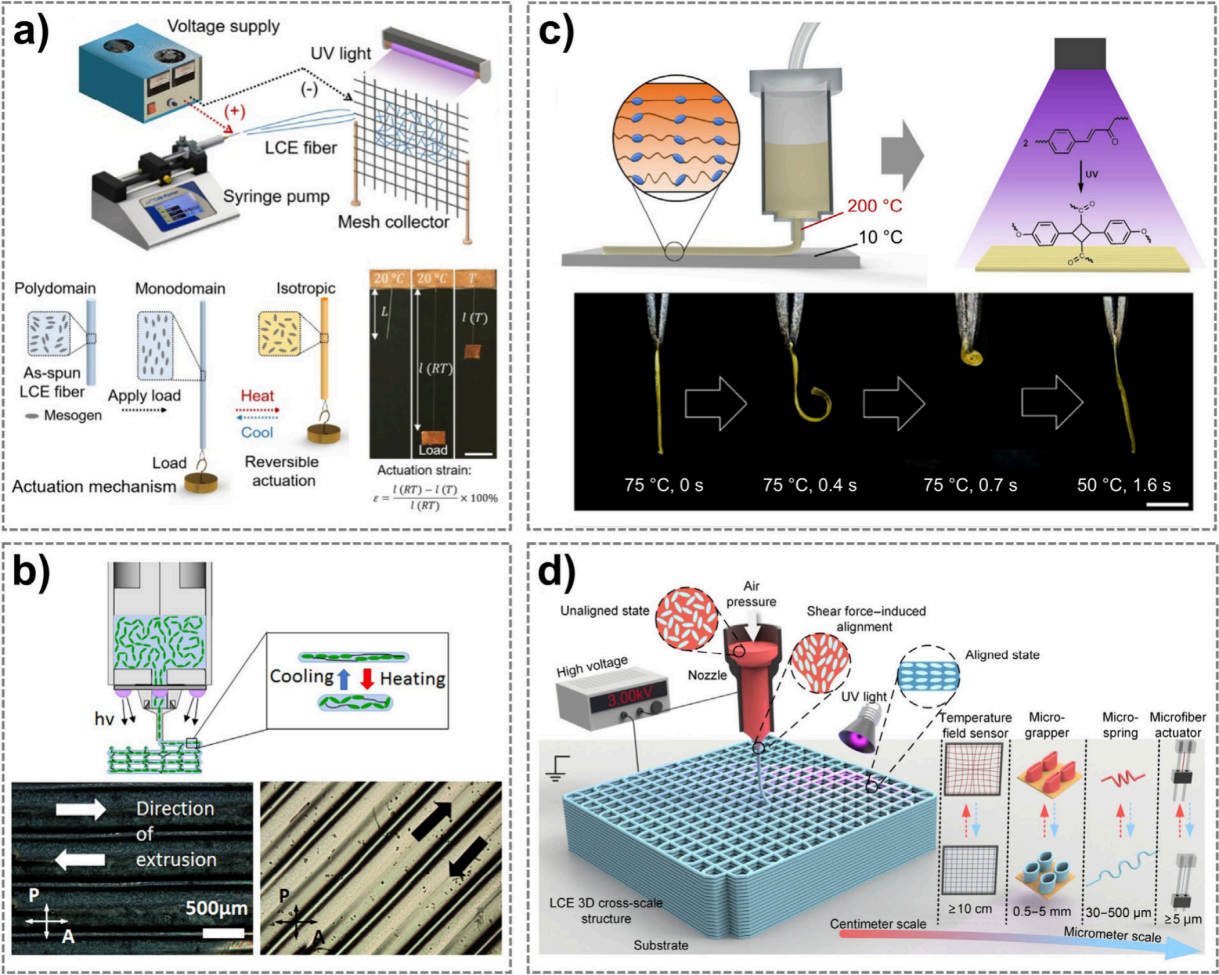
a) LCE microfiber actuators fabricated by electrospinning and stretching. Reproduced from He et al., Science Robotics, DOI: 10.1126/scirobotics.abi9704 [2021], AAAS.^103^ b) 4D printing of LCEs that allows shear-induced mesogen alignment in the direction of extrusion, verified by polarized optical microscopy. Reprinted with permission for ref 110 Copyright 2017 American Chemical Society. c) LCE thermal actuators produced by 4D printing with combined thermal gradient and shear stress-induced mesogen orientation. Reprinted with permission for ref 112 Copyright 2019 American Chemical Society. d) Electric field-assisted extrusion of LCE printing ink that enables the fabrication of multiscale LCE actuators. Reproduced from Feng et al., Science Advances, DOI: 10.1126/sciadv.adk3854 [2024], AAAS.^114^

Recent advancements in thermo-responsive LCEs have focused on material design, including the integration of dynamic covalent bonds for recyclability, novel main-chain LCEs, and nanoparticle-reinforced composite LCEs to enhance actuation force.^109^ Additionally, four-dimensional (4D) printing, as an emerging additive manufacturing process that enables spatial and temporal programming of material properties, has been applied to LCE fabrication for intelligent mesogen alignment (Figure 3b).^110,111^ Zhang et al. utilized a temperature gradient perpendicular to the printing surface during the DIW process to achieve LCE actuators with a controllable orientation gradient (Figure 3c).^112^ A hybrid 4D printing process proposed by Peng et al. combines shear-induced mesogen alignment and in situ laser curing for alignment fixation, enabling one-step fabrication of functionally freestanding LCEs.^113^ Feng et al. developed a melt electrowriting method to fabricate multiscale LCE actuators assisted by an electric field and successfully demonstrated temperature-driven LCE lattice structures, microgrippers, and LCE grid temperature sensors (Figure 3d).^114^

Thermo-responsive LCEs are promising materials for soft actuators, capable of generating significant reversible strains up to 40%–55%. The highly oriented mesogens within LCEs enable directional, anisotropic shape changes, allowing for precise and complex actuation by programming the alignment directions of local mesogens. However, several challenges must be addressed for the practical use of LCEs as functional materials in autonomous and sustainable robots powered by environmental temperature changes or gradients. First, most LCEs have a *T*_NI_ above 50 °C, and require heating above 75 °C for full actuation,^103,107,110,112,114–116^ rendering them unsuitable for phase transitions under sustainable environmental conditions on Earth. Most existing high-performance LCE actuators need to combine with active heating (e.g., Joule heating) to release their full actuation potential.^117^ Thus, tuning the *T*_NI_ of LCEs to lower temperature ranges for more efficient phase transitions, alongside the design of materials for enhanced heat generation, transfer, and collection based on sustainable energy sources (e.g., photothermal effects), is crucial for the development of ambient temperature-driven, LCE-based robots. Second, the inherent softness of LCEs, due to their low elastic modulus (ranging from kPa to MPa), limits their actuation force output, thereby constraining their thrust-to-weight ratio for use in self-propelled robotic applications. Fiber- or nanofiller-reinforced LCEs offer a potential solution to overcome the force output limitations of LCE actuators. Finally, biocompatibility and biodegradability remain major challenges for LCEs, particularly in the context of fully sustainable robotic systems. Most LCEs based on cross-linked mesogenic networks are inherently nondegradable; however, recent studies have demonstrated that incorporating ester-containing star architectures can impart partial biodegradability.^118^ Meanwhile, increasing efforts have focused on enhancing the recyclability of LCEs through reversible chemistries, such as dynamic covalent bonds. Advancing both biodegradability and recyclability will be essential for expanding the role of LCEs in environmentally sustainable and biologically compatible soft robots.

#### 2.1.3 Thermo-Responsive Hydrogels

Hydrogels are water-swollen gels made of hydrophilic polymers that form a 3D network, allowing them to retain large amounts of water.^119^ They can be either chemically cross-linked, with permanent and irreversible bonds, or physically cross-linked, where noncovalent interactions such as host–guest binding, ionic forces, ligand coordination, and hydrogen bonding hold the network together. Hydrogels used for soft actuators and robots can be powered by multiple stimuli (temperature, humidity, pH, light, etc.). In this section, however, we focus specifically on thermo-responsive hydrogels, whose gelation behavior is governed by hydrophobic interactions.

Thermo-responsive hydrogels undergo phase transitions near their critical solution temperature (CST) due to their amphiphilic polymer networks containing both hydrophilic and hydrophobic segments. Depending on the polymer composition, these transitions can occur at either a lower critical solution temperature (LCST), where the hydrogel shifts from a swollen, hydrated state to a collapsed, hydrophobic phase upon heating, or an upper critical solution temperature (UCST), where a sol–gel transition takes place upon cooling. Beyond LCST- and UCST-driven processes, additional mechanisms such as micellization and micelle aggregation may also induce hydrogel shape transformations, although these processes typically result in slower actuation rates and smaller volume changes.^120^

LCST-type hydrogels are among the most used thermo-responsive hydrogels in soft actuators and robotics due to their rapid shrinking response upon heating and fast kinetics. In the hydrated or swollen state, these hydrogels are soluble in water due to strong hydrogen bonding interactions between the polymer and water molecules. When the temperature exceeds the LCST, these hydrogen bonds are disrupted, and intramolecular and intermolecular hydrophobic and hydrogen bonding interactions dominate. This leads to the hydrogel becoming insoluble in the aqueous solution, resulting in dehydration and collapse as the temperature rises.^121^

The earliest documented studies on LCST-type hydrogels, conducted between 1967 and 1968, focused on poly­(*N*-isopropylacrylamide) (PNIPAM).^122,123^ PNIPAM has an LCST around 32 °C, which lies between room temperature and human body temperature, making it highly suitable for temperature-modulated mechanical actuation in soft robotics and biomedical applications.^124^ During the transition, PNIPAM undergoes conformational changes from a coil to a globule state, altering its solubility in water. Above the LCST, PNIPAM adopts a globular form, with most amide groups shielded, causing dehydration (Figure 1d).^29^ Upon cooling below the LCST, hydrogen bonds with water molecules are re-established, leading to the extension of the polymer chains into a coil form and subsequent rehydration. Despite its advantages, PNIPAM has limitations, such as slow actuation rates, weak mechanical properties, and hysteresis after cooling, which often necessitate composition modification for many actuation applications. To enhance the thermo-actuation properties of PNIPAM for various applications, copolymerization and blending with other polymers can be used to tailor its transition temperature and swelling behavior. Copolymerizing PNIPAM with hydrophilic components such as PEG or poly­(acrylic acid) (PAA) can increase the LCST by improving water compatibility, while the addition of hydrophobic comonomers or ionic liquid-based components can reduce the LCST, making it suitable for low-temperature environments. For example, a more hydrophilic poly­(hydroxyl ethylacrylamide-co-*N*-isopropylacrylamide) with an increased LCST of 50 °C has been developed to combine with PNIPAM for composite hydrogels capable of multiple shape transformations.^125^ Additionally, alginate has been incorporated into PNIPAM to form an interpenetrating hydrogel network that has a tunable LCST range between 22.5 and 32 °C (Figure 4a).^124^ In addition to advances in material chemistry, the structural and material design of PNIPAM-based hydrogels represents another important research direction toward the application of thermo-responsive hydrogel actuators. Among these, bilayer bending hydrogels are particularly well studied, offering promising applications in smart grippers and switches.^124,126,127^ For instance, Li et al. developed a bilayer hydrogel composed of a UCST poly­(*N*-acryloyl glycinamide) layer and an LCST PNIPAM-Laponite layer, which exhibited excellent interlayer bonding and rapid bidirectional thermal actuation performance (Figure 4b).^126^

**Figure 4:**
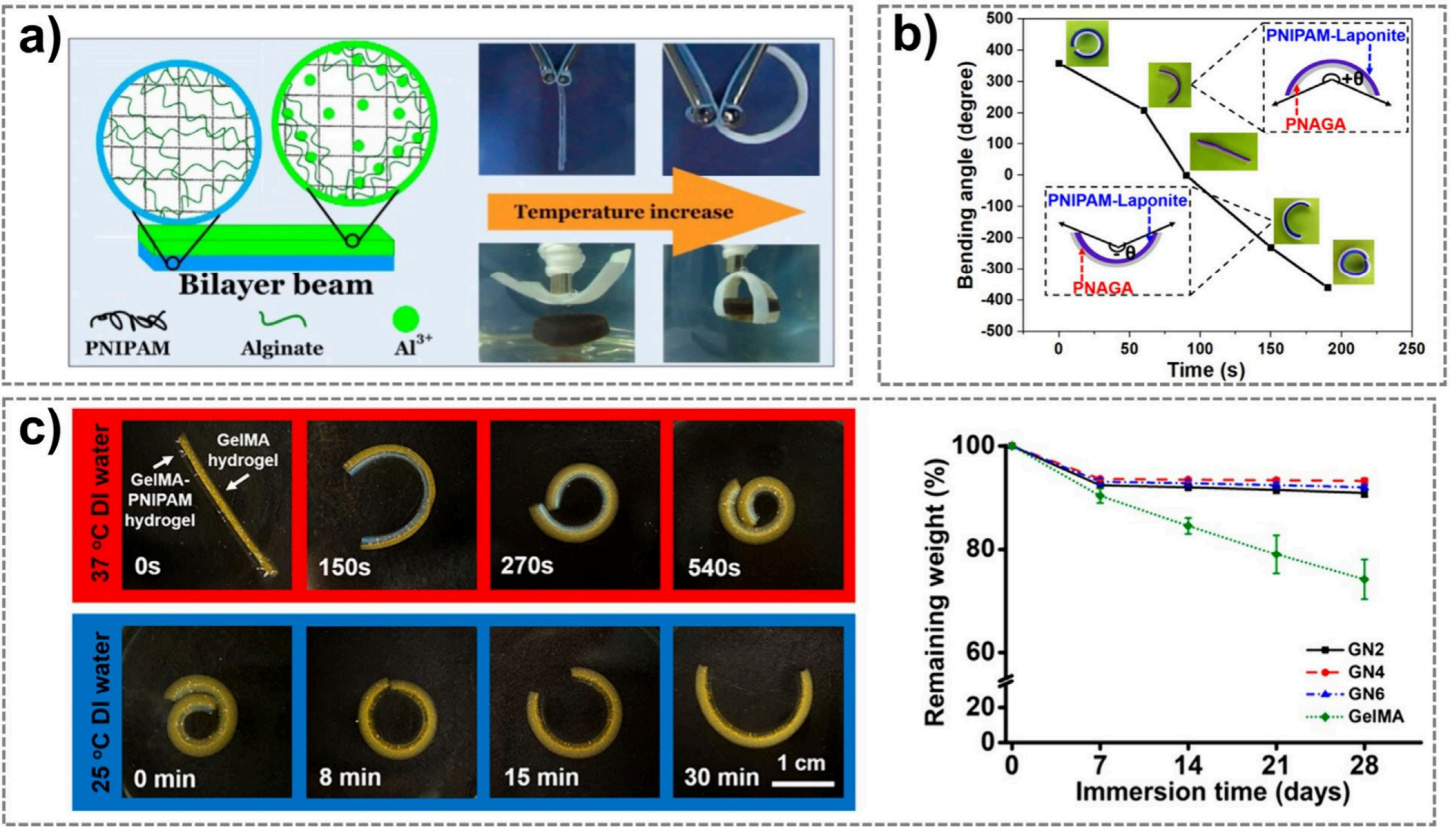
a) An Al-alginate/PNIPAM hydrogel has been synthesized with tunable LCSTs within 22.5–32 °C, which can be used to fabricate bilayer temperature-driven actuators. Reprinted with permission from ref 124 Copyright 2015 American Chemical Society. b) An LCST–UCST bilayer hydrogel can produce rapid and significant bidirectional bending when the temperature is changed from 5 to 45 °C. Reprinted with permission from ref 126 Copyright 2020 American Chemical Society. c) A printable bilayer gelatin methacryloyl (GelMA)/PNIPAM hydrogel can perform reversible bending actuation between 25 and 37 °C. Biodegradation tests show that only the GelMA layer is degradable, while the bilayer hydrogels (GN2–GN6) are more persistent due to the composition of PNIPAM. Reprinted with permission from ref 127 Copyright 2023 American Chemical Society.

PEG-based hydrogels are another type of popular LCST hydrogels, known for their attractive biocompatibility and nontoxicity. Although high molecular weight PEG can exhibit phase separation in water at temperatures around 80–100 °C,^121^ it does not show a sharp LCST like PNIPAM. Moreover, this temperature range is not suitable for actuators driven by typical environmental temperatures. However, copolymerizing PEG with hydrophobic blocks can significantly modulate the LCST, lowering the transition temperature by disrupting polymer–water interactions. For instance, poly­(ethylene glycol)-*b*-poly­(N-acryloyl-2,2-dimethyl-1,3-oxazolidine) hydrogels were synthesized with a tunable LCST ranging from 40 to 72 °C,^128^ and poly­(ethylene glycol)-*co*-poly­(*N*-isopropylacrylamide)-*co*-poly­(ε-caprolactone) hydrogels were developed with a LCST around 31 °C.^129^ By modifying the molecular architecture, hyperbranched copoly­(oligoethylene glycol) can exhibit LCSTs ranging from 25 to 90 °C, which is 5–10 °C lower than the LCST of linear chains with the same monomer compositions.^130^ Additionally, poloxamers, commercially available copolymers of PEG and poly­(propylene oxide) (PPO), have LCSTs varying from 20 to 85 °C, under the trade name of Pluronic.

Natural polymers offer higher biocompatibility and biodegradability compared to the synthetic hydrogels mentioned above, but they often exhibit limited or weak intrinsic thermo-responsiveness. To address this, one approach is grafting synthetic LCST polymers like PNIPAM onto natural polymer backbones (e.g., alginate, chitosan, hyaluronan, cellulose) or creating interpenetrating networks with PNIPAM to impart LCST properties while preserving biodegradability.^127,131,132^ However, the persistence of PNIPAM within these composites still restricts their overall biodegradability, which often results in substantial residual weight of the composite hydrogel even after prolonged degradation (Figure 4c).^127^

More recently, dual CST hydrogels, which possess both LCST and UCST, have been introduced to enable multiple phase transitions and more complex actuation designs. Guo et al. designed a poly­(*N*-acryloyl glycinamide) (PNAGA) network with PNIPAM side-chains, exhibiting dual CST transitions across a wide temperature range (UCST = 14 °C and LCST = 36 °C).^133^ In addition, pH-responsive hydrogels with switchable LCST/UCST behavior or dual CST behavior have been explored by Phunpee et al. and He et al.,^134,135^ offering potential applications in environment-adaptive smart systems.

Thermo-responsive hydrogels show great promise as actuation materials for autonomous, environmentally driven soft robots, primarily due to their mild transition temperatures, which are close to room and body temperature, and their extremely large deformation (>100% strain)^136^ over the transition temperature. Many thermo-responsive hydrogels are also biocompatible and partially biodegradable, making them suitable for medical robots, wearable devices and artificial muscles. Despite these advantages, challenges remain in using thermo-responsive hydrogels for temperature-driven soft robots. These include significantly slower response rates (full actuation cycle up to 15–90 min) compared to SMPs and LCEs due to water diffusion,^126,127,136,137^ ultralow stiffness (modulus typically below MPa level), and limited force output. Furthermore, the presence of water makes hydrogels prone to freezing at subzero temperatures, rendering them unsuitable for direct applications in cold environments. Researchers have developed several strategies to address the freezing problem of conventional hydrogels, either by introducing antifreezing agents into water or by replacing water with alternative liquids in thermo-responsive systems. For instance, mixing water with glycerol, ethylene glycol, or salts can significantly lower the freezing point, enabling hydrogel operation below 0 °C.^138–140^ Alternatively, substituting water with ionic liquids to create ionogels can also broaden the usable temperature range below freezing.^141^ However, in thermo-responsive (hydro)­gels, such modifications of the swelling liquid inevitably alter the phase transition temperature (e.g., LCST or UCST),^142,143^ necessitating additional adjustments in polymer composition to maintain the desired transition within the working temperature range. A further limitation of thermo-responsive hydrogels lies in their reversible actuation performance. Because the phase transition process expels water, which may evaporate or be lost to the environment, the gel can suffer from insufficient water for reswelling. Consequently, reversible actuation of thermo-responsive hydrogels is generally achievable only in high-humidity or underwater conditions, unless encapsulated in a thin, stretchable barrier that prevents water loss.^144,145^

#### 2.1.4 Additional Thermo-Responsive Material Systems

The abovementioned smart materials all exhibit intrinsic thermo-responsive shape morphing and actuation. In addition, the structural and geometric design of composite materials and multimaterial systems can turn conventional materials into thermo-responsive actuators. Among these, two types of thermo-responsive systems are well-studied and engineered. The first category comprises encapsulated PCM actuators (Figure 1e), where the active phase-change component undergoes a substantial density and volume change when heated above its transition temperature (solid–liquid or liquid–gas). To accommodate this expansion, the encapsulating shell is typically designed from compliant and stretchable materials, preventing rupture while transmitting the generated deformation. Upon cooling, the reverse transition drives contraction and recovery of the initial geometry. A wide range of PCMs can be employed to realize thermo-responsive actuators. For applications exploiting environmental temperature variations on Earth (approximately −20–60 °C), common solid–liquid PCMs include low-melting metals, paraffin waxes, and fatty acids, while low-boiling liquids such as acetone, ethanol, and fluorinated fluids are frequently selected for liquid–gas transitions. It should be noted that, in solid–liquid systems, *T*_m_ must be surpassed to activate appreciable volume change, whereas in liquid–gas systems, vapor pressure increases significantly even below the nominal boiling point, enabling actuation through partial evaporation. Although PCMs possess one of the highest energy densities among actuation materials,^146^ most existing studies on encapsulated PCM actuators focus either on soft actuators driven by electrically heated liquid–gas transitions,^147–149^ or on miniaturized, rigid, high-force actuators utilizing solid–liquid transitions.^146^ In contrast, directly harnessing environmental temperature changes to activate soft PCM-based actuators capable of large deformation remains highly challenging and largely unexplored. The primary obstacles lie in fabrication, specifically in achieving reliable encapsulation to prevent leakage, as well as in scaling up the systems for practical use.

The second category consists of bilayer structures with mismatched CTE. As one of the most classic thermo-responsive designs, the bilayer structure is well understood: the actuation curvature or twist angle can be quantitatively related to the CTE mismatch, anisotropic angle, elastic moduli, and thickness ratio of the two layers through established analytical models like classical lamination theory. While the underlying principle is straightforward, effective implementation requires careful consideration of the interfacial bonding between layers, which critically influences durability, fatigue resistance, and reversibility of the actuation. Recent developments have explored diverse material combinations, including metal/ceramic,^150^ polymer/polymer,^151^ polymer/metal,^152^ and polymer/nanomaterial hybrids,^153^ while improving interfacial bonding and enhancing heat transfer efficiency remain as the key research directions.^154^ Additionally, the broad range of available materials for bilayer structures enables the design of fully biodegradable thermal actuators, particularly relevant for soft actuators and robots intended for outdoor applications where sustainability is a priority. In general, most amorphous and semicrystalline biobased or biodegradable polymers (e.g., starch blends, polyhydroxyalkanoates (PHA), polybutylene succinate) exhibit relatively high CTEs, whereas highly crystalline biomaterials such as cellulose display much lower CTE values. One major drawback of this type of bilayer actuator is its relatively small thermally induced strain (<1% within typical environmental temperature ranges) compared to actuators based on phase-transition materials. Consequently, bilayers are often fabricated with thicknesses below a few hundred micrometers to achieve significant curvature or twist angle changes, which inherently restricts their force output and limits their application in load-bearing contexts.

### 2.2 Material Selection Rationale

Table 1 summarizes the representative properties of the thermo-responsive materials discussed above. It is important to note that the actuation strains reported in the table refer to the intrinsic linear strain generated within the material itself, rather than the apparent length change ratio of a structure, which can be significantly amplified through geometric design strategies such as bending, coiling, or serpentine layouts. Selecting materials for environmental temperature-driven soft actuators and robots first requires defining the target application and identifying the relevant working conditions. Broadly, two categories of applications can be distinguished: (i) one-time deployable structures and (ii) reversible devices and soft robots.

**Table 1:** Summary of Representative Properties of Thermo-Responsive Materials

**One-way actuation**					
**Material**	**Actuation temperature**	**Actuation strain**	**Actuation time**	**Biodegradability**	**Cost estimate**
**SMA**	Heating above 10–100 °C^45,46^	8–10%^31^	<2 s by Joule heating^31,156^	No	$1–10/m
**SMP**	Heating above 40–150 °C^51,54,57,68^	Up to 100%–400% ^31,51,68^	<1 min^75^ to 10 min^51,68^	Yes, for some formulations	$20–500/kg

**Two-way reversible actuation**					
**Material**	**Actuation temperature**	**Actuation strain**	**Actuation time (full cycle)**	**Biodegradability**	**Cost estimate**
**SMA**	Heating above 0–60 °C,^41,43,47,157^ thermal hysteresis of 2–50 °C, sharp transition	1%–4% ^41,42^	5–30 s (including < 5 s of heating)^43,156^	No	$1–50/spring
**SMP**	Heating above 43–80 °C,^86,91,93^ thermal hysteresis of 20–50 °C, broad transition	5%–40% ^86,91,93^	5–10 min^86,92,93^	Yes, for some formulations	>$100/kg
**Thermo-responsive LCE**	Heating above 75–160 °C,^103,107,110,112,114–116^ thermal hysteresis of 5–15 °C, broad transition	Up to 40%–55% ^103,107,114^	<1 s for fibers,^103,114^ 5 s to 3 min for films and bulk LCEs^107,115^	Very limited, and only partially degradable	$10–50/g for monomers
**Thermo-responsive hydrogel**	Heating above 22.5–75 °C,^124,126–128,132,158^ thermal hysteresis of 1–5 °C, sharp transition	Up to > 100% ^126,127,136^	<5 min^124,132^ to 15–90 min^126,127,136,137^	Very limited, and only partially degradable	$0.5–5/g for monomers $5–50/g for polymers
**Encapsulated PCM**	Heating above 20–80 °C for solid–liquid transition and 45–130 °C for liquid–gas transition^147–149^	2%–5% for solid–liquid transition and 50%–300% for liquid–gas transition^147–149^	<10 s for solid–liquid transition and 2–6 min for liquid–gas transition^147–149^	Yes, for some formulations	$1–10/kg for PCM, $10–50/piece for actuators
**Bilayer with CTE mismatch**	Within phase transition limit, very low thermal hysteresis	<1%	<1 s for ultrathin strips^153^ and up to 1–3 min for bulk structures^151^	Yes, for some formulations	Varies, from $1/kg polymers to $100/g nanomaterials

For deployable applications, SMAs and SMPs are the most suitable candidates due to their ability to generate large deformation and considerable force output. Many SMA and SMP formulations have transition temperatures that fall within the sustainable environmental temperature range on Earth (approximately −20–60 °C), enabling autonomous deployment when ambient temperature exceeds a threshold. For disposable or single-use deployable structures, SMPs also offer the advantage of biodegradability in certain formulations, such as polyester-based networks, making them attractive for sustainable, low-cost applications.

In contrast, temperature-driven devices and soft robots with reversible motion are at an earlier stage of development but hold great potential. Current promising demonstrations include temperature-triggered switches, grippers, and thermal regulators that operate fully passively by responding directly to ambient temperature changes. Achieving reversible or cyclic motion requires materials with two-way actuation capability. Unlike one-way actuation, which only requires surpassing a threshold temperature, two-way actuation performance depends on the phase transition temperatures, the thermal hysteresis and the sharpness of the phase transition. Given that typical daily temperature swings or spatial temperature gradients in natural environments rarely exceed 30–40 °C (excluding extreme cases), practical thermo-responsive materials must exhibit more strict phase transition temperature ranges with low thermal hysteresis and sharp transitions. Candidate materials include two-way SMAs with R-phase transformation, thermo-responsive hydrogels, and encapsulated PCMs. Two-way SMAs with narrow hysteresis are particularly promising for robotic applications due to their fast response, while their relatively low reversible strain can be amplified through bending, coiling, or embedding SMA wires in elastomeric matrices. Thermo-responsive hydrogels exhibit transition temperatures that align well with ambient ranges, enabling actuation in underwater or high-humidity environments where reversible swelling is possible, or in dry environments if encapsulated within thin, stretchable skins. Encapsulated PCMs are already well-established in MEMS and thermal valve applications, though challenges remain in scaling and fabrication for soft robotic systems. In contrast, thermo-responsive LCEs provide strong reversible actuation and programmable anisotropic strain, but their high transition temperature and cost currently limit their practical application in fully passive, environmentally driven systems; most high-performance LCE devices are coupled with active heating, which has been extensively reviewed^155^ and is beyond the scope of this review. As a future direction, energy harvesting using flexible solar cells and nanogenerators can be integrated into the same system to power LCE-based actuators through electric heating, which is another strategy to realize sustainable energy-powered soft robots. Finally, bilayer actuators based on mismatched CTE represent another viable strategy. Because their response is not governed by a phase transition, they exhibit negligible thermal hysteresis, making them well-suited for environmental temperature-driven actuation. However, their thermal response speed and actuation amplitude decrease significantly with increasing size, making strain amplification through geometric design and enhancement of thermal conductivity crucial for upscaling.

Beyond actuation performance, material sustainability is an increasingly critical criterion for material selection, since the scope of this review already considers sustainable energy. Among the classes of thermo-responsive materials, SMPs, encapsulated PCMs, and CTE mismatch bilayers currently offer the most feasible biodegradable or biobased options. For example, SMPs synthesized from biodegradable segments (e.g., polyester-based copolymers) can degrade under soil or physiological conditions. Biobased PCMs, such as beeswax or butter, can be explored as low-cost, environmentally-friendly options. CTE-mismatch bilayers can also be designed from bioderived or biodegradable components, such as cellulose fibers, natural rubbers, or biodegradable synthetic polymers. In contrast, SMAs and conventional LCEs remain largely nondegradable, while high-performance synthetic thermo-responsive hydrogels (e.g., LCST type) are also nondegradable or partially degradable. In these cases, recent advances have been focused on building networks with reversible bonds to improve the recyclability of LCE and hydrogels. Incorporating biodegradability and recyclability into material selection not only addresses end-of-life concerns but also aligns with the broader vision of sustainable soft robotics operating in natural environments.

### 2.3 Temperature-Driven Soft Actuators and Robots

The development of various thermo-responsive materials has paved the way for temperature-driven soft actuators and robots. Several strategies are available to generate heat or induce temperature changes in thermo-responsive materials, including active methods such as electrical Joule heating and radiative heating (e.g., microwave, infrared laser), as well as passive heat transfer through conduction, convection, or radiation from the surroundings. In the field of soft robotics, active heating approaches are currently the most widely adopted, as they enable rapid, localized heating of thermal actuators and allow for programmable control.^117,159,160^ In contrast, this chapter focuses specifically on passive heat transfer from the natural environment, without relying on external energy conversion mechanisms, with the goal of harvesting environmental heat, a low-grade yet abundant and sustainable energy source, to generate mechanical work.

Within this context, most of the research in the field of thermo-responsive soft actuators and robotics is centered around deployable structures, reconfigurable robots, adaptive devices, and grippers, all of which exploit shape change in response to environmental temperature variations. Among these, deployable structures represent the most established application of one-way thermally activated materials, particularly in space and medical fields. SMA-based triggering devices for solar panels and antennas on small satellites, as well as medical stents and other surgical tools, are already well-developed and commercially implemented, while SMP-based counterparts are progressing toward real-world applications.^80,161,162^ In the specific context of soft robotics, shape reconfiguration of soft structures can be realized by SMPs, hydrogels, and LCEs through either one-way or reversible actuation. For example, Ma et al. and Zhou et al. demonstrated biomimetic robotic flowers that open under elevated temperatures (40–60 °C) and close upon cooling using bilayer hydrogels, which can be integrated with sensors for bioinspired adaptive environmental monitoring.^163,164^ Chen et al. developed a low-temperature-responsive LCE with a crystallization point temperature of 34 °C, enabling the creation of robotic flowers that exhibit temperature-responsive shape changes or function as temperature sensors under ambient environmental conditions.^165^ Nojoomi et al. programmed thermo-responsive hydrogels for spatially and temporally controlled shape morphing through digital light 4D printing, allowing bioinspired dynamic growth of complex 3D structures under temperature change (*T*_trans_ = 32.5 °C) (Figure 5a).^158^ They also demonstrated a stingray-shaped robot capable of producing oscillatory flapping motion of the wings in response to temperature cycles between 31.5 and 33.5 °C. An LCE-based reconfigurable octopus robot designed by Li et al. has shape and color-changing capabilities under different water temperatures, which can further be integrated with magnetic nanoparticles to introduce magnetic field-driven locomotion.^166^ These examples illustrate the significant potential of thermo-responsive materials for developing bioinspired robots that replicate certain functionalities of living organisms within the natural temperature range. In addition to bioinspired designs, a variety of other shape-morphing structures have been developed using thermo-responsive materials, such as SMP-based smart cooling textiles^167^ and hydrogel-based cell culture and delivery devices.^168^ In these applications, one-way actuation of the thermo-responsive materials is typically employed, and the reversibility of the shape transformation is often limited. In addition to shape morphing, other forms of reversible thermal actuators have been studied, such as temperature-adaptive devices,^126,169–171^ multimode fiber actuators,^172^ and artificial muscles.^99^ Among these, intelligent self-adaptive devices that respond to temperature change show the greatest industrial potential, as they enable fully autonomous, wireless, and battery-free operation through the use of thermo-responsive materials. For instance, at the large scale, SMA-controlled sunshades can autonomously open and close in response to ambient temperature variations,^169,170^ while at the small scale, LCE/liquid metal foams have been designed to adaptively modulate thermal conductivity according to the temperature of the target object (Figure 5b).^171^

**Figure 5:**
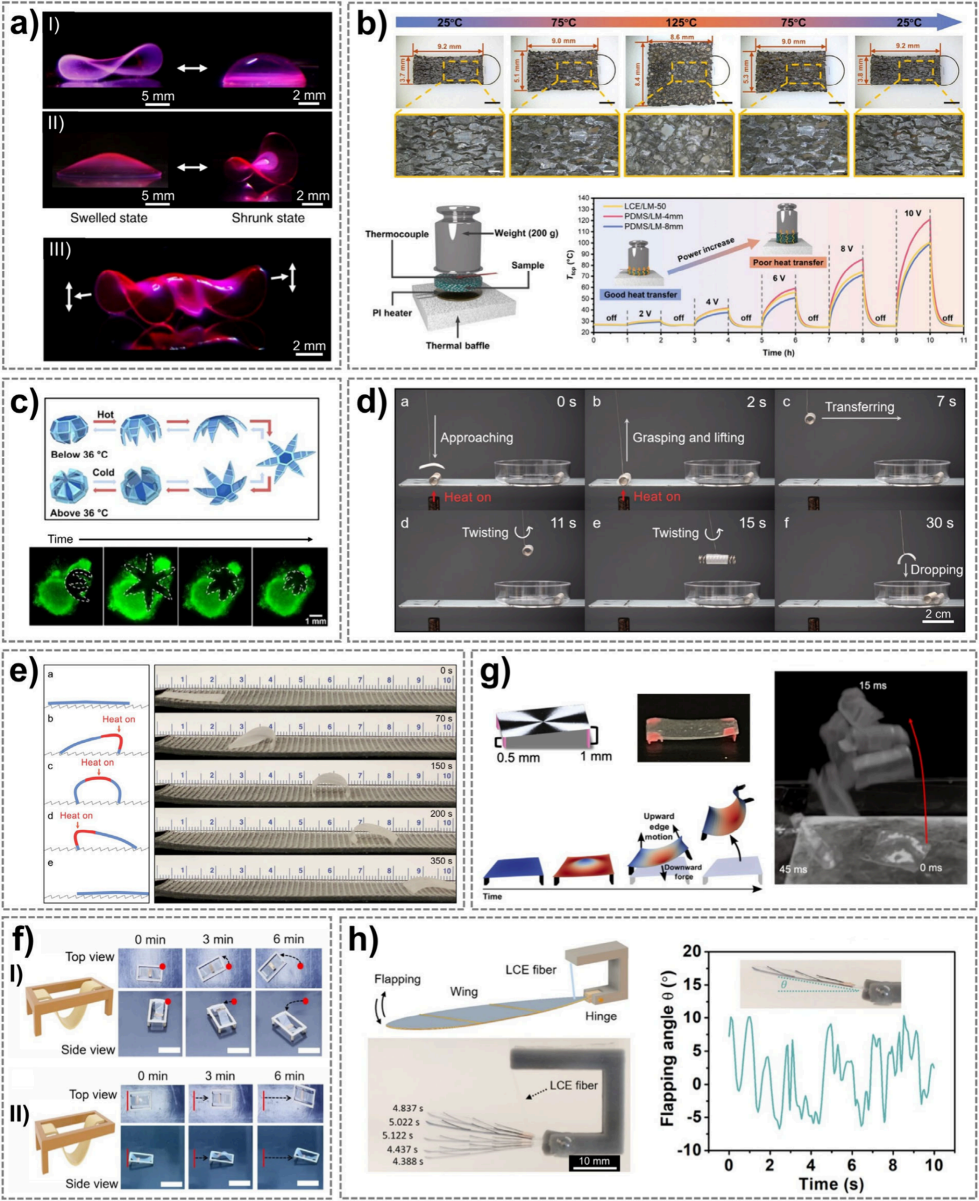
a) Hydrogels with spatially and temporally controlled shape morphing, which can produce a stingray-shaped robot with oscillatory wing flapping under temperature change. Reprinted with permission from ref 158 under CC BY 4.0, Copyright 2018 The Author(s). b) A thermal regulator made of an LCE/liquid metal composite foam can exhibit self-adaptive thermal conductivity based on object temperature variations. Reprinted with permission from ref 171 under CC BY 4.0, Copyright 2023 John Wiley & Sons. c) Thermo-responsive hydrogel-based microgrippers for gripping cells and tissues. Reprinted with permission from ref 173 Copyright 2015 American Chemical Society. d) A soft robotic gripper is used for controlled grasping, delivering, and releasing a hot object. Reproduced from Li et al., Science Advances, DOI: 10.1126/sciadv.abg3677 [2021], AAAS.^115^ e) An LCE crawling robot can move on a ratchet surface by sequentially localized heating on different parts of the robot body. Reproduced from Li et al., Science Advances, DOI: 10.1126/sciadv.abg3677 [2021], AAAS.^115^ f) Crawling robots based on self-regulated snap-buckling of LCE actuators. I) With front legs. Scale bars, 1 cm. II) Without front legs. Scale bars, 2 cm. Reprinted from Chemical Engineering Journal, Vol 500, Choi and Kim, Self-Repeatable snapping liquid–crystal-elastomer actuator, Page 156744, Copyright 2024, with permission from Elsevier.^116^ g) An LCE jumping robot based on concentrically programmed LCE films and a rapid snap-buckling mechanism. Reproduced from Hebner et al., Science Advances, DOI: 10.1126/sciadv.ade1320 [2023], AAAS.^174^ h) An autonomous flapping wing actuated by LCE microfibers that oscillates in a steady temperature field with the gradient. Reproduced from He et al., Science Robotics, DOI: 10.1126/scirobotics.abi9704 [2021], AAAS.^103^

Soft grippers and actuators represent another key application of thermo-responsive materials, particularly for autonomous object manipulation, such as gripping and releasing, based on the temperature of the environment or the object. Biocompatible and stimuli-responsive hydrogels have shown promise in micromedical robotics. Over the past decade, thermo-responsive hydrogels have been explored as microgrippers for manipulating cells and tissues (Figure 5c),^173,175^ as well as for macro-grippers that actuate in warm water,^176^ both operating within temperature ranges close to human body temperature. Beyond medical applications, researchers have incorporated spatially programmed LCEs into a single-part gripper that provides reversible gripping above *T*_NI_ (62 °C) and releasing at room temperature.^177^ Additionally, LCE grippers have been 3D printed using digital light processing (DLP) for controlled hot object grasping, delivery, and release (Figure 5d).^115^ Moreover, Loaiza et al. designed an octopus-inspired morphing surface made of elastomer-NiTi SMA composites that can be programmed into a temperature-triggered gripper,^38^ and Lu et al. developed a hydrogel composite gripper with embedded anisotropic delignified wood that can automatically capture a 50 °C object and release it in a 15 °C water bath.^178^ Although macroscale thermo-responsive actuators are typically characterized by slower actuation rates (with cycle times ranging from a fraction of a minute to more than 1 h), recent advancements have successfully shifted the shape transformation temperature of these materials into the environmental or body temperature range, making fully ambient-powered sustainable actuators a viable possibility.

Soft robotic locomotion powered by environmental temperature change or gradients as a fully sustainable energy source has garnered significant research interest in recent years, becoming a prominent focus within the soft robotics community. However, many demonstrations of temperature-powered soft robots reported to date and referenced below rely on laboratory-controlled heating or cooling that exceeds environmental temperature ranges. While such studies are not directly applicable to natural conditions, they remain valuable as stepping stones toward sustainable robotics. First, rolling represents one of the simplest and earliest forms of locomotion achieved by temperature-driven soft robots. In 2018, Ahn et al. demonstrated the autonomous rolling of an LCE rod (70.0 mm in length and 2.6 mm in diameter) on a hot surface (100 °C), achieving a rolling speed of 6 mm/s on a flat surface and 3 mm/s on an 11° tilted surface.^179^ The mechanism behind the rolling motion is attributed to the cyclic thermal gradient generated in the LCE rod, induced by heating at the contact surface with the hot substrate and cooling in the surrounding air. An initial curvature of the rod is essential to break the symmetry and initiate the rolling motion. Later in 2021, Zhai et al. adopted a similar concept but incorporated 4D printing into the fabrication process of an LCE tubular robot. This robot was capable of rolling on a 160 °C hot surface at a speed of up to 8 mm/s (with a length of 100 mm and a width of 5 mm) and could facilitate cargo transportation, carrying up to 3 g of cargo.^180^ Ding et al. developed a room-temperature shape-programmable LCE and demonstrated a twisted LCE actuator that can roll on an 80 °C hot plate at a speed of 5.7 mm/s.^181^ Thermal gradient-driven rolling on a hot surface has also been extended to more complex self-propelling structures. Kotikian et al. developed a foldable pentagonal prism-shaped rollbot (edge length of 15 mm) equipped with five LCE hinges. This robot could propel itself on a 200 °C hot surface at a speed of approximately 1.3 mm/s.^182^ In all rolling robot cases, the substrate temperature far exceeds the transition temperature of the actuation material due to inefficient heat transfer between the two. Therefore, incorporating high thermal conductivity materials and improving surface contact to enhance heat transfer efficiency is a crucial research direction for developing practical rolling robots for real-world applications.

Crawling or walking is another important locomotion mode for autonomous soft robots. In 2021, Li et al. employed DLP to fabricate an LCE-based crawling robot (18 mm × 13 mm × 0.2 mm) capable of moving at a speed of 20 mm/min on a ratchet surface through sequential localized heating applied to different regions of the robot body (Figure 5e).^115^ Although a heat gun was used as the heating source in this demonstration, the concept is potentially adaptable to outdoor environments where directional heating, such as solar irradiation, occurs naturally. More recently, in 2024, Seung Choi and Kim developed crawling robots utilizing self-regulated snap-buckling of LCE actuators. The cyclic gait was generated through a snap-through motion driven by intermittent contact with a 170 °C substrate, followed by cooling when lifted from the surface (Figure 5f).^116^ Additionally, they demonstrated that a configuration consisting of only two rear legs and a snapping LCE actuator functioning as a third leg enabled directional crawling motion at a speed of approximately 0.5 cm/min.

Jumping is a more complex locomotion mode for soft robots, enabling obstacle avoidance and dynamic movement. In 2018, Gao et al. demonstrated hydrogel-based underwater locomotion using a bilayer hydrogel composite with two distinct LCSTs. This system achieved directional sliding on a glass substrate and controlled jumping over a 10.6 mm lateral distance (35% of body length) by increasing the water temperature from 22 to 47 °C in a single cycle (∼700 s) on a ratchet surface via underwater snap-buckling.^137^ While this study established snap-buckling as a viable mechanism for energy release in temperature-driven soft robots, it also highlighted the challenge of extremely slow actuation in hydrogel systems. Later in 2023, Hebner et al. explored the rapid (millisecond-scale) and powerful snap-through behavior of LCEs, developing a jumping robot based on concentrically programmed LCE films with a modulus gradient through the thickness (Figure 5g).^174^ Their design enabled vertical jumps up to 20 mm on a 160 °C hot surface, and with four supporting legs, the robot achieved directional jumping with a lateral displacement of ∼ 10 mm. Beyond surface-based locomotion, researchers have also investigated LCE actuators operating in continuous thermal gradients. He et al. designed an autonomous flapping-wing device using electrospun LCE microfibers, capable of oscillatory motion (∼15° peak-to-peak flapping angle change) under a steady temperature gradient (*ΔT* ≈ 10–20 °C, Figure 5h).^103^ This work opened new possibilities for self-propelling, environment-driven soft robots that harness natural thermal gradients in air or water, potentially enabling future applications in autonomous flying or swimming locomotion.

In summary, Table 2 provides a literature comparison of several typical temperature-driven soft actuators and soft robots that have been discussed in this section. For the energetic sustainability classification, temperature-driven systems capable of actuation and continuous operation entirely within naturally occurring environmental temperature ranges (−20 to 60 °C) are classified as fully sustainable. Systems whose thermal activation thresholds only partially fall within this range, such that actuation is possible under specific but limited environmental conditions, are considered partially sustainable. In contrast, thermal systems that require temperatures outside natural environmental limits or depend on externally controlled local heating to trigger actuation are categorized as laboratory-level demonstrations.

**Table 2:** Literature Comparison of Several Temperature-Driven Soft Actuators and Robots

**Soft actuator or robot**	**Materials and fabrication process**	**Performance**	**Energetic sustainability classification**	**Applications**	**Ref**
**Stingray-shaped robot**	Hydrogel with spatially and temporally controlled contraction.	The hydrogel grows into a stingray shape, and it achieves wing flapping between 31.5–33.5 °C.	Fully sustainable	Biomimetic soft robot	^158^
	Fabrication: DLP 4D printing.				
**Deployable orbital stent**	Shape-memory PU/gold nanoparticles/nanohydroxyapatite composite.	Stent deployment in the orbit of a rabbit with 44 °C saline solution stimulation.	Laboratory-level	Medical device	^161^
	Fabrication: 4D printing.				
**Intelligent thermal regulator**	LCE/liquid metal foam.	The foam has 121% strain when heated from −20 to 120 °C, and can change thermal conductivity based on the target temperature.	Partially sustainable	Adaptive thermal regulator	^171^
	Fabrication: leaching and infiltration.				
**Soft microgripper**	PNIPAM-acrylic acid hydrogel film.	Autonomous gripping of 37 °C warm tissues.	Fully sustainable	Medical device	^173^
	Fabrication: photopatterning.				
**Hot object gripper**	Bilayer: tempo-oxidized cellulose nanofibers/PNIPAM hydrogel on delignified wood.	Autonomously gripping a 50 °C object and releasing it in 10 °C water.	Partially sustainable	Gripper	^178^
	Fabrication: molding.				
**Artificial muscle**	LCE strip.	A 56 mg artificial muscle lifts 40 g mass (temperature change: 23–90 °C), with a maximum specific work of 63 J/kg.	Laboratory-level	Linear actuator	^115^
	Fabrication: DLP printing				
**Rod-shaped rolling robot**	LCE rod.	With rolling speeds of 6 mm/s on a flat 100 °C surface and 3 mm/s on an 11° tilted surface.	Laboratory-level	Locomotion: rolling	^179^
	Fabrication: molding.				
**Tubular rolling robot**	LCE tube.	With a rolling speed of up to 8 mm/s and a cargo carrying capacity up to 3 g on a 160 °C surface.	Laboratory-level	Locomotion: rolling, cargo transportation	^180^
	Fabrication: 4D printing.				
**Twisted rolling robot**	Shape-programmed LCE strip.	With a rolling speed of 5.7 mm/s on an 80 °C hot plate.	Laboratory-level	Locomotion: rolling	^181^
	Fabrication: molding.				
**Pentagonal prism-shaped rollbot**	Active LCE hinges and passive resin-based structures.	With a rolling speed of 1.3 mm/s on a 200 °C hot plate.	Laboratory-level	Locomotion: rolling	^182^
	Fabrication: DIW.				
**Crawling robot**	LCE film.	With a moving speed of 20 mm/min on a ratchet surface through localized heating (90–140 °C) on different regions of the robot body.	Laboratory-level	Locomotion: crawling	^115^
	Fabrication: DLP printing.				
**Snap-buckling crawling robot**	Bistable LCE film with a 90°-twisted-nematic alignment.	Directional crawling motion based on reversible snap-buckling at a speed of 0.5 cm/min on a 170 °C surface.	Laboratory-level	Locomotion: crawling	^116^
	Fabrication: injection molding and assembly with prebuckling.				
**Snap-buckling hydrogel jumper**	Bilayer composite hydrogel: *N*-isopropylamide and *N*-[3-(dimethylamino)propyl]methacrylamide copolymers with different LCSTs.	Controlled jumping over a 10.6 mm lateral distance in a single cycle (∼700 s, temperature from 22–47 °C) on a ratchet surface underwater.	Partially sustainable	Underwater locomotion	^137^
	Fabrication: sequential free radical copolymerization.				
**Snap-buckling leaping robot**	Concentrically programmed LCE films with a modulus gradient through the thickness.	Directional jumping with a lateral displacement of ∼10 mm or vertical jumping up to 20 mm on a 160 °C surface.	Laboratory-level	Locomotion: Jumping	^174^
	Fabrication: lamination.				
**Flapping wing device**	LCE microfibers on a wing.	Oscillatory flapping motion (∼15° peak-to-peak flapping angle change) under a steady temperature gradient (*ΔT* ≈ 10–20 °C).	Fully sustainable	Flapping flight	^103^
	Fabrication: electrospinning.				

Temperature-driven soft actuators and robots for locomotion can generally be powered by two primary mechanisms: temporal temperature change and spatial temperature gradients. When considering applications in nature and environment-driven systems, both mechanisms are closely linked to the thermal energy naturally available on Earth. Temporal temperature variations occur due to cyclic heating and cooling, such as the temperature fluctuations between day and night (typical *ΔT* < 20–30 °C) or the intermittent exposure to sunlight and shade. Soft robots leveraging this phenomenon can be designed for slow but predictable actuation cycles, making them suitable for long-term deployment in remote areas for tasks such as environmental monitoring, agricultural automation, or passive energy harvesting. On the other hand, spatial temperature gradients, such as the difference in thermal properties between the ground and air, can serve as a sustainable energy source for self-propelled motion. In desert or arid regions, where ground surfaces absorb and retain heat more effectively than the surrounding air (typical *ΔT* < 20–40 °C), thermo-responsive robots can utilize this temperature differential for locomotion. Such robots can autonomously explore terrains, assist in geological surveys, or transport lightweight payloads without requiring batteries or active energy input.

Despite the progress described above, a substantial gap remains between current demonstrations and the envisioned capabilities of temperature-driven soft robots. As summarized in Table 2, temperature-driven locomotion demonstrations are still mostly at laboratory-level, which require conditions impractical for outdoor deployment. Consequently, the technology readiness level (TRL) of ambient temperature-driven soft robots for locomotion remains below 4, falling behind temperature-adaptive smart devices (TRL 4–5). The main challenges are two aspects: (i) the narrow window of temperature change available for reversible thermal actuation, and (ii) the difficulty of harvesting natural spatial temperature gradients and converting them into cyclic thermal inputs for locomotion. Future material development should focus on thermo-responsive systems with sharp phase transitions in the ambient range and minimal thermal hysteresis. At the same time, integrating thermal management strategies into material and structural design will be crucial. For passively heated systems, potential approaches include enhancing thermal conductivity,^183^ optimizing contact area to improve heat collection, incorporating thermal energy harvesting and storage elements,^147,184^ and exploiting different cooling media to accelerate recovery.^185^ Finally, new mechanisms that can cyclically harness naturally available temperature gradients (for example, through self-regulated center-of-mass shifts or buckling instabilities activated by thermal actuation) represent promising directions for advancing environmental temperature-powered soft robotics.

## 3 Soft Robots Powered by Humidity

In this section, we examine soft materials, actuators, and robots driven by ambient humidity variations. Humidity is an attractive environmental energy source for soft robots since relative humidity (RH) fluctuation and spatial variability are abundant naturally and can be converted into mechanical deformation through hygroscopic effects. We briefly introduce humidity as a sustainable environmental stimulus, identify hygromorphic actuation mechanisms observed in natural systems, and focus on how these principles can inform material selection and structural design in humidity-driven soft actuators and robots.

RH is defined as the ratio, often expressed as a percentage, of the partial pressure of water in the atmosphere at a given temperature to the saturation vapor pressure of pure water at that temperature.^186^ It varies widely due to climate, geography, seasons, and typically spanning ∼ 20%–80% RH in outdoor conditions. These variations provide a naturally regenerable stimulus capable of driving repeated deformation in hygroscopic materials. In nature, humidity-responsive motion is commonly realized through hygromorphism, where anisotropic swelling of hydrophilic tissues converts moisture adsorption and desorption into mechanical work.^187–191^ Classic examples include seed awns in the Geraniaceae family (Figure 6a) and oat (Figure 6b), where oriented cellulose fibers and layered tissue architectures produce reversible bending, twisting, or snapping motions in response to humidity changes, enabling locomotion, soil penetration, and dispersal.^187,188,192^ Similarly, pinecone scales exploit bilayered microstructures with differential hygroscopic expansion to achieve slow, reversible opening and closing (Figure 6c).^189–191,193^ These natural systems illustrate key design principles for bioinspired humidity-driven actuators or robots, such as material anisotropy, structural asymmetry, and passive environment response.

**Figure 6:**
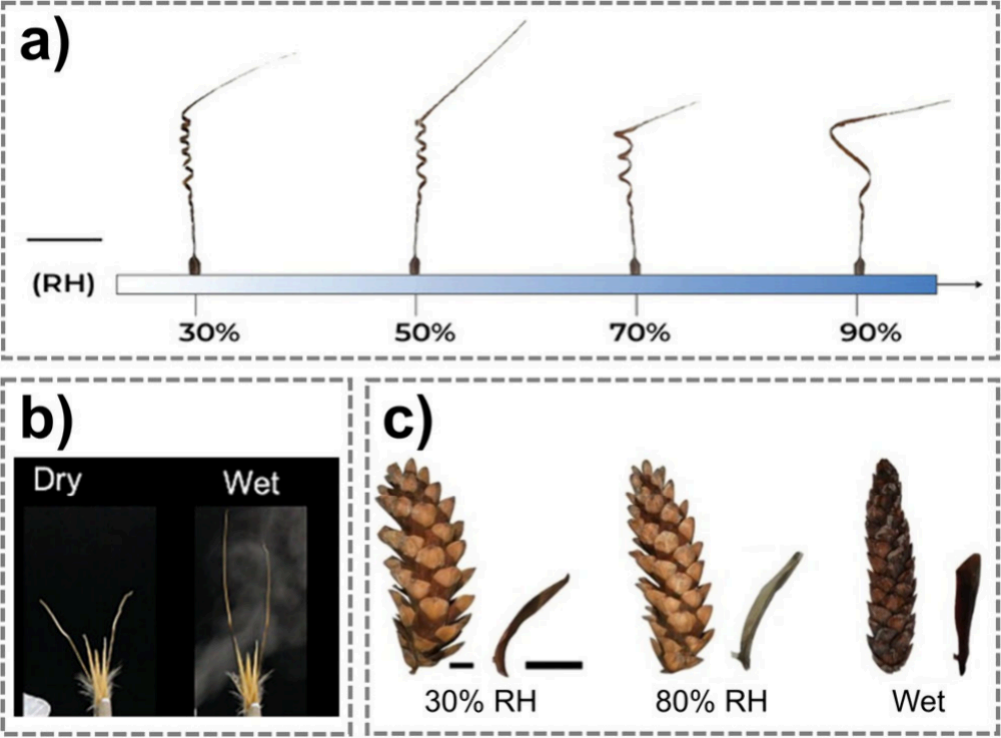
a) *Pelargonium appendiculatum* seed uncoiling with the increase of the RH from 30% to 90%. Scalebar is 1 cm. Reprinted with permission from ref 192 under CC BY 4.0, Copyright 2023 The Authors . b) *Avena sterilis* fruit in dry and wet conditions after using a humidifier. Reprinted with permission from ref 195 under CC BY 4.0, Copyright 2024 The Authors. c) The *Pinus wallichiana* cone at different levels of humidity. Open cone at 30% RH (at 23 °C) and the corresponding bent shape of the scale in lateral view. After 7 h at 80% RH (at 23 °C), the cone is slightly closed. The single scale shows a slight bending toward the cone axis. A cone soaked in water will close completely. The individual scale is completely straight upright. Scale bars are 2 cm. Reprinted with permission from ref 193 under CC BY 4.0, Copyright 2022 The Authors.

Inspired by these biological strategies, in the past decade, many reconfigurators, actuators, and soft robots that harness energy from environmental RH changes have been developed.^194^ These soft robots typically adsorb and desorb vapor water molecules on hydrophilic structures, converting this physical-chemical process into mechanical energy.^194^ Water adsorption causes expansion, which, combined with less active materials, induces shape changes like bending or twisting.^194^ In the following paragraphs, we will review the materials most used for the design and development of actuators and robots driven by humidity variations, focusing on hygroscopic expansion properties, mechanical properties, and manufacturing techniques for integration into soft robots.

### 3.1 Hygroscopic Materials

The natural hygromorphic structures mentioned above have been a significant source of bioinspiration for the design and development of hygroscopic actuators^194^ and soft robots solely powered by RH variations. For such actuation, the material must be hygroscopic, which is the reversible absorption and release of water to drive material volume change. Hygroscopicity represents a subset of hydrophilicity, where the distinction lies in the chemical structure. Hygroscopic materials not only possess hydrophilic functional groups (e.g., hydroxyl, carboxyl, and amine) that strongly bind water, but also have a molecular architecture that permits water uptake into the bulk. This combination of water affinity and capacity for molecular chain-level swelling is the key that enables humidity-driven actuation of hygroscopic materials.

Accordingly to the exhaustive classification reported by Tang et al.,^194^ hygroscopic materials to be integrated into soft robot structures can be classified as natural, naturally derived, or synthetic materials. In the following sections, we will systematically review the most used hygroscopic materials that have already been integrated into soft robots, or at least into hygroscopic actuators with potential application in soft robots. In addition to highlighting their chemical properties (functional groups and solubility), we also focus on physical and mechanical (e.g., Young’s modulus), and environmental (biodegradability, toxicity) properties of hydrophilic materials, along with the most widely used fabrication techniques. Particular attention is given to the humidity-responsive behavior, described by the coefficient of hygroscopic expansion (CHE) and the swelling ratio (SR). The CHE expresses the volumetric (or linear) expansion per unit of absorbed moisture and is often denoted as β, with a unit of % strain per %RH.^196^ The SR refers to the fractional increase in weight due to water absorption.^197^ Together with mechanical performance, these parameters are crucial for the design, modeling, and implementation of hygroscopic actuators and soft robots. Furthermore, evaluating biodegradability and toxicological profiles is critical for developing devices (e.g., soft crawlers) that can autonomously disperse into the environment for applications such as ecological monitoring and reforestation.^198,199^

#### 3.1.1 Natural Hygroscopic Materials

##### 3.1.1.1 Wood

Among the many naturally occurring hygroscopic materials, wood is an outstanding candidate for the development of hygroscopic actuators and soft robots. It is the structural tissue found in the xylem of tree trunks, branches, and roots, as well as in other woody plants and seeds. From a chemical point of view, wood is a natural composite made of cellulose and hemicellulose (carbohydrates), which provides tensile strength, and is embedded in a lignin matrix, enhancing resistance to compression.^200^ Its chemical composition varies by tree and plant species, but generally consists of lignin (18%–35%) and carbohydrates (65%–75%).^200^ Likewise, the Young’s modulus depends on the species, ranging from 10 to 60 GPa (in the longitudinal direction) when dry.^201^ For the wood processing steps for application in soft robotics, the most common approach involves the formation of wood veneers for the assembling of laminates, and delignification for reducing the mechanical strength, making the sheet compliant for mechanical molding^199^ and porosity tailoring. Alternatively, wood fibers can be included in 3D printable composites with thermoplastic materials (e.g., PLA and PCL) to provide significant flexibility in terms of design and the capability of forming 3D structures.^202^ From an environmental impact perspective, wood is optimal considering that it is naturally occurring and can be decomposed by a variety of biological agents, including fungi, bacteria, and insects.^203^

##### 3.1.1.2 Other Natural Materials

Besides wood, other natural hygroscopic materials with potential in humidity-driven soft robotics include: i) pollen, which has a rigid outer sporin layer with ester and acetal moieties and an inner layer with cellulose; ii) *bacillus* spores that can expand and contract in response to fluctuations in RH, iii) two-dimensional (2D) nanoclays, in particular Morillonite (Mt) that consists of SiO_2_, Al_2_O_3_ and other oxides.^194^ Similar to wood, materials derived entirely from natural sources offer the advantage of low environmental impact, as they are often renewable, biocompatible, (bio)­degradable, and with minimal processing and energy requirements.

#### 3.1.2 Naturally Derived Hygroscopic Materials

Hygroscopic materials derived and/or processed from natural polysaccharides (e.g., cellulose, agarose, chitosan, and alginates) are certainly the most widely used for the development of humidity-driven soft actuators and robots. The good hydrophilicity of these polysaccharides is due to the presence of -OH, -NH_2_, and -COOH groups.^194^ The following paragraphs will explain the representative naturally derived hygroscopic materials in detail.

##### 3.1.2.1 Cellulose

Cellulose is an unbranched homopolysaccharide composed of long chains of β-*D*-glucopyranose units connected by β(1→4) glycosidic bonds. Although the numerous hydroxyl groups along its molecular chains enable strong interactions with water, cellulose remains insoluble due to the extensive hydrogen bonding within its crystalline regions.^204^ It is soluble in ionic liquids, *N*-methylmorpholine-*N*-oxide (NMMO), LiCl/dimethylacetamide (DMAc), tetra­(*n*-butyl)­ammonium hydroxide/dimethyl sulfoxide (TBAH/DMSO), alkali/urea aqueous solution, and sulphuric acid solution.^205^ The Young’s modulus of microcrystalline cellulose was estimated to be 25 GPa,^206^ while the reported CHE of the amorphous cellulose is 0.0097.^207^ Cellulose is biodegradable by a variety of microorganisms with enzymes known as cellulases, which are secreted by cellulolytic bacteria and fungi. These enzymes are categorized into two main types: endoglucanases, which hydrolyze the β-1,4-glycosidic bonds found in amorphous cellulose, and cellobiohydrolases, which target the end groups of cellulose chains.^208^ In terms of toxicity, microcrystalline cellulose is considered safe for all animal species.^209^

##### 3.1.2.2 Carboxymethyl Cellulose (CMC)

CMC is a linear polysaccharide derived from a chemical modification of cellulose.^210^ In CMC’s chemical structure, the carboxymethyl groups (-CH_2_-COOH) are bound to the hydroxyl groups of the glucopyranose chain of cellulose. CMC is often used as a sodium salt (Na-CMC) with a solubility in water of 26–55 mg/mL.^211^ Its Young’s modulus is roughly 150–200 MPa (in film form, highly dependent on the moisture and plasticizer content)^212,213^ while the SR range from 10% to 45%.^214^ CMC-based films can be produced by solution casting or electrospinning.^215^ As for the environmental aspects, CMC is biodegradable in soil in the presence of microorganisms with hydrolytic cellulase enzymes^216^, and it is used as a food additive (E466 or E469) as an emulsifier and viscosity stabilizer.^217^ Toxicity experiments in rats revealed a LD_50_ of 27000 mg/kg after oral administration, indicating very low toxicity.^218^

##### 3.1.2.3 Cellulose Nanocrystals (CNCs)

CNCs are cellulose-derived micro- and nanomaterials with morphologies such as microcrystals, whiskers, nanocrystals or nanoparticles.^219^ They are typically isolated from cellulose fibers by acid hydrolysis (e.g., sulfuric acid), followed by ultrasonication and purification.^219^ CNCs range from 100–3000 nm in length and 5–50 nm in width, depending on synthesis conditions.^219^ Sulfuric acid hydrolysis introduces sulfate ester groups onto the cellulose surface, inducing surface charge and enhancing aqueous dispersion up to concentrations of 30–100 mg/mL. CNC colloids and hydrogels can be processed by casting, spin-coating, 3D printing, and electrospinning.^220^ The Young’s modulus in the longitudinal direction of the crystalline region in CNCs is ∼ 150 GPa,^221^ while CNCs also exhibit strong anisotropy. Literature value of CHE for self-organized CNC film is about 0.040.^222^ CNCs’ biodegradability is similar to cellulose,^223^ as microbial enzymes (cellulase and cellobiase) can accelerate their degradation in soil within weeks to months.^224^ Toxicity studies in rats report an oral LD_50_ of 5000 mg/kg.^225^

##### 3.1.2.4 Alginate

Alginate is a naturally occurring polysaccharide from brown seaweeds, composed of β-*D*-mannuronic (M block) and α-*L*-guluronic (G block) acid units linked by 1,4-linkages.^226^ As a polyanion, it readily forms gels through cross-linking with divalent cations (e.g., Ca^2+^).^226^ Its solubility in water depends on pH, ionic strength, cosolvents, and ions: alginic acid is insoluble, whereas sodium, potassium, and ammonium alginate salts are water-soluble,^227^ with solubility about 10–40 mg/mL.^228^ For mechanical properties, alginate films show a Young’s modulus of ∼ 1.9 GPa.^229^ Metal ion cross-linking enables the fabrication of alginate-based scaffolds, hydrogels, fibers, films, and membranes using techniques such as freeze-drying, microfluidic spinning, wet/jet spinning, and electrospinning.^230^ As for the environmental aspect, alginate is nontoxic, biodegradable, and can be degraded by alginate lyases.^231^

##### 3.1.2.5 Chitosan

Chitosan is a linear polysaccharide of randomly distributed β-(1→4)-linked *D*-glucosamine and *N*-acetyl-*D*-glucosamine units.^232^ It is obtained from chitin (crab, shrimp, lobster shells) via demineralization with HCl and deproteination with NaOH.^232^ Chitosan is insoluble in water and most solvents but dissolves in dilute acidic solutions (pH < 6.5), with acetic acid commonly used for solubilization.^233,234^ Chitosan films can be prepared by casting, coating, extrusion,^235^ or electrospinning,^236^ typically exhibiting brittleness, with tensile strength ∼ 40 MPa, Young’s modulus ∼ 1.6 GPa, and elongation at break ∼ 4%.^237^ Considering the environmental impact, chitosan is biocompatible and biodegradable, with chitin-degrading enzymes identified in a wide range of organisms, including fungi, bacteria, archaea, rotifers, algae, carnivorous plants, and even within the digestive tracts of animals.^238^ Finally, chitosan shows low toxicity (oral LD_50_ in mice: 16000 mg/kg).^239^

##### 3.1.2.6 Agarose

Agarose is a linear polysaccharide from red seaweed and one of the main structural components of agar, obtained by removing agaropectin. Its polymer chain consists of alternating *D*-galactose and 3,6-anhydro-*L*-galactopyranose units linked by α-(1→3) and β-(1→4) glycosidic bonds.^240^ Used as a gelling agent since the 17th century, it dissolves in boiling water (∼10 mg/mL)^241^ but not in cold water, and is also soluble in polar aprotic solvents such as DMSO, dimethylformamide (DMF), formamide (FA), *N*-methyl formamide (MFA), and certain ionic liquids.^242^ Agarose hydrogels show Young’s moduli ranging from ∼ 130 kPa (1% gels) to ∼ 3 MPa (10% gels).^243^ Additionally, agarose films can be fabricated by electrospinning.^244^ For environmental concerns, agarose is nontoxic and biodegradable, with degradation catalyzed by agarolytic enzymes including α-agarase, β-agarase, α-neoagarobiose hydrolase, and β-galactosidase.^245^

In addition to polysaccharides, moisture-sensitive peptide polymers such as silk fibroin (SF) and sericin, extracted from silk, have been employed in the construction of actuators and soft robotic systems. Silk is a natural fiber produced by various arthropods (e.g., spiders, scorpions) and insects such as bees and silkworms.^246^ Among these, mulberry silk from *Bombyx mori* L. is most widely used. It is primarily composed of SF (65%–85%) and sericin (15%–35%), the two proteins that provide hygroscopic functionality. The amorphous hydrophilic regions and α-helices of these proteins readily form hydrogen bonds with water, enabling strong moisture uptake and swelling.

##### 3.1.2.7 Silk fibroin

SF consists of a heavy (H, ∼ 390 kDa) and light (L, ∼ 26 kDa) peptide chain linked by a disulfide bond at the H-chain C-terminus, together with a glycoprotein P25 (∼25 kDa) that associates noncovalently in a ratio of H:L:P25 = 6:6:1 to form the silk complex. The amino acid composition of *Bombyx mori* SF is dominated by glycine (43%), alanine (30%), and serine (12%).^247^ SF represents the structural protein of silk and is isolated by degumming, a thermo-chemical process that removes the adhesive sericin.^247^ While insoluble in water and most organic solvents, SF swells by ∼ 30%–40%, largely in the amorphous region.^248,249^ It can be dissolved in solvents such as NMMO, hexafluoro-isopropanol (HFIP), hexafluoroacetone (HFA), or aqueous salt solutions (CaCl_2_, LiBr).^250^ SF coatings and fibers are fabricated by solvent casting, sol–gel processes, electrospinning, and wet spinning.^250^ Native SF fibers exhibit the following mechanical properties: elongation at break 13%–14%, elastic modulus 15–18 GPa, yield strength ∼ 230 MPa, tensile strength 620–760 MPa, and shear modulus ∼ 3 GPa.^250^ As a protein, the biodegradation of SF proceeds via proteolytic enzymes (protease, α-chymotrypsin, collagenase), yielding amino acids as end products.^250^

##### 3.1.2.8 Sericin

Sericin is a hydrophilic adhesive protein that binds SF fibers and is usually removed during degumming.^251^ Composed of 18 amino acids with up to 33% serine, sericin is highly water-soluble (solubility up to 90% at 90 °C).^252^ Its solubility and amino acid functionality make it attractive for developing biomaterials and bioproducts, including fibers, films, hydrogels, and 3D scaffolds.^251^ The Young’s modulus of sericin, estimated by the composite rule-of-mixtures in silk, ranges from 0.84–1.84 GPa.^253^ Sericin is also biodegradable, which can undergo enzymatic degradation both in vitro and in vivo by proteases such as protease XIV, α-chymotrypsin, proteinase K, papain, matrix metalloproteinases, and collagenase.^254^

#### 3.1.3 Synthetic Hygroscopic Materials

In addition to completely natural or naturally derived materials, hygroscopic polymeric materials resulting from organic polymerization can be used for the realization of humidity-driven actuators and soft robots:^194^

##### 3.1.3.1 Liquid Crystalline Polymers (LCPs)

LCPs consisting of polymerized mesogenic molecular network rich in -COOH groups are hygroscopic and processable in 1D fibers, 2D films, and complex 3D shapes.^194^ They typically exhibit higher modulus (in the GPa range) than LCEs (reported in Section 2.1.2), but the modulus can decrease under humidity conditions due to water uptake. The biodegradability of LCPs is often limited, while partial degradation through enzymatic hydrolysis of ester linkages is possible in some formulations.

##### 3.1.3.2 Polyvinyl Alcohol (PVA)

PVA is a polymer consisting of linear vinyl alcohol and is synthesized by the hydrolysis of polyvinyl acetate.^255^ It exhibits tunable solubility (e.g., solubility of 40–50 mg/mL for PVA with M_w_ 31 000–50 000, 87%–89% hydrolyzed)^256^ and mechanical properties (1–10 GPa)^257^ depending on molecular weight (20 000–400 000) and hydrolysis degree. It swells up to 231% in water and can be processed by casting, coating, 3D printing, or electrospinning.^258^ The biodegradation of PVA in soil is possible but slow (∼10% mineralization after 50 days for 72.5% hydrolyzed PVA), whose rate also reduces as the degree of hydrolysis increases.^259^ However, PVA can be considered environmentally safe and also edible^260^ with a very low acute toxicity: LD_50_ in the range of 15000–20000 mg/kg.^261^

##### 3.1.3.3 Poly­(3,4-ethylenedioxythiophene)-poly­(styrenesulfonate) (PEDOT:PSS)

PEDOT:PSS is a conductive composite^262^ with a reported Young modulus of 0.8 to 2.4 GPa.^263^ The hydrophilic group in PEDOT:PSS is -SO_3_^–^, usually neutralized by Na^+^, and it is generally in a water solution with a concentration ranging from 1%–4%.^264^ PEDOT:PSS has a swelling ratio of 60%, and it is processed by solution-processing techniques, such as spin coating, slot die coating, doctor blade, spray deposition, screen printing, andinkjet printing for thin film formation.^262^ This composite is not considered biodegradable,^265^ and it shows an acute toxicity LD_50_ of 2.000 mg/kg.^266^

##### 3.1.3.4 Perfluorosulfonic Acid Ionomer (PFSA)

PFSA, known as Nafion (commercial name), is an ion-conductive polymer with remarkable ion conductivity (0.015–0.035 S/cm at 30–60 °C, with RH = 70%, respectively)^231^ and chemical-mechanical stability, rich in -SO_3_H groups.^267,268^ Its Young’s modulus changes under various temperatures and humidities, from 59 to 197 MPa.^269^ Nafion is soluble in water and alcohols, with a SR of 10%–28%.^270^ For fabrication, PFSA thin films can be prepared by casting from diluted dispersion onto substrates via various methods (spin coating, self-assembly, drop casting, doctor blade, etc.).^267^ PFSA is not biodegradable and not environmentally friendly due to the presence of fluorinated compounds.^271^

##### 3.1.3.5 Polypyrrole (PPy)

PPy is another conductive^272^ material obtained by oxidative polymerization of pyrrole.^273^ It interacts with water through -NH groups and is mainly used in composite hygroscopic systems. PPy has good solubility in *m*-cresol, DMSO, DMF, and *N*-methyl-2-pyrrolidone (NMP).^239^ PPy films can be prepared by in situ chemical polymerization or by coating techniques (spin or electro coating),^273^ and their Young’s modulus can span from 0.53–4.32 GPa.^274^ In general, PPy is biocompatible but not biodegradable, and it is considered relatively nontoxic.

##### 3.1.3.6 Polyethylene Oxide (PEO)

PEO, also known as polyethylene glycol (PEG), depending on molecular weight, is a linear polyether obtained by polymerization of ethylene oxide.^275^ The polymer is strongly hygroscopic, and its properties vary with chain length. It is highly soluble in water (100–500 mg/mL), with a Young’s modulus spanning from 30 to 600 MPa,^276^ and a CHE of 0.076.^277,278^ For the fabrication of soft hygroscopic actuators, PEO fibers can be produced by electrospinning.^277^ Biodegradability of PEO is molecular-weight dependent: polymers up to ∼ 20 kDa are enzymatically degradable by soil microorganisms, whereas higher molecular weight grades are not biodegradable but remain environmentally neutral and safe.^279^ Its acute toxicity is very low, with an LD_50_ above 4000 mg/kg in rats for PEG 4000 kDa.^280^

In addition to the abovementioned materials, other hydrophilic synthetic polymers such as carbon nitride polymers, polyacrylamide (PAAm), and poly­(ethylene glycol) diacrylate (PEGDA) have also been employed for the preparation of humidity-driven actuators and robots.^194^ Besides polymers, hydrophilic 2D materials (e.g., graphene oxide and MXenes) can also serve as responsive elements in devices that react to humidity variation.

##### 3.1.3.7 Graphene Oxide (GO)

GO is a 2D material derived from graphite by chemical oxidation, followed by dispersion and exfoliation in water or suitable organic solvents.^281^ Unlike pristine graphene, GO is highly hydrophilic due to the abundance of oxygen-containing functional groups (-OH, 1,3-ether, ketone, quinone, and phenol) located on its surfaces and edges,^281^ which strongly interact with water molecules. GO is stably dispersible in both water (1–3 mg/mL) and various organic solvents.^282^ Its mechanical properties are outstanding: the Young’s modulus of a GO monolayer is 208 GPa (thickness ∼ 0.7 nm),^283^ while in the bucky paper form (thickness 1–30 μm), GO has a Young’s modulus of 32 GPa.^284^ In hygroscopic actuators, GO is most often used as a reinforcing or functional filler in composites with elastomers or hydrophilic polymers. Manufacturing routes for GO-based materials include additive manufacturing, electrospinning, casting, molding, and coating.^285,286^ In terms of sustainability, GO can be partially biodegraded by plant and animal peroxidases, including myeloperoxidase (MPO), making it environmentally friendly.^287^

##### 3.1.3.8 MXenes

MXenes are a class of 2D transition-metal carbides, nitrides, and carbonitrides discovered in 2011. They consist of few-atom-thick layers with the general formula M_*n*+1_X_*n*_T_*x*_ (*n* = 1–4), where M is a transition metal (groups 3–6), X is carbon and/or nitrogen, and T_x_ denotes surface terminations such as -O, -OH, -F, or -Cl.^288^ MXenes are typically produced via top-down etching of MAX phases (where A is an element from groups 13–16) using HF and/or HCl. Their surface functional groups render them strongly hydrophilic, allowing water dispersibility up to 25 mg/mL^289^ and very high SRs (∼1800% at 100 μg/mL).^290^ A monolayer of Ti_3_C_2_T_x_, the most widely studied MXene, has a nanomechanical Young’s modulus of 0.484 TPa.^291^ A multilayered MXene film can exhibit Young’s modulus from 28 to 72 GPa,^292,293^ depending on the film compactness and interlayer bonding. Like GO, MXenes are commonly incorporated into composites for humidity-driven actuation, with processing techniques including 3D printing,^294^ electrospinning,^295^ casting, and coating. Interestingly, MXenes also possess excellent electrical and thermal conductivities and photothermal conversion efficiency, making them suitable for two-step energy conversions, where external stimuli such as light, heat, or electricity modulate water adsorption and desorption, ultimately triggering hygroscopic motion in actuators.^296^ Partial enzymatic biodegradation of MXenes can be achieved by using horseradish peroxidase + H_2_O_2_, which produces TiO_2_ and CO_2_.^297^ While pristine MXenes are toxic to aquatic organisms, this toxicity decreases after degradation, albeit with an increased bioaccumulation of Ti.^297^

### 3.2 Material Selection Rationale

Table 3 summarizes the representative physical, mechanical, and environmental properties of the abovementioned hygroscopic materials, together with their typical fabrication processes. In Table 3, most physical and mechanical properties are presented as intrinsic to the material; however, they can be markedly altered through morphology engineering, for example, by introducing aerogel, hydrogel, or other 3D porous architectures. Therefore, both intrinsic material properties and morphology must be considered when selecting materials for the design of hygroscopic soft robots.

**Table 3:** Summary of Representative Properties of Hygroscopic Materials^a^

**Material**	**Solubility**	**Young’s modulus**	**CHE or SR**	**Fabrication process**	**Biodegradability and toxicity**
**Natural hygroscopic materials**					
**Wood**	Not soluble in water	10–60 GPa^201^ when dry	SR ≈ 6%–10%^302^	Used for veneer formation and mechanical molding,^199^ or used as fibers in thermoplastic composites for 3D printing.^202^	Biodegradation by fungi, bacteria, and insects;^203^ safe without toxicity.
**Naturally derived hygroscopic materials**					
**Cellulose**	Soluble in ionic liquid, NMMO, LiCl/DMAc, TBAH/DMSO, alkali/urea solution, sulphuric acid solution^205^	25 GPa^206^ when dry	CHE = 0.0097 ^207^ in amorphous cellulose	Electrospinning^303^	Biodegradation by microorganisms with enzymes known as cellulases;^208^ safe without toxicity.
**CMC**	Solubility in water: 26–55 mg/mL^211^	150–200 MPa in plasticized film forms^212,213^	SR ≈ 10%–45%^214^	Casting, coating, and electrospinning^215^	Biodegradation by microorganism with cellulases;^216^ LD_50_: 27000 mg/kg, oral administration in rats.^218^
**CNC**	Stable dispersion in water: 30–100 mg/mL	∼150 GPa in the crystalline region^221^	CHE ≈ 0.040 ^222^	Casting, coating, spin-coating, 3D printing,^220^ and electrospinning	Biodegradation by microorganism with cellulase and cellobiase;^224^ LD_50_: 5000 mg/kg, oral administration.^225^
**Alginate**	Sodium alginate (SA)’s solubility in water: 10–40 mg/mL^228^	1.9 GPa^229^	*NR*	Ion cross-linking, freeze-drying, wet spinning, and electrospinning^230^	Biodegradation by microorganisms with alginate lyases;^231^ nontoxic.
**Chitosan**	Slightly soluble in aqueous acidic solutions (pH < 6.5),^234^ soluble in acetic acid: 0.1 M or 1%^233^	1.6 GPa^237^	*NR*	Spread coating, spray coating, direct casting, extrusion,^235^ or electrospinning^236^	Biodegradation by chitin-degrading enzymes;^238^ LD_50_: 16000 mg/kg, oral administration.^239^
**Agarose**	Soluble in boiling water (10 mg/mL)^241^ and soluble in aprotic solvents^242^	0.13 and 3 MPa, for 1% and 10% hydrogels, respectively^243^	*NR*	Electrospinning^244^	Biodegradation by agarolytic enzymes;^245^ nontoxic.
**SF**	Soluble in HFIP, NMMO, HFA or in aqueous salt solution (CaCl_2_ and LiBr)^248^	15–18 GPa^250^ when dry	SR ≈ 30%–40% ^248,249^	Solvent casting, sol–gel procedures, electrospinning, and wet spinning for the deposition of fibers and nanofibers^250^	Biodegradation by proteolytic enzymes;^250^ nontoxic.
**Sericin**	Soluble in water until 90% at 90 °C^252^	0.84–1.84 GPa^253^	*NR*	Electrospinning, wet spinning, solvent casting, 3D bioprinting, and freeze-drying to produce fibers, films, and 3D scaffolds^251^	Biodegradation by proteolytic enzymes;^254^ nontoxic.
**Synthetic hygroscopic materials**					
**PVA**	Solubility in water: 40–50 mg/mL for M_w_ 31 000–50 000, 87%–89% hydrolyzed^256^	1–10 GPa^257^	SR = 231%^304^	Solvent casting, 3D printing, electrospinning, and nanofibers production^258^	Possible biodegradation in soil, but slow;^259^ LD_50_: 15000–20000 mg/kg, oral administration.^261^
**PEDOT:PSS**	Stable dispersion in water: 1%–4%^264^	0.8–2.4 GPa^263^	SR = 60%^305^	Spin coating, slot die coating, doctor blade, spray deposition, screen printing, and inkjet printing for thin film formation^262^	Not biodegradable;^265^ acute toxicity LD_50_ of 2.0 mg/kg.^266^
**PSFA**	Solubility in water: 22.0%; methanol: 49.2%; ethanol: 45.0%^306^	59–197 MPa^269^	SR ≈ 10%–28%^270^	Spin coating, self-assembly, drop casting, doctor blade^267^	Not biodegradable;^271^ toxic due to fluorinated compounds.
**PPy**	Soluble in *m*-cresol, DMSO, DMF, and NMP^239^	0.53–4.32 GPa^274^	*NR*	In situ chemical polymerization, or by spin or electro-coating^273^	Not biodegradable;^307^ relatively nontoxic.
**PEO**	Solubility in water: 100–500 mg/mL	30–600 MPa^276^	CHE = 0.076 ^277,278^	Electrospinning^277^	M_W_ < 20 kDa: biodegradable;^279^ M_W_ > 20 kDa: not biodegradable but biocompatible;^308^ LD_50_ above 4000 mg/kg for PEG 4000 kDa, oral administration.^280^
**GO**	Stable dispersion in water: 1–3 mg/mL^282^	208 GPa in monolayer,^283^ 32 GPa in bucky paper form^284^	SR ≈ 40%–75%^298^	Used as fillers in composites, processed by additive manufacturing techniques, electrospinning, solvent casting, molding, and coating^285^	Partial biodegradation by peroxidases, including MPO;^287^ its toxicity is dose-, size-, and surface-chemistry-dependent.
**MXene**	Stable dispersion in water: 25 mg/mL^289^	484 GPa in monolayer,^291^ 28 to 72 GPa in multilayered film form^292,293^	SR ≈ 1800% at 100 μg/mL^290^	Used as fillers in composites, processed by 3D printing^294^ and electrospinning,^295^ coating, and casting	Partial enzymatic degradation by horseradish peroxidase + H_2_O_2_; toxic to aquatic organisms; toxicity reduces after degradation.^297^

a *NR* = Not Reported.

For the intrinsic material type, hygroscopic properties such as CHE and SR are the key parameters that influence humidity-driven actuation performance. CHE and SR reflect the equilibrium extent of water-induced expansion of a hygroscopic material, meaning how much ultimate deformation the material can generate with respect to a given RH change. In addition, the kinetic moisture adsorption rate plays a critical role in defining the actuation response speed, but this parameter is rarely reported quantitatively across different hygroscopic materials. As a kinetic process, it is highly dependent on environmental conditions and sample geometry, so typically only qualitative comparisons are possible. In general, the adsorption rate is related to the density of hydrophilic surface groups and the specific surface area. For example, MXene has much higher SR (∼1800%)^290^ than GO (40%–75%)^298^ owing to the loosely stacked lamellar structure, yet GO exhibits a faster adsorption rate due to its richer oxygen functionalities. Thus, both equilibrium (CHE, SR) and kinetic (adsorption rate) aspects must be evaluated in material selection for hygroscopic actuators. In addition to the hygroscopic response, the mechanical properties of the material influence actuation performance. The Young’s modulus determines force output, but a higher modulus often correlates with brittleness, which compromises reversibility and fatigue resistance. Furthermore, biodegradability and toxicity are crucial concerns for actuators intended for natural or biomedical environments. From this perspective, naturally derived polysaccharides such as CMC, chitosan, and alginate offer a balanced combination of swelling capability, adsorption kinetics, and environmental sustainability. In contrast, 2D nanomaterials such as MXenes provide high-performance, fast-response, and multifunctional actuation, and can exploit additional stimuli (thermal, solar, or electrical inputs) to accelerate water desorption, thereby improving full-cycle actuation speed.

While intrinsic properties establish the fundamental limits of hygroscopic response, proper morphology design can amplify this behavior. Introducing porosity, hierarchical structures, or composite architectures modifies swelling by adding contributions from capillary condensation, enhanced diffusion pathways, and increased interfacial surface area. For example, CMC or chitosan in hydrogel or aerogel forms can swell significantly higher than their dense film forms due to water uptake into the porous network.^299^ Similarly, MXene and GO aerogels demonstrate far greater water adsorption compared to their dense lamellar counterparts, owing to the large specific surface area and open diffusion channels.^300,301^ Additionally, porous and nanostructured materials also enable faster adsorption–desorption cycles by shortening diffusion lengths through interconnected channels. However, highly porous structures are mechanically weaker than dense films, which may reduce actuation force output and fatigue life. Therefore, the design of hygroscopic actuators and soft robots requires balancing morphology-enhanced swelling behaviors with mechanical robustness for practical use.

### 3.3 Hygroscopic Soft Actuators and Robots

Hygroscopic actuators are commonly realized in a laminated composite form, where thin layers of hygroscopic and non-hygroscopic materials are combined to create an asymmetric composite. In this laminated form, the differential swelling between the layers translates RH changes into bending or twisting deformations. The mechanical behavior of such systems can be adapted from classical lamination theory, assuming each layer has orthotropic properties and perfect bonding between layers. Under this theory, the governing equation for the laminate curvature under hygroscopic loading is expressed as:^309^

$$\left[\frac{\left\{N\right\}}{\left\{M\right\}}\right]=\left[\frac{\left[A\right]}{\left[B\right]}\frac{\left[B\right]}{\left[D\right]}\right]\left[\frac{\left\{\varepsilon_{0}\right\}}{\left\{\kappa \right\}}\right]-\left[\frac{\left\{N^{h}\right\}}{\left\{M^{h}\right\}}\right]$$

where {*N*} and {*M*} are the resultant force and moment on the laminate, respectively; [*A*], [*B*] and [*D*] are the laminate extensional, coupling, and bending stiffness matrices, respectively; {*ε*_0_} and {κ} are the midplane strain and curvature of the laminate, respectively; {*N* ^h^} and {*M* ^h^} are the hygroscopic force and moment, respectively, which can be calculated based on the CHE of each layer and the%RH change. For free-standing actuators, the strain {*ε*_0_} and curvature {κ} can be calculated using {*N*} = {*M*} = 0. A more simplified version of this theory is the Timoshenko bimetallic strip model (equivalent to the thermo-responsive bilayer in Section 2.1.4), which assumes isotropic material properties and neglects twisting deformation:

$$\kappa =\frac{6\left(\alpha_{1}-\alpha_{2}\right)\Delta \phi }{\left(t_{1}+t_{2}\right){\left(1+t_{1}/t_{2}\right)}^{2}}\frac{E_{1}}{E_{2}}$$

where κ is the laminate curvature; α_,_ *t*, and *E* are the CHE, thickness, and Young’s modulus of each layer, respectively, as the layer number is distinguished by the subscript; *Δϕ* is the %RH change. From this simplified model, the resulting bending curvature is driven by the CHE mismatch of the layers and the %RH change, and can be modulated by the modulus and layer thickness. These two models describe the equilibrium deformation of hygroscopic actuators but do not capture the kinetics of water diffusion. In practice, diffusion through the bulk is orders of magnitude slower than heat conduction for thermal actuators (in Section 2), making the response time of hygroscopic bilayers highly thickness-dependent. Consequently, the thickness of hygroscopic actuators plays a much larger role in limiting actuation speed compared to thermal bilayers. To ensure practical response rates, most humidity-driven actuators are therefore fabricated as thin films, typically below 1 mm in thickness. Based on the laminated actuator design, various examples of reconfigurators, graspers, and soft robots driven by humidity variations have been reported in scientific literature.^194^ In the following paragraphs, we will review the most significant devices and milestones in hygroscopic soft robotic research.

Humidity-driven locomotion, including crawling and rolling, has been studied by researchers for more than one decade. Early demonstrations of hygroscopic locomotion relied heavily on 2D nanomaterials such as GO, due to its strong and fast moisture adsorption.^310^ In a pioneering 2013 study, Cheng et al. reported a fiber walker made of selectively reduced GO (G/GO), which moved between two glass slides via moisture-driven bending.^311^ At high humidity (RH = 80%), the fiber bent toward the reduced G side, and upon returning to ambient conditions (RH = 25%), it recovered the original shape. This reversible process enabled continuous walking with an average motion rate of ∼ 8° s^–1^. Building on the same principle of GO moisture adsorption, a centipede-like soft robot was developed using structured GO films patterned by soft lithography.^312^ Ten pairs of twisting “legs” coordinated their motion to propel the robot forward at a speed of 0.98 mm s^–1^, powered simply by humid air at a flow rate of 0.2–0.4 m s^–1^. To further enhance performance, hybrid nanomaterials were introduced through the combination of GO with carbon nanotubes (CNTs) and cellulose nanofibers (CNFs), which resulted in a conductive composite film via vacuum-assisted self-assembly.^313^ The porous nanostructure and the hydrophobicity of CNTs improved water exchange and desorption, which enabled rapid actuation (0.8 s for bending, 2 s for recovery) and stability over 1000 cycles. This composite was successfully integrated into a soft crawling robot that reached speeds of 2.6 mm s^–1^. A further step was taken with a humidity-responsive agarose/GO/polyvinylpyrrolidone (PVP) composite film, prepared by a simple casting method.^314^ The monolayer film exhibited a high angular response speed (124.58° s^–1^) and a recovery rate of 18.87° s^–1^. Leveraging this fast actuation, researchers demonstrated rolling and crawling robots powered by periodic water vapor, reaching locomotion speeds of 28.73 mm s^–1^ and 9.9 body lengths per minute (BL min^–1^), respectively.

Progress then expanded toward polymer-based hygroscopic actuators. A representative example is Hygrobot, reported in 2018, which employed aligned PEO fibers electrospun onto a polyimide (PI) substrate (Figure 7a, panel I).^278^ The highly porous microfiber network enhanced both CHE and water diffusion, leading to fast and sensitive actuation. Directional locomotion was achieved by coupling oscillatory bending with asymmetric surface friction (Figure 7a, panel II and III), enabling the robot (0.035 g mass, 30 μm active layer) to crawl at 6 mm s^–1^, corresponding to 0.24 BL s^–1^. The same electrospinning strategy for PEO was later adapted to polyvinyl chloride (PVC) substrates, yielding humidity-driven wheels, seesaws, and vehicle-like machines, further diversifying the locomotion modes achievable with hygroscopic polymers.^315^

**Figure 7:**
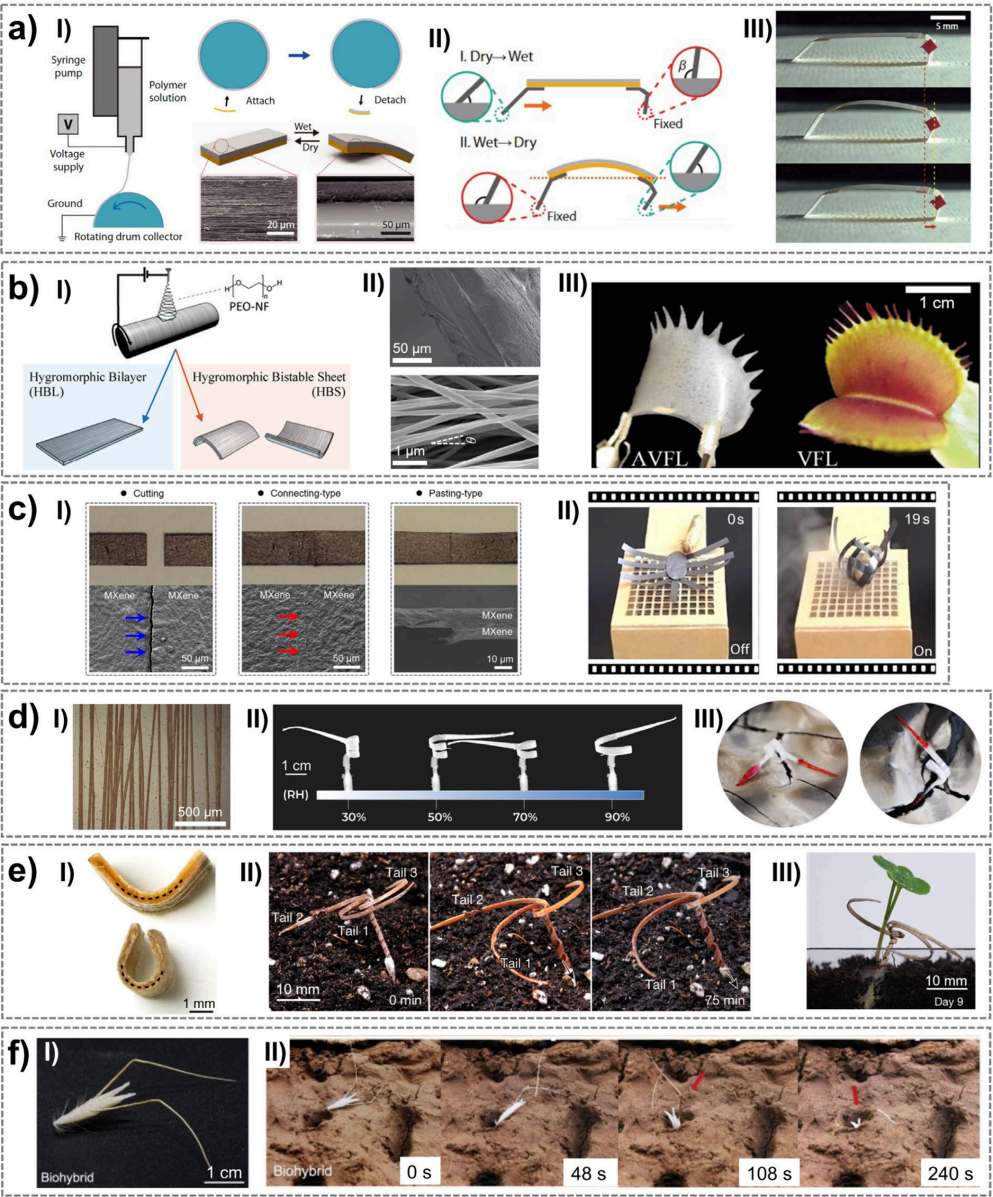
Soft robots driven by humidity variations. a) Hygrobot: I) fabrication process, schematic of the bending deformation of the bilayer, and SEM images; II) schematics of Hygrobot crawling spontaneously on a moist surface; III) movement of Hygrobot during a single period of humidity variation. Reproduced from Shin et al., Science Robotics, DOI: 10.1126/scirobotics.aar2629 [2018], AAAS.^278^ b) Bistable artificial *Venus* flytrap (AVFL): I) Electrospinning process for the hygroscopic bilayer and bistable sheet; II) SEM images of the cross-section of the bilayer (top) and the aligned nanofiber layer on PDMS (bottom); III) AVFL compared to the natural counterpart. Reprinted with permission from ref 277 under CC BY 4.0, Copyright 2020 The Authors. c) MXene/GO-based hygroscopic flytrap: I) self-healing capability of MXene films through water swelling-desorption; II) snapshots of the insect capture process, where the flytrap is powered by the humidifier that is triggered by a sandwiched MXene/GO sensor. Reprinted with permission from ref 316 Copyright 2021 Science China Press. d) Pelargonium-like soft robot: I) Microscope image of electrospun PEO/CNC fibers on a PCL substrate; II) experimental visualization of pitch and radius changes (coiling/uncoiling) of the soft robot as a function of RH; III) soil crack penetration after RH changes and autonomous random exploration. Reprinted with permission from ref 192 under CC BY 4.0, Copyright 2023 The Authors. e) Three-tailed wood-based seed carrier: I) the curvature of a wood veneer evolving from wet-released state (up) to dry state (down); II) snapshots of the three-tailed seed carrier during the first hydration cycle triggered by natural rain; III) the germination process of a three-tailed seed carrier with cherry belle radish seeds and mycorrhizal fungi. Autonomous self-burying seed carriers for aerial seeding, Luo et al., Nature, 614, Springer Nature, 2023, reproduced with permission from SNCSC.^199^ f) HybriBot: I) photo of HybriBot consisting of a biomimetic biodegradable capsule and two natural awns; II) HybriBot placed on artificial clay soil, while entering soil crevice. Reprinted with permission from ref 195 under CC BY 4.0, Copyright 2024 The Authors.

More recently, research has begun exploring emerging concepts such as multifunctional or self-healing hygroscopic materials, aimed at increasing durability and resilience. Cao et al. developed a humidity-responsive copolyester, synthesized through condensation copolymerization of PEG and poly­(tetramethylene glycol) (PTMG), which combined reversible shape change with intrinsic self-healing.^317^ Structural components of hygroscopic actuators were assembled by “welding” through reestablishment of hydrogen bonds at low ambient temperatures, eliminating the need for external adhesives. Exploiting this property, a crawler carrying payloads was built based on this hygroscopic material and beeswax as the inactive layer, which could move unidirectionally with a speed of 0.4 BL min^–1^ when the RH cycled between 20% and 90%. Such demonstrations highlight the potential of integrating repair and reconfigurability into hygroscopic locomotion systems to achieve more durable and adaptive soft machines. Beyond material choice, component geometry in composite laminates strongly influences the response time of soft actuators. Zhang et al. showed that pinecone hygroscopic motion is exceptionally slow because its vascular bundles contain spring-shaped and square microtube heterostructures.^189^ The spring-shaped tubes deform far more than the square ones, driving the overall bundle response. Inspired by this mechanism, the authors 3D printed resin-based actuators that moved almost imperceptibly, at ∼ 10^–5^ BL s^–1^, 2 orders of magnitude slower than other reported actuators.

Hygroscopic actuation has also been harnessed for grippers and graspers.^318^ Using electrospun PEO nanofibers deposited on prestretched PDMS substrates (Figure 7b, panel I and II), Lunni et al. developed a hygroscopic bistable sheet mimicking the fast snapping mechanism of *Venus* flytrap (carnivorous plant *D. muscipula*, Figure 7b, panel III*)*.^277^ Thanks to the bioinspired bistability, the grasper achieved rapid closure (∼1 s), significantly faster than conventional hygroscopic bilayers (∼10 s), enabling efficient moisture-driven grasping. Graspers have also been realized through the fabrication of multifunctional and self-healing nanocomposites. Ma et al. developed a moisture-induced self-assembly and self-healing of homogeneous and heterogeneous MXene/GO nanomaterials, allowing moisture-driven grippers to recover functionality after damage (Figure 7c, panel I).^316^ Such materials have enabled adhesive-free fabrication of diverse bioinspired robots, like flytrap-inspired graspers (Figure 7c, panel II), as well as small-scale architectures resembling ants, crabs, spiders, dragonflies, and geckos.

The integration of natural or biodegradable materials in hygroscopic soft robots represents an essential step toward real-world deployment. Unlike synthetic systems, biodegradable soft robots can operate in natural environments without raising concerns of long-term ecological footprint. This feature is especially valuable for applications where robots are designed to be dispersed in large numbers and may not be retrieved after use, such as environmental monitoring, agricultural sensing, or targeted delivery. Based on this concept, Cecchini et al. reported the first humidity-driven robot bioinspired by *Pelargonium appendiculatum* seeds (Figure 7d), which was fabricated through the coupling of 3D printing of PCL and coaxial electrospinning of CNCs (inner core) and PEO (coating).^192^ The artificial awn of the robot had a torque value ≈30 μN m, an extensional force ≈2.5 mN (similar to the natural one), and it was able to lift ≈100 times its own weight. The artificial hygroscopic seed actuators showed an energy density of 52.16 kJ m^–3^, a power-to-mass ratio of 154.38 μW kg^–1^, and an average moving speed of 0.63–0.93 mm/humidity cycle on a clay surface. It was conceived for random soil exploration driven by humidity changes and for perspective distributed and environmental monitoring (e.g., visual RH sensing).^319^ A more sustainable example based on purely naturally derived materials was an autonomous self-burying seed carrier for aerial seeding. This seed carrier (seeding robot) developed by Luo et al. had moisture-powered actuation mechanisms, which was made of delignified wood veneer (Figure 7e, panel I).^199^ The robot was characterized by an artificial three-tailed awn with a modulus of approximately 4.9 GPa when dry and around 1.3 GPa when wet, as well as a high bending curvature of 1854 m^–1^. The artificial hygroscopic awn was coupled with a biodegradable capsule made of flour and carried with a variety of payloads, including biofertilizers and plant seeds as germination models for aerial seeding purposes. The moisture-driven drilling and self-burying success rate was 80% on flat terrain after two triggering cycles (Figure 7e, panel II), thanks to its optimal tail anchoring angle of 25°–30°. This work was a clear example of how design and engineering of natural materials (e.g., wood veneers) can deliver outstanding mechanical performance. Similarly, Chen et al. demonstrated that bamboo films obtained by a chemical-free process could have a programmed response by carefully controlling the thickness. Under moisture stimulation, the bending angle was up to 300° in 9 s. If the thickness of the bamboo was 25 μm, rapid actuation and continuous locomotion were exhibited under moisture stimulation from human fingers (2 cycles) with a displacement of 0.5 cm.^320^ In addition to engineering robotic devices out of naturally derived materials, combining natural components with engineered structures is a new approach for sustainable robotic systems. As an example, Fiorello et al. developed a moisture-driven biohybrid self-dispersing miniature machine (HybriBot) directly integrating wild oat fruit (*Avena sterilis*) awns with a biomimetic flour-based capsule (Figure 7f, panel I), which was realized with a mold printed via two-photon polymerization (2PP). HybriBot could carry tomato seeds for perspective autonomous reforestation and precision agriculture purposes (Figure 7f, panel II).^195^

Most of the soft robots reported so far are capable of locomotion or reversible actuation only in response to temporal changes in humidity. To expand the application in constant-humidity environments, Fu et al. introduced a soft robot called Hydrollbot, which achieved rolling locomotion by leveraging the inherent humidity gradient between the two sides of its body when operating on a constant-humidity surface.^321^ The Hydrollbot was made with a hygroscopic agarose film linked to PET strips to avoid the random twisting of the isotropic agarose film. By harnessing evaporative energy, Hydrollbot demonstrated spontaneous, continuous, and rapid self-rolling locomotion with a programmable trajectory under uniform humidity conditions. Additionally, the geometric parameters of the robot were fine-tuned to maximize rolling speed (0.714 BL s^–1^), enabling the optimized Hydrollbot to carry a payload of up to 100% of its own weight.

In Table 4, we summarize the fabrication methods, functional materials, performance metrics, energetic sustainability classification, and applications of the representative humidity-driven soft robots. Because hygroscopic actuation arises from the direct and often quasi-linear response of materials to ambient relative humidity, most humidity-driven actuators and robots can operate entirely within naturally occurring humidity fluctuations and are therefore classified as fully sustainable. This intrinsic ability of hygroscopic actuators to operate in low-energy environments makes them particularly attractive for applications in adaptive architecture components, distributed environmental sensing, autonomous locomotion for exploration, and seeding devices. However, key challenges remain in purely humidity-driven systems, including limited predictability of environmental humidity, slow response times, and long-term material durability.

**Table 4:** State of the Art in Humidity-Driven Soft Actuators and Robots^a^

**Soft actuator and robot**	**Materials and fabrication process**	**Performance**	**Energetic sustainability classification**	**Applications**	**Ref**
**Hygrobot**	Bilayer. AL: aligned PEO fibers; IL: PI substrate.	Linear locomotion at a speed of 6 mm s^–1^, or 0.24 BL s^–1^ when RH change period = 2 s.	Fully sustainable	Locomotion: crawling	^278^
	Fabrication: electrospinning.	RH tested: 20%–80% at *T* = 25 °C.			
**Hygro-machines: wheels, seesaws, and vehicles**	Bilayer. AL: aligned PEO fibers; IL: PVC substrate.	Hygro-wheel: theoretical maximum speed of 26 mm s^–1^; hygro-seesaws: theoretical maximum frequency of 0.076 Hz.	Fully sustainable	Locomotion: diverse modes	^315^
	Fabrication: electrospinning.	RH tested: 20%–80% at *T* = 25 °C.			
**Centipede-like soft robot**	Bilayer. AL: GO film; IL: PDMS layer.	Speed: 0.98 mm s^–1^.	Fully sustainable	Locomotion: crawling	^312^
	Fabrication: soft lithography.	RH tested: 44%–97% at *T* = 25 °C.			
**Crawler robot**	Nanocomposite film of GO, CNTs, and CNFs.	Response time: 0.8 s; recovery time: 2 s; operating for 1000 cycles without degradation. Crawling speed: 2.6 mm s^–1^.	Fully sustainable	Locomotion: crawling	^313^
	Fabrication: vacuum-assisted self-assembly.	RH tested: 30%–70% at *T* = 30 °C.			
**Rolling and crawling robot**	Bilayer. AL: agarose and GO; IL: PVP film.	Response speed: 124.58° s^–1^; recovery speed: 18.87° s^–1^. Rolling speed: 28.73 mm s^–1^. Crawling speed: 9.9 BL min^–1^.	Fully sustainable	Smart bionic devices and locomotion devices: rolling and crawling	^314^
	Fabrication: casting.	RH tested: 35%–80% at *T* = 25 °C.			
**Bistable** *Venus* **flytrap**	Bilayer. AL: PEO nanofibers; IL: prestretched PDMS.	Grasping response time: ∼ 1 s.	Fully sustainable	Smart grasper	^277^
	Fabrication: electrospinning.	RH tested: 35%–85% at *T* = 25 °C.			
**Self-healing hygroscopic robot**	Bilayer. AL: GO; IL: MXene.	Bending response and recovery time: 13 and 9 s. Insect grasper actuation time: 8–16 s.	Fully sustainable	Smart grasper, reconfigurable robots	^316^
	Fabrication: self-assembly.	RH tested: 23%–100% at *T* = 25 °C.			
**3D printed pinecone actuator**	Hygroscopic resins.	Extremely low speed: 10^–5^ BL s^–1^.	Fully sustainable	Ultraslow shape morphing for camouflage	^189^
	Fabrication: 3D printing.	Tested by water immersion.			
***Pelargonium*-inspired soft robot**	Bilayer. AL: PEO and CNCs; IL: PCL.	Torque ≈ 30 μN m; extensional force ≈ 2.5 mN. Lift ≈ 100 times its own weight. Energy density of 52.16 kJ m^–3^.	Fully sustainable	Random soil exploration and environmental monitoring, with biodegradability	^192^
	Fabrication: 3D printing and coaxial electrospinning.	RH tested: 30%–90% at *T* = 25 °C.			
**Autonomous self-burying seed carriers**	Delignified wood veneer.	Peak extension force: 55.18 mN; extension force in real environment (30 °C): 42.47 mN. Self-burying success rate on flat soil: 80%.	Fully sustainable	Autonomous aerial seeding and reforestation, with biodegradability	^199^
	Fabrication: chemical delignification, wood-forming.	RH tested: 30%–93% at *T* = 25–30 °C.			
**Bamboo crawler**	Bamboo 0.025 mm film (ultramicrotome).	Bending speed: 300° in 9 s. Crawling displacement of 0.5 cm under moisture stimulation of human fingers (2 cycles).	Fully sustainable	Biodegradable robot for locomotion, with biodegradability	^320^
		RH tested: 24%–89% at *T* = 28 °C.			
*Avena*-**inspired HybriBot**	Natural *Avena* awns integrated with a flour capsule.	Capsule drag forces ≈ 0.38 N; Awns torque ≈ 100 mN mm^–1^.	Fully sustainable	Aerial seeding and reforestation, with biodegradability	^195^
	Fabrication: casting from a mold manufactured via 2PP.	RH tested: 30%–98% at *T* = 25 °C.			
**Continuous self-rolling robot (Hydrollbot)**	Bilayer. AL: agarose film; IL: PET.	Rolling speed: 0.714 BL s^–1^.	Fully sustainable	Locomotion: rolling; medical robots	^321^
	Fabrication: casting.	RH tested: 40%–70% at *T* = 23–61 °C.			

a AL = active layer, IL = inactive layer.

A central limitation of current hygroscopic systems is the desorption/deswelling process, which typically governs the full-cycle actuation speed of highly hydrophilic actuators. Designing new structures that accelerate water release through multiscale architectures or porous scaffolds will be critical for improving cycle frequency. Meanwhile, integrating multifunctional materials into the hygroscopic actuator to harness other forms of environmental energy (e.g., thermal or solar) for accelerated water desorption can further improve the performance of humidity-driven robotic systems. Another promising research direction is the exploitation of spatial humidity gradients, rather than relying solely on temporal humidity fluctuations. By harvesting steady-state gradients, robots could maintain continuous operation independently of natural diurnal or seasonal cycles, overcoming one of the primary constraints of current designs.

Environmental humidity on Earth typically ranges between 20% and 80% RH in most outdoor conditions, though it can extend beyond these limits in arid deserts (<10%) or tropical climates (>90%). Although most existing demonstrations of hygroscopic robots can operate in this natural RH range, the more practical concern is their requirement of large humidity swings (ΔRH > 50%) to drive full actuation strokes. The diurnal swing of humidity can vary significantly in different climates, from 20% in tropical and up to 80% in desert regions, restricting the application areas of the existing robots. Bridging this gap requires materials and structures capable of amplifying small humidity fluctuations into effective mechanical work, or alternatively, designs that exploit localized gradients (e.g., between soil and air, or within confined architectures). In addition, sensor-feedback control could allow real-time adaptation, enabling the system to compensate for unexpected fluctuations and maintain consistent performance.^322^ These systems monitor deformation, pressure, and position in real time, enabling closed-loop regulation to counteract nonlinearities and disturbances. Control strategies include open-loop, closed-loop, and hybrid approaches, with feedback commonly provided by visual, force, and tactile sensors.^322^ Incorporating these strategies could be crucial for enhancing the robustness, operational lifespan, and practical applicability of humidity-driven soft robotic systems.

Finally, integrating biodegradable hygroscopic materials such as cellulose or wood derivatives would not only enhance mechanical performance but also enable sustainable deployment in ecological contexts. Such robots, which safely degrade after autonomous dispersion, are particularly well suited for large-scale environmental applications, including soil monitoring, agricultural automation, and reforestation.

## 4 Soft Robots Powered by Sunlight

In this section, we examine soft robots powered by sunlight. We first introduce solar radiation and the mechanisms by which natural systems can actuate in response to light exposure. We then explore photoresponsive materials that adapt to sunlight variations, concluding with a comparative analysis of soft robots powered by sunlight using photoresponsive materials and a discussion on solar energy harvesting.

The solar constant, approximately 1361 W m^–2^ (136.1 mW cm^–2^), represents the average solar irradiance on a surface oriented perpendicular to the Sun’s rays at the top of Earth’s atmosphere.^323^ This value is not strictly constant: it varies by about ± 3% annually due to Earth’s elliptical orbit and is further reduced at Earth’s surface by atmospheric effects. For terrestrial applications, the more relevant reference is the standard solar irradiance under AM1.5G conditions, commonly referred to as 1 sun, corresponding to approximately 100 mW cm^–2^. This terrestrial irradiance level, therefore, serves as a practical upper bound for sunlight-driven systems intended to operate effectively in outdoor environments. In natural outdoor environments, solar irradiance is inherently dynamic, exhibiting pronounced temporal fluctuations due to diurnal cycles and cloud cover. Rather than representing a limitation, such variability can be exploited as a useful energy input for dynamic or intermittent actuation modes in sunlight-driven systems. Nature offers rich inspiration, notably from plants and seeds that adaptively orient themselves to maximize sunlight absorption through phototropism,^324,325^ which drives an uneven distribution of growth factors like auxin in the stem. This auxin gradient causes cell elongation on the shaded side, enabling directional growth (Figure 8).^324^ Inspired by such adaptive strategies, engineered systems can similarly exploit fluctuating illumination, although achieving continuous and efficient operation typically requires complementary approaches, including energy storage, thermal buffering, or adaptive structural and control strategies.

**Figure 8:**
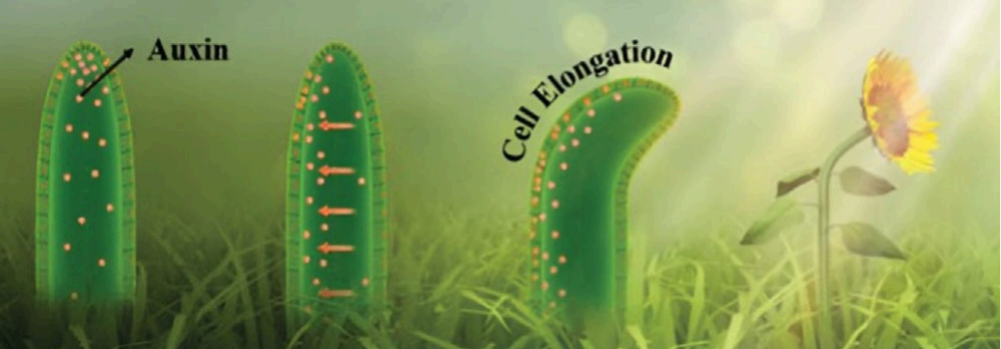
Schematic illustration of phototropism in sunflowers by a gradient of auxins and directional growth toward the sunlight. Reprinted with permission from ref 326 Copyright 2022 John Wiley and Sons.

### 4.1 Photoresponsive Materials

In soft robotics, photochemical and photothermal conversions function as material-embedded energy transduction mechanisms that enable direct, light-driven deformation and untethered actuation. The following subsections introduce representative materials employed in photoresponsive soft robots that utilize these two mechanisms.

#### 4.1.1 Photochemical Materials

The most explored photochemical mechanism that induces material deformation is photoisomerization.^327^ This approach is typically achieved by embedding light-responsive molecules into anisotropic soft materials such as LCEs, where nanoscale conformational changes in photoswitches or chromophores can be amplified into macroscopic material deformations. Several classes of photochromic molecules have been investigated for this purpose, including azobenzene derivatives, spiropyrans, and diarylethenes. Azobenzene systems, for example, exhibit a pronounced trans–cis isomerization between a planar and a bent state under ultraviolet (UV) or sunlight exposure,^328^ with the reverse process occurring thermally or under visible light; their compatibility with polymer matrices and reversible actuation make them attractive for repeated light-driven cycling. Spiropyrans and spirooxazines undergo UV-triggered ring-opening into extended merocyanine forms, reverting thermally or under visible light, and are valued for their strong photocolorability and stability. Diarylethenes undergo reversible switching between open- and closed-ring structures, with UV light inducing ring closure and visible light promoting reopening. Despite their chemical versatility and scientific appeal, the practical use of photoisomerization in sunlight-driven soft robots is limited. The main challenges stem from the mismatch between molecular absorption spectra and the solar spectrum: many photochromic molecules are activated by narrow UV bands or lights with specific wavelengths,^329^ which is only a very small portion (<10%) of the solar irradiance at the Earth’s surface. More importantly, reversible photoisomerization processes require two different wavelengths for the two-way actuation, which is not possible to achieve solely with broadband sunlight.^330^ Thus, photochemical materials are not suitable to be the building blocks for sunlight-driven soft robots.

#### 4.1.2 Photothermal Materials

In soft robotics, the most common approach to generate motion with sunlight exploits the photothermal properties of materials embedded in the soft robot structure, i.e., the ability to directly convert light energy into thermal energy.^331^ When the light-induced energy is converted into heat, it can trigger various actuation mechanisms for material deformation, such as thermal expansion or contraction, phase changes, nematic–isotropic transition, and crystalline structure transition (described in Section 2), or water adsorption/desorption (described in Section 3).^332^ Other effects, like the generation of surface tension gradients, can also contribute to actuation.^332^

Advancements in material science are central to the enhanced performance and functionality of photothermal actuators. The physical mechanism of photothermal conversion is based on plasmonic localized heating, nonradiative relaxation in semiconductors, and thermal vibrations of molecules. The efficiency of the photothermal effect is typically measured by photothermal conversion efficiency (PCE or η), which quantifies the amount of electromagnetic energy that is transformed into heat. It is possible to evaluate this coefficient considering its definition in thin layers as:^333^

$$\eta =\frac{\Delta T}{\tau }\frac{mC_{p}}{I_{0}\left(1-10^{-\alpha }\right)}$$

where *ΔT* is the difference in temperature generated in a time constant τ, when light impinges the thin film with optical power *I*_0_, *m* is the mass of the sample, *C*_p_ is the material heat capacitance, and α is the absorbance. Nanomaterials play a central role in enhancing photothermal performance owing to their exceptional light–heat conversion properties. The choice of photothermal materials should be focused on the intrinsic efficiency, the versatility in production and functionalization, and their impact on sustainability.^334^ Commonly employed photothermal materials in sunlight-driven soft robots include 2D materials such as MXenes and GO, nanomaterials such as CNTs and noble metal nanoparticles, and bioderived alternatives such as lignin.

MXenes are the most promising materials in this field. In MXenes, the photothermal conversion is primarily due to plasmonic-like behavior arising from the high electrical conductivity of the metallic transition metal layers and enabling efficient light absorption and subsequent heat generation. They represent the most promising materials in PCE, showing η ≈ 90%–100%.^335,336^ They exhibit excellent mechanical properties, including high stiffness and tensile strength, combined with flexibility (reported in Section 3.1.3). These properties make them suitable for coatings and composites. However, challenges in synthesis include the use of hazardous chemicals (e.g., HF) during etching and delamination,^288^ which complicates large-scale production and raises environmental concerns.

Alternatively, GO is a derivative of graphene that contains abundant oxygen functional groups, including hydroxyl, epoxy, and carboxyl groups, and has revealed great potential for practical application in photothermal actuation. Its structure consists of a single-layered 2D material with disrupted sp^2^-bonded carbon networks due to the oxygen groups. Detailed chemical, physical, and mechanical properties of GO have been previously reported in Section 3.1.3. Photothermal conversion in GO occurs through nonradiative relaxation processes facilitated by its defect states and strong absorption in the near-infrared (NIR) region, with η = 58%.^337^ These properties are linked to its oxygenated structure, which allows efficient photon absorption and heat release. GO demonstrates high mechanical strength and flexibility, making it ideal for applications like soft composites. A significant advantage of GO is its relatively low-cost and scalable production via chemical exfoliation of graphite. However, controlling the oxygen content and achieving uniform reduction during synthesis can be challenging, which may limit its mechanical performance.

Still carbon-based, CNTs are cylindrical structures composed of rolled-up graphene sheets, which can be single-walled (SWCNTs) or multi-walled (MWCNTs). They exhibit extraordinary mechanical properties, including high tensile strength (up to 100 GPa), excellent elastic modulus (∼1 TPa), and remarkable flexibility.^338^ These properties arise from the strong sp^2^ carbon-carbon bonds in their structure, making CNTs ideal for reinforcing materials in composites and various advanced applications requiring high specific strength. CNTs are incorporated into composites by dispersing them within a matrix material to enhance the mechanical properties. Proper functionalization of CNTs (e.g., surface modification) ensures strong interfacial bonding, enabling efficient load transfer. Techniques include solution mixing, melt blending, and in situ polymerization for uniform dispersion.^339^ Photothermal conversion in CNTs occurs through efficient photon absorption across a broad spectrum, particularly in the NIR range, followed by rapid nonradiative decay into heat due to their high electron density and conductivity. The PCE of CNTs ranges from 60% to 70%.^332^ Despite these advantages, large-scale production remains expensive and complex, involving processes like chemical vapor deposition (CVD) or arc discharge. Additionally, issues such as bundling and poor dispersion in matrices can compromise their performance in applications.

In addition to carbon-based materials, plasmonic metal nanoparticles (NPs), e.g., gold (Au) or silver (Ag) NPs, are nanoscale materials characterized by their strong localized surface plasmon resonance (LSPR) phenomena. This property arises from the collective oscillation of conduction electrons in response to incident light. The photothermal conversion mechanism is directly linked to LSPR, where absorbed light energy is converted into heat via electron–phonon interactions and phonon–phonon relaxation processes. These NPs possess excellent chemical stability and tunable optical properties by varying their size, shape, and composition. The PCE ranges from 40%–90% according to the selected metal, shape, and dimension.^334^ However, their mechanical properties are less relevant in most cases due to their nanoscale nature. Noble plasmonic NPs used in composites are commonly fabricated via chemical reduction, sol–gel synthesis, physical vapor deposition, and electrochemical methods. These techniques enable precise control of NP size, shape, and distribution. Moreover, to reduce the toxicity of the fabrication processes, green synthesis, utilizing eco-friendly reducing agents, is increasingly employed for sustainable fabrication in applications like sensing, catalysis, and photothermal therapy.^334^

For the abovementioned production difficulties and environmental concerns, recently, particular attention has been focused on the use of biodegradable photothermal materials (such as lignin^340^ and cuttlefish ink^341^) for the development of biodegradable actuators and soft robots. Lignin is a complex, amorphous biopolymer derived from plant cell walls, consisting primarily of phenolic subunits linked by ether and carbon-carbon bonds. It is a renewable and abundant byproduct of the pulp and paper industry, with methods of lignin extraction including alkaline and acidic processes. Photothermal conversion in lignin is attributed to its strong light absorption in the UV and visible ranges, facilitated by the delocalized electrons in its aromatic structure. The absorbed energy undergoes efficient nonradiative decay, yielding heat with a reported efficiency of η = 54%.^340^ Lignin also exhibits notable mechanical performance, with tensile strengths of 30–50 MPa and Young’s moduli ranging from 2–10 GPa, depending on its source and processing conditions.^342^ Lignin can be incorporated into composites by blending, molding, or in situ polymerization, where it contributes to enhanced mechanical strength. However, its complex and heterogeneous structure poses challenges in achieving uniform properties during blending and processing. Additionally, chemical modifications may be required to enhance its compatibility with other materials, which can complicate production.

Cuttlefish ink, composed of natural melanin nanoparticles, has gained increasing interest for its excellent dispersion stability, biodegradability, and remarkable ability to convert photothermal energy. As an eco-friendly substitute for traditional synthetic photothermal agents, cuttlefish ink nanoparticles (CINPs) have found applications in areas such as biomedicine, solar interface evaporation, thermal regulation, and actuation.^341^ In a 0.02% w/w dispersion, the cuttlefish ink nanofluid had a maximum overall PCE of 60.2% under solar simulator irradiation.^343^

Compared to the previously mentioned photochemical materials, photothermal materials can harness a broader portion of the solar spectrum and enable reversible actuation through temperature-mediated processes, which make them the main mechanism in sunlight-driven soft robots. For these reasons, the following discussion will focus exclusively on photothermal actuators and soft robots.

### 4.2 Material Selection Rationale

In Table 5, we summarize the PCE, working mechanism, and environmental compatibility of the most employed photothermal materials. An ideal material for photothermal actuators should possess a high light-to-heat PCE to enable rapid and substantial deformation. High-performance nanomaterials such as MXenes, CNTs, and plasmonic nanoparticles are excellent candidates, as they not only convert sunlight efficiently into heat but also provide high thermal conductivity that facilitates fast heat transfer to the thermo-responsive or hygroscopic components for actuation, while promoting rapid cooling once sunlight intensity decreases. Given that sunlight is a broad-spectrum source, broadband absorbers such as MXenes and CNTs are particularly advantageous for maximizing efficiency. Beyond thermal performance, the mechanical properties of photothermal materials are equally critical. They must be robust and resilient to support repeated heating–cooling cycles and reversible actuation without fatigue or structural failure. Since photothermal layers are typically combined with thermo-responsive or hygroscopic counterparts in composite systems to achieve ultimate deformation, compatibility between the two materials is essential for optimizing actuation behavior. For fabrication concerns, photothermal materials should also be easily processable (e.g., coating, blending) into composite architectures, such as reinforced films or laminates, to enhance mechanical robustness and functional adaptability. Finally, environmental considerations such as nontoxicity and biodegradability are increasingly important, with natural materials like lignin and bio-derived nanostructures such as CINPs offering sustainable alternatives for environmentally responsible applications.

**Table 5:** Photothermal Materials for Sunlight-Driven Soft Robot

**Material**	**PCE (@808 nm)**	**Working mechanism**	**Properties and environmental compatibility**
**MXene**	90%–100% ^335,336^	Nonradiative recombination of electron–hole pairs and LSPR effect	Broadband light absorption and high photothermal conversion efficiency. Partial enzymatic degradation by horseradish peroxidase + H_2_O_2_; toxic to aquatic organisms; toxicity reduces after degradation.^297^
**GO**	58%^337^	Nonradiative relaxation of delocalized π electrons	Highly dispersible in water and biocompatible. Easy functionalization. Partial biodegradation by peroxidases, including MPO;^287^ its toxicity is dose-, size-, and surface-chemistry-dependent.
**CNTs**	SWCNTs 60%	Nonradiative relaxation of delocalized π electrons	High thermal conductivity, high chemical stability, and low degradation. Partial biodegradation by peroxidases, including MPO.^344^ CNTs can have respiratory toxicity and cytotoxicity in cells.
	MWCNTs 68%^332^		
**Plasmonic NPs**	Au NPs: 48%–86%; Ag NPs: 72%; Fe NPs: 67%; Mn NPs: 70%^334^	LSPR effect	Tunable plasmon resonance, large absorption, facile synthesis in situ and ex situ. Not biodegradable. Some plasmonic NPs can be toxic, such as Ag NPs, Fe NPs, and Mn NPs.
**Lignin**	54%^340^	Non-radiative relaxation of delocalized π electrons in HOMO–LUMO levels	Low-cost, with biodegradability and renewability. Nontoxic.
**CINPs**	60.2%^343^	Absorption of NIR light and conversion into heat	Low-cost, with biodegradability and renewability. Good dispersibility in water. Generally nontoxic.

### 4.3 Sunlight-Driven Soft Actuators and Robots

In the following paragraphs, we will review the example of photothermal soft robots developed over the last 15 years. The scientific literature on actuators stimulated by light sources is extensive and typically focuses on narrowband light-emitting diodes (LEDs) or laser sources, mainly in the NIR range.^327,332,345–347^ Since this review aims to provide an overview of soft robots powered by sunlight, we focus on actuators and robots that operate under light sources (natural or artificial) within the solar spectrum and typically at optical irradiation levels near 1 sun (100 mW cm^–2^).

The fundamental building block of photothermal soft robots is the actuator, which converts optical power into mechanical work. In realistic conditions, sunlight provides a broad blackbody spectrum spanning from UV to IR.^348^ A widely adopted strategy for designing photothermal actuators is the bilayer structure, which couples an active material that undergoes thermally induced stress with a passive, nonresponsive layer. The structural asymmetry between these layers amplifies and enhances motion, making this design both simple and efficient.

MXenes have broadband photothermal absorption properties and multifunctionalities, making them highly promising candidates for photothermal actuators. Cai et al. reported a bilayer-structured actuator composed of MXene-cellulose composites (MXCC) paired with a polycarbonate (PC) membrane that could be powered by low-intensity NIR light.^349^ This actuator, fabricated through a solution-drying method, demonstrated remarkable multiresponsiveness to stimuli, including light, humidity, and electric fields, thanks to photothermal, hygroscopic behavior and electrical conductivity of MXenes.^350^ The basic principle of motion was related to water desorption due to the temperature increase of the photothermal material. Under an NIR lamp (650–1050 nm) with a power density of 80 mW cm^–2^, the material showed an increase of temperature *ΔT* = 50 °C in 7 s. Considering the composite actuator, it achieved a force of 45 mN under 100 mW cm^–2^ of NIR illumination. Notably, the study also showcased the actuator’s ability to operate using natural sunlight, including a crawling motion, highlighting its potential for fully sustainable applications. Besides low-intensity NIR light excitation, many MXene-based systems can be directly powered by sunlight. Zhao et al. developed a bimorph textile actuator that can be produced at scale using conventional textile manufacturing techniques and autonomously triggered by sunlight.^351^ The actuator’s active layer consisted of an MXene-modified PA filament, while the passive layer was made from polypropylene tape. The opposing thermal expansion characteristics of the two layers, combined with the outstanding PCE of MXene, enabled the actuator to exhibit effective deformation (1.38 cm^–1^) under sunlight exposure with a power density of 100 mW cm^–2^ (Figure 9a, panel I). This innovative design offered a promising pathway for wearable, sunlight-triggered actuators, particularly for applications in smart breathable textiles (Figure 9a, panel II). Similarly, Hu et al. presented an MXene-based bimorph actuator with an asymmetric and enlarged microstructure designed to harness natural sunlight for directional self-locomotion (Figure 9b, panel I).^352^ The actuator, referred to as the I-MXene/PE bimorph, was fabricated by adhering an adhesive PE film onto a freestanding I-MXene film, followed by cutting it into predetermined shapes. The I-MXene film, enhanced by intercalating 3-isocyanatopropyltriethoxysilane (IPTS) into MXene nanosheets, features increased interlayer spacing, an asymmetric microstructure, and improved mechanical properties. This structure allowed the actuator to achieve reversible bending deformation with a macroscopic amplitude and rapid response (3 mm in 0.6 s) to illumination. Simulated sunlight irradiation was provided by a xenon lamp equipped with the simulated sunlight filter. The interlayer spacing facilitates water molecule intercalation and volume changes, while the asymmetric microstructure amplifies microdeformations. When exposed to natural sunlight, the actuator delivers remarkable performance, achieving a 346° deformation in just 1 s (Figure 9b, panel II). Under intensified sunlight (200 mW cm^–2^), it achieves ultralarge deformations of approximately 700° in 2.1 s. This biomimetic design underscores the potential of MXene-based bimorphs for high-efficiency, sunlight-driven actuation in ambient environments, including a demonstration of locomotion driven by sunlight intensity fluctuation with a speed of 5.3 mm min^–1^ (Figure 9b, panel III). Another example of using MXenes for coupled photothermal and hygroscopic actuation was a nacre-inspired layered MXene/sodium alginate composite (MXSA), which exhibited excellent solar-level actuation performance.^353^ The hybrid structure absorbed sunlight across the UV–Vis–NIR range and converted it into heat, further promoting water desorption in sodium alginate for enhanced actuation performance. When an MXSA bending actuator was exposed to 1 sun irradiation, its curvature increased from 0.1 to 1.45 cm^–1^ in 14 s, and recovered in 155 s after the light was off. However, the curvature was not fully recoverable due to the irreversible water loss into the low RH environment. A light-triggered switch was demonstrated as the application of this electrically conductive bending actuator. Moreover, a wood-based composite was developed by Zhang et al. by coating MXenes onto porous delignified wood (TDW), which was combined with a low-density polyethylene (LDPE) layer to form a bending actuator.^354^ Under 120 mW cm^–2^ simulated sunlight, the TDW film exhibited excellent PCE of 70.4%, and the corresponding bending actuator showed about 100° angle change. The authors demonstrated a bionic flower made of bilayer actuators that could fold and bloom under natural sunlight excitation, a light-triggered gripper for cargo transportation, and a light-triggered circuit switch for adaptive curtain control.

**Figure 9:**
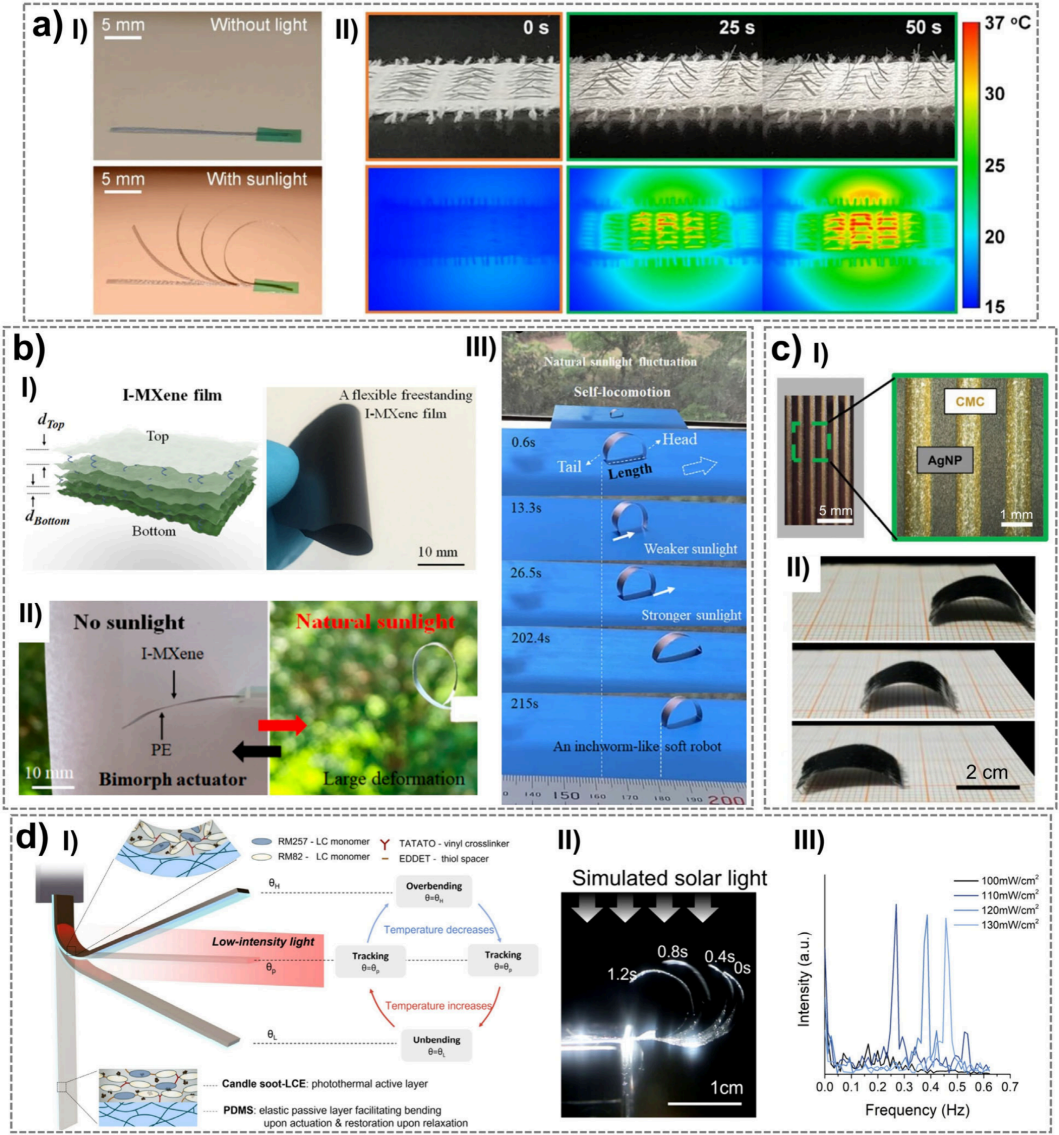
Simulated sunlight-driven soft actuators and robots. a) A sunlight-triggered bimorph textile actuator: I) optical images of a textile actuator driven by sunlight, with overlay of the time-lapse images; II) optical and infrared photos of the smart thermoregulation fabric under simulated sunlight (1 sun) irradiation. The cut piles open during human exercise to regulate skin temperature. Reprinted with permission from ref 351 Copyright 2021 American Chemical Society. b) MXene-based sunlight-driven soft robot: I) schematic diagram of the I-MXene film and an optical image of the bending a freestanding I-MXene film; II) optical images of reversible bending deformation of a bimorph actuator exposed to natural sunlight irradiation; III) optical images of the directional self-locomotion for the inchworm-like soft robot driven by natural sunlight fluctuation. Reprinted with permission from ref 352 Copyright 2021 American Chemical Society. c) Nanoplasmonic sunlight-driven soft robot: I) optimal microscope images of the DIW printed CMC paths on a PDMS covered with Ag NPs; II) crawling test of the rectangular actuator (2 × 1 cm) under 1 sun light exposure cycles. Reprinted with permission from ref 333 under CC BY 4.0, Copyright 2023 The Authors. d) Self-excited oscillator LiLBot under continuous light excitation: I) schematic showing the oscillation mechanism of the photothermal actuator under continuous light exposure; II) and III) superimposed sequential snapshots, angle changes, and frequencies of oscillation powered by a solar simulator. Reproduced from Zhao et al., Science Robotics, DOI: 10.1126/scirobotics.adf4753 [2023], AAAS.^355^

Carbon-based materials (CNTs, GO, carbon black) have also shown significant promise in the development of light-driven soft robots.^332^ Although most related demonstrations are excited by high-intensity laser sources to achieve substantial movement,^356,357^ several innovative systems show unique capabilities under sunlight irradiation. In 2014, a PC/SWCNT bilayer was among the first demonstrations of photothermal actuation directly under 1 sun (100 mW cm^–2^) by Zhang et al.^358^ The high broadband absorption and excellent heat transfer of SWCNTs enabled rapid bending of ∼ 90° within 0.67 s under light, and recovery in 0.87 s when the light was off. The authors demonstrated a smart curtain using an array of these actuators that could open and close with respect to the light intensity. For another example, Sun et al. developed a long-term self-propelled soft robot by assembling a solar-absorbing layer of carbon black particles on a hydroxyethyl poly­(2-hydroxyethyl methacrylate-*co*-acrylic acid) [p­(HEMA-*co*-AA)] hydrogel.^359^ The material’s combination of hydrophilic and hydrophobic groups facilitated self-propulsion on water surfaces by dynamically establishing asymmetric surface tension through wetting processes. The carbon black particles served as photothermal conversion materials, driving water evaporation inside the hydrogel under constant light irradiation. The evaporation efficiency was significantly enhanced by optimizing the polymer network structure and the carbon black layer’s thickness, enabling a dynamic balance between water absorption and evaporation for sustained locomotion. This self-propelled soft robot exhibited exceptional performance, including directionality through geometry design, rapid movement (5.19 cm s^–1^), and long-term operation (1760 min) under constant simulated sunlight equivalent to 1 sun. The system’s versatility extended to practical applications such as garbage collection, oil exploration, targeted goods transportation, and self-assembly. These advancements emphasized the potential of carbon-based materials for sustainable and multifunctional soft robotic systems.

Typical plasmonic NPs-based photothermal systems have narrowband absorption; thus, most robotic demonstrations are excited by narrowband light sources (e.g., NIR) with high intensity.^360,361^ This imposes a challenge on continuously producing useful and practical work using plasmonic NPs under low-intensity, broadband sunlight. Addressing this challenge, Mariani et al. introduced a plasmonic photothermal-hygroscopic actuator using Ag NPs on a PDMS substrate, integrated with a CMC track produced through DIW (Figure 9c, panel I).^333^ Ag NPs were synthesized in situ on a PDMS surface via a one-step fluoride-assisted synthesis, achieving 40% surface coverage. This design provided broadband absorbance in the visible spectrum (absorbance > 1) and a PCE of 40%. Using a mechanical model to predict curvature and forces, the actuator exhibited a response time of 6.8 s, a curvature change of 43%, and a maximum force of 0.76 mN under 1 sun (100 mW cm^–2^) simulated solar irradiation. These characteristics make it well-suited for various tasks, as lifting (energy density of 0.23 kJ m^–3^), crawling under modulated light on–off cycles (20 mm min^–1^, Figure 9c, panel II), rolling, and grasping. The authors also provided a demonstration of the actuator as a coating layer to functionalize passive materials, widening the possibilities of application in the field of soft robotics.

Most of the abovementioned photothermal robots change shape, produce force, or move based on the variation in the light intensity, which can be used as an adaptive strategy to natural light conditions. Utilizing steady-state light exposure to achieve continuous motion is a challenging task. The simplest design for addressing this challenge is a shape-changing roller. For example, in 2014, Zhang et al. demonstrated a roller made of bilayer PC/SWCNT can continuously roll away from the directional light coming from the left, with a speed of 6 cm s^–1^.^358^ Another solution is to use the out-of-equilibrium oscillatory behavior of photoresponsive materials to produce continuous oscillations under constant light exposure. In 2023, Zhao et al. presented a groundbreaking design for a fully autonomous soft robot (LiLBot), driven by sunlight through a self-excited oscillator (Figure 9d, panel I).^355^ This oscillator consisted of a candle soot-doped LCE/PDMS bilayer (CLP), created using DIW, and exhibited various autonomous behaviors. The system leveraged the low nematic–isotropic phase transition temperature of LCE, powered by temperature gradients induced by photothermal conversion. The CLP bilayer could generate chaotic oscillation under 100 mW cm^–2^ sunlight excitation, and constant frequency oscillation under 110 mW cm^–2^ excitation (Figure 9d, panels II and III). In addition, autonomous, continuous movement was achieved through oscillatory motion of stimuli-responsive materials, triggered by a constant, directional, and narrow-beam NIR light source (laser pointer, 808 nm) with higher intensity. This work demonstrated the potential of using a steady-state light source as the power source for powering the self-oscillation motion of photoresponsive actuators through the spatial light and thermal gradients. More recently, in 2024, Hou et al. combined the photothermal Au NPs and reduced GO (r-GO) in a thermo-responsive PNIPAM hydrogel, and developed an underwater phototactic robot that resembled a jellyfish.^362^ This phototactic vehicle self-regulated its motion by converting light into localized heat, generating asymmetric deformation and flows that propelled it toward the light source. It could be powered by ambient-level illumination, such as simulated sunlight at 60 mW cm^–2^, for underwater movement, while providing omnidirectional maneuverability through directional light excitation. This work highlighted how the photothermal effect, combined with a thermofluidic field, generates complex locomotion underwater.

In the context of sustainable material selection, lignin-based materials offer an eco-friendly solution as a photothermal component for smart elastomers. Cecchini et al. described the creation of a bilayer photothermal and biodegradable bending actuator, made from a PCL-lignin blend, 3D printed on a cellulose acetate substrate.^363^ When exposed to simulated solar irradiance of 300 mW cm^–2^, the actuator exhibited a 25.34% curvature change, a bending moment of approximately 80.2 μN m, and an actuation time of 30 s. Additionally, the photothermal blend achieved a PCE of 13.5%. Due to its photothermal and biodegradable qualities, this actuator could serve as a battery-free tool for various outdoor applications, especially in disposable applications. Moreover, using CINPs, Chen et al. reported a biomass-based multistimuli-responsive actuator based on wood-derived CNF and bioderived PLA.^364^ A patterned CINPs/CNF composite (CICC) film was successfully fabricated using mask-assisted vacuum filtration. It exhibited outstanding PCE and remarkable hygroscopic sensitivity. Under light exposure to the 0.2 W cm^–2^ NIR irradiation, the temperature on the CICC increased to 54 °C in 10 s, while under 0.6 W cm^–2^, the bilayer actuator generated bending deformation and bending curvature up to 2.28 cm^–1^ in 10 s. The actuator was also implemented in the development of soft robots for untethered grasping, weightlifting (using a mass 10 times the robot mass), and climbing. Although this robot was not powered by sunlight energy, it represented a good example of how biodegradable materials can still be used for the development of photothermal actuators/robots. In Table 6 we report a summary of the photothermal soft actuators and robots powered by simulated sunlight, similar to the sunlight spectrum or intensity. For the energetic sustainability classification, photothermal systems capable of actuation and continuous operation under natural sunlight intensities up to approximately 100 mW cm^–2^ (1 sun) are classified as fully sustainable. Systems that require moderately elevated irradiance levels exceeding 1 sun but remaining below the solar constant (136 mW cm^–2^) are considered partially sustainable, as such conditions may only be accessible in specific or transient environments. In contrast, photothermal systems that rely on highly concentrated illumination or artificially programmed light on–off cycles to achieve actuation are categorized as laboratory-level demonstrations.

**Table 6:** Simulated Sunlight-Powered Soft Actuators and Robots

**Soft actuators and robot**	**Materials and fabrication process**	**Performance**	**Energetic sustainability classification**	**Applications**	**Ref**
**I-MXene/PE bilayer robot**	Freestanding I-MXene film on an adhesive PE film.	Natural sunlight: deformation of 346° within 1 s. Intensified sunlight (200 mW cm^–2^): deformation of 700° in 2.1 s. Locomotion driven by natural sunlight fluctuation with a speed of 5.3 mm min^–1^.	Fully sustainable	Locomotion: crawling	^352^
	Fabrication: vacuum filtration.				
**Nacre-like composite bending actuator**	Layered Mxene/sodium alginate composite.	Under simulated sunlight (1 sun): curvature increased from 0.1 to 1.45 cm^–1^ in 14 s, and recovered in 155 s when the light was off.	Fully sustainable	Light-triggered switch	^353^
	Fabrication: codispersion and solvent casting.				
**Light-driven wood-based actuator**	Bilayer: a layer of TDW consisting of MXenes on delignified wood and a layer of LDPE.	Under 120 mW cm^–2^ simulated sunlight (1.2 sun): PCE of 70.4%, bending angle of 100°. Bending tested under 70–170 mW cm^–2^ simulated sunlight.	Fully sustainable for morphing, laboratory-level for gripping	Light-triggered morphing structures, gripper, and switch	^354^
	Fabrication: coating and densification, gluing.				
**Carbon-based floating swimmer**	Carbon black particles dispersed in a p(HEMA-*co*-AA) hydrogel.	Under continuous sunlight (1 sun): movement speed at 5.19 cm s^–1^ on water, and long-term operation (1760 min).	Fully sustainable	Locomotion: floating, swimming	^359^
	Fabrication: molding.				
**PC/SWCNT bilayer actuator**	Bilayer: SWCNTs on a PC membrane.	Under simulated sunlight (1 sun): rapid bending of 90° in 0.67 s under light, and recovery in 0.87 s when the light was off. Rolling under continuous, directional light: speed of 6 cm s^–1^.	Fully sustainable	Light-adaptive curtain, locomotion: rolling	^358^
	Fabrication: vacuum filtration.				
**Plasmonic bilayer actuator**	Bilayer: Ag NPs integrated on CMC tracks. Ag NPs were synthesized in situ on a PDMS substrate.	Under simulated sunlight (1 sun): energy density 0.23 kJ m^–3·^ Crawling motion of 20 mm min^–1·^	Laboratory-level	Graspers; locomotion: crawling and rolling	^333^
	Fabrication: one-step fluoride-assisted synthesis and DIW.				
**LiLBot**	Bilayer: candel soot-doped LCE on PDMS.	Under continuous simulated sunlight (1 sun): chaotic oscillation. Under continuous intensified sunlight (>1.1 Sun): constant frequency oscillation (∼0.25 Hz under 1.1 Sun).	Partially sustainable	Locomotion: swimming, walking, rolling.	^355^
	Fabrication: DIW.				
**Self-regulated underwater phototactic vehicle PTV**	Au NPs and r-GO in a PNIPAM hydrogel.	Under continuous simulated sunlight (0.6 sun): continuous movement toward the light. Under directional light: omnidirectional maneuverability under water.	Fully sustainable	Locomotion: swimming underwater	^362^
	Fabrication: molding.				

The autonomous sunlight-driven soft actuators and robots discussed leverage the photothermal effect as a promising step toward sustainable and untethered robotics. By converting solar energy into localized heating, these systems induce material deformation through thermal expansion, hygroscopic motion, surface tension changes, or thermal flows underwater, enabling controlled movement without the need for batteries or external power sources. So far, most demonstrated sunlight-driven robots are made of nanomaterials to achieve high-performance functionality, which are not sustainable for practical applications in nature. Improving the efficiency and performance of biodegradable photothermal materials is a future direction toward sustainable soft robotics. Moreover, most current photoresponsive systems adapt primarily to light being switched on or off, and reversible or continuous motions are typically powered by fluctuations in sunlight intensity. However, this reliance on naturally variable sunlight can limit actuation speed and predictability under outdoor conditions. Harnessing constant sunlight as a stable power source to drive reversible motion, therefore, represents an important research direction for advancing solar-actuated soft systems. This will require advanced robot structure designs to enable self-shallowing behaviors throughout an actuation cycle. At the same time, beyond using solar power for actuation, harvesting and storing solar energy through photovoltaic (PV) cells offers another route to sustainable operation, where collected energy can be stored as electricity and later used to power conventional motors and actuators. A brief discussion of solar energy storage is provided in the following subsection.

### 4.4 Solar Energy Harvesting

PV conversion remains the most widely adopted mechanism for harvesting solar energy, relying on semiconductor-based solar cells to convert photons into electricity. The generated electricity can be stored and subsequently used to power electronic devices, motors, or actuators, providing a direct energy supply for autonomous systems.^365^ In solar cells, light absorbed in the photoactive layer generates electron–hole pairs, which are transported to the cathode and anode through charge transport layers that promote efficient carrier mobility and suppress recombination.^366^

While crystalline silicon (c-Si) dominates the commercial PV market, its rigidity limits applicability in emerging fields such as flexible electronics^22,23^ and soft robotics. For soft robots, which demand compliance, lightweight, and adaptability to dynamic environments, alternative PV materials and architectures are more suitable. Candidate technologies include amorphous silicon (a-Si), copper indium gallium diselenide (CIGS), organic solar cells (OSCs), perovskite solar cells (PSCs), and dye-sensitized solar cells (DSSCs). These systems offer the potential for lightweight, flexible, and even semitransparent formats, expanding their usability in deformable or wearable robotic platforms.^367^ Flexibility can be further enhanced by the geometry design of the PV or solar cells. For example, fiber/fabric-type organic PV materials can withstand large deformation without failure, while wavy-form nonstretchable PV materials can also exhibit better stretchability than conventional solar cells.^368^ Coupling flexible/stretchable PV modules with compatible energy storage units, such as thin-film batteries or supercapacitors, can provide the cyclic release of energy required for soft robot actuation, whether by powering electronics directly or indirectly inducing deformation through the Joule effect.^366^

In this way, PV-based solar harvesting not only complements photothermal actuation strategies but also offers a pathway toward fully self-sufficient soft robotic systems. Nevertheless, important challenges remain, including the relatively low TRL of small-scale flexible or stretchable PV cells (currently around TRL 5–6), the complexity of seamlessly integrating PV modules, energy storage devices with soft robotic architectures, and the sustainability concerns associated with the nondegradable or toxic materials typically used in PV devices for environmentally oriented applications.

## 5 Soft Robots Powered by Osmosis

In this section, we examine soft materials, actuators, and robots powered by osmosis. Osmosis is attractive for soft robotics because it enables the conversion of chemical potential gradients into mechanical deformation through liquid water transport across semipermeable membranes, offering a potentially sustainable actuation mechanism. Here, we briefly introduce the physical basis of osmotic actuation, highlight biologically inspired design principles, and focus on material and structural strategies that enable osmotic soft robots to operate under environmentally realistic ionic-strength conditions.

In biological systems, osmotic pressure regulation exists in a wide range of nonmuscular plant movements, where changes in intracellular turgor pressure modulate tissue stiffness and drive macroscopic deformation.^369,370^ Plants function as distributed hydraulic systems in which spatially controlled osmolyte gradients produced through transmembrane ion-pumping proteins can generate reversible mechanical responses.^371^ In many multicellular plant movements, such as those triggered by touch, light, or thermal stimuli, osmotic water transport and turgor pressure variation execute deformation, while the initiating stimulus and energy supply are internally regulated. In contrast, certain systems, most notably stomatal opening and closure (Figure 10), are more directly coupled to environmental conditions, where changes in external humidity, CO_2_ concentration, or ionic environment modulate osmolyte gradients and drive osmotic actuation without centralized control.^370^ These latter examples provide particularly relevant bioinspiration for osmotic soft robotic systems that aim to harness naturally occurring osmotic gradients as an environmental energy source.

**Figure 10:**
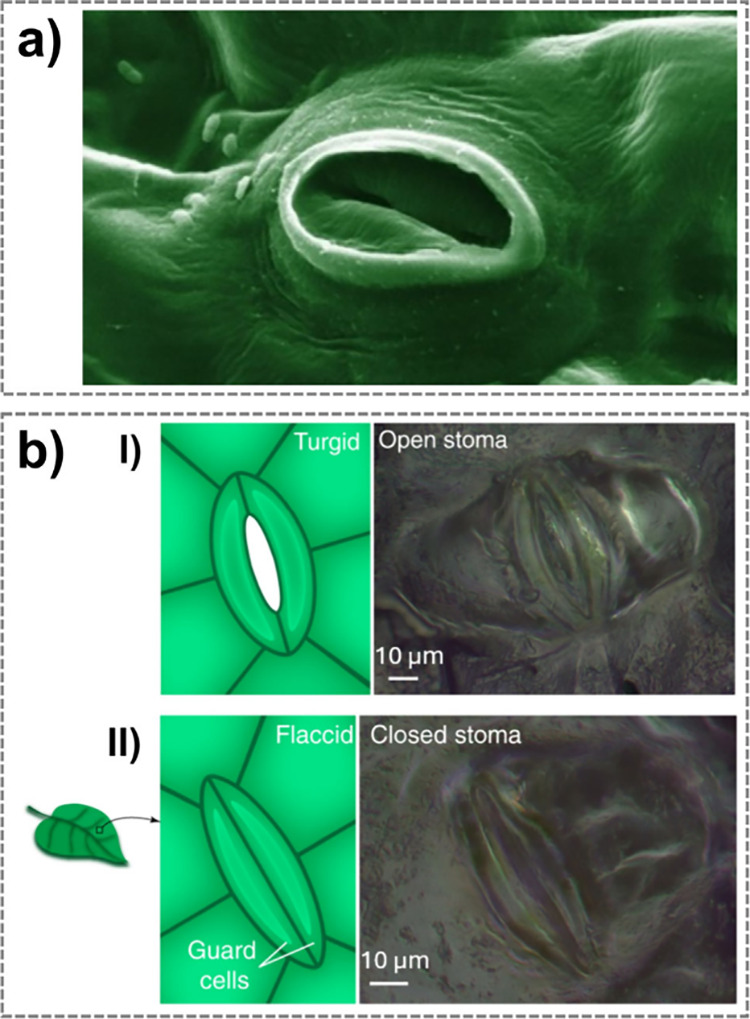
a) Example of osmosis-based movements in plants: Stomata guard cells. Reprinted with permission from ref 370 under CC BY 4.0, Copyright 2014 Public Library of Science. b) Osmosis-based reversible actuation of stomata guard cells in I) flaccid and II) turgid state: schematic and optical images of *Tradescantia zebrina* leaves. Reprinted with permission from ref 371 under CC BY 4.0, Copyright 2019 The Author(s).

At the material level, osmotic pressure is governed by the concentration gradient of dissolved species, with the resulting solvent flux and mechanical response determined by membrane permeability and osmolyte mobility. For soft robotic applications, the magnitude and reversibility of osmotic actuation are therefore tightly coupled with the ionic strength of the surrounding environment. Fully sustainable ionic strengths generally occur within freshwater-to-seawater salinity regimes (up to approximately 0.6 M), which are widely encountered in soils, estuarine and coastal regions, river–sea interfaces, and physiological compartments. Beyond this range, higher ionic strengths (≈ 0.6–1 M) can still occur in specific contexts, such as saline soils, alkaline or polluted waters, and certain wastewater streams. In these settings, spatial gradients (e.g., stratification, diffusion fronts) and temporal variations (e.g., rainfall, tides) naturally provide the driving force for osmotic deformation.

Accordingly, this section reviews common materials and actuator designs for osmotic soft robots, with emphasis on their chemical and mechanical properties and fabrication strategies that enable integration under environmentally realistic ionic-strength conditions.

### 5.1 Soft Materials for Osmotic Actuators

The osmotic materials most commonly employed in soft actuators and robotic systems are semipermeable polymeric hydrogels and membranes, including poly­(2-acrylamido-2-methylpropane sulfonic acid) (PAMPS), PAAm hydrogels, gelatin methacrylate (GelMA), poly­(vinyl alcohol) (PVA), poly­(3-sulfopropyl acrylate) potassium salt (PSPA), and polysulfone (PSF).

PAMPS hydrogel is the first material to be historically employed for the development of a soft actuator.^372^ It is a strong anionic and sulfonic polyelectrolyte, and it finds applications in ion exchange resins, water purification, hygienic products, and electrosensitive soft materials and robots.^373^ The monomer 2-Acrylamido-2-methylpropanesulfonic acid (AMPS) has a solubility of 500 g/L in water,^374^ and when UV-polymerized from an aqueous solution containing 60 wt% AMPS, and in the absence of any chemical cross-linker, it is able to absorb up to 1000 times its own weight in water without dissolving.^373^

PAAm hydrogel is another hydrogel frequently utilized for osmotic actuators. PAAm is synthesized via the chain-growth polymerization of acrylamide (AAm) monomers. During hydrogel preparation, AAm is copolymerized with a cross-linking agent, typically *N,N*’-methylenebisacrylamide (MBA), resulting in a 3D network of covalently cross-linked PAAm. The mechanical properties can be tuned to produce hydrogels in a wide range of stiffness (elastic moduli ≈ 0.1–340 kPa) and with a swelling ratio tunable according to the ratio of AAm and MBA in the range 1–46.^375^

GelMA hydrogel is a gelatin-based material that primarily contains methacrylamide groups along with some methacrylate groups, extensively utilized for fabricating 3D tissue-engineered constructs, but also osmotic actuators. When exposed to UV light in the presence of a photoinitiator, it undergoes radical polymerization, resulting in the formation of covalently cross-linked hydrogels.^376^ The Young’s modulus ranges from 0.1 to 265 kPa depending on gelatin type and concentration, the buffer system, the methacrylate (MA) concentration, and the degree of substitution (DS%), while the swelling ratio in PBS is 44.57 (5% w/v GelMA) or 4.05 (30% w/v GelMA).^377^

PVA hydrogel, whose chemical and physical properties are described in Section 3, can also be used to fabricate porous semipermeable membranes for osmotic actuators. The PVA hydrogel membrane, with a thickness of approximately 750 μm and pore diameters around 200 μm, can withstand a true stress of at least 1.2 MPa over 10,000 cycles of tensile loading.^378^

PSPA is a water-soluble polyelectrolyte polymeric hydrogel derived from 3-sulfopropylacrylate monomers. The monomer contains a sulfonate group (-SO_3_^–^) attached to a 3-carbon spacer, with the counterion being potassium (K^+^). The hydrogel shows a degree of swelling up to 7.5, but studies in the literature have shown that the mechanical properties and swelling of hydrogels can be controlled using different types of cross-linking molecules and by controlling the cross-linking density.^379^

PSF porous membranes are based on the amorphous high-temperature thermoplastic PSF, which has outstanding thermal stability (*T*_g_ ≈ 185 °C), strong mechanical performance (Young’s modulus ≈ 2.5 GPa), and resistance to chemicals (acid or base).^380^ Because of these properties, PSF ultrafiltration membranes are frequently used as the foundational layer in the production of composite membranes, which find applications across various fields such as reverse osmosis, chemical processing, water purification, and osmotic actuation. This widespread use is largely due to PSF’s industrial availability, cost efficiency, straightforward manufacturing process, resistance to thermal, chemical, and microbial degradation, and its capacity to function effectively over a wide pH range.^381–383^

### 5.2 Material Selection Rationale

Materials for osmotic actuators must exhibit strong osmotic responsiveness to enable substantial and reversible volume changes when exposed to solute concentration gradients. High hydrophilicity and solvent uptake are essential to generate sufficient actuation forces, while mechanical properties must balance flexibility and strength to permit repeated swelling-deswelling deformation without rupture under osmotic stress. Hydrogels and semipermeable polyelectrolyte polymers are particularly promising, as they can remain stable under typical osmotic gradients while providing large deformation.

Beyond actuation performance, environmental sustainability is another key consideration. Materials should be nontoxic and preferably biodegradable to minimize ecological impact in outdoor environmental applications, if not, device retrieval is performed. In this regard, polymers such as PVA^259^ and GelMA^384^ have shown promise as biodegradable hydrogels, with established use in biomedical contexts. Equally important is the selection of osmolytes incorporated within or used in conjunction with hydrogels. The trapped solutes (e.g., counterions in polyelectrolytes) drive swelling by establishing osmotic gradients, while external solutes control actuation through concentration changes. For environmentally sensitive applications, biocompatible and readily degradable osmolytes such as organic acids, salts with low ecotoxicity (like NaCl and KCl), or naturally derived small molecules are preferred over persistent or toxic species. Developing hydrogel-osmolyte systems that couple high actuation performance with environmental safety represents a critical step toward sustainable osmotic actuator design.

### 5.3 Soft Actuators and Robots Powered by Osmosis

Several examples of reconfigurators, actuators, and grippers driven by osmosis have been reported in scientific literature. Given the ability of hydrogels to swell through osmotic pressure, most of the osmotic-driven actuators and robots are based on hydrogels immersed in water or aqueous solvents with osmolytes.^385–387^ Although extensive efforts have been devoted to developing osmotic and osmosis-responsive hydrogel materials and actuators, their translation into fully functional soft robotic systems remains limited. This gap arises not from the material-level limitations but from intrinsic challenges associated with osmotic actuation, particularly the difficulty of achieving rapid and reversible deformation. While osmotic swelling can generate large strains, the recovery or shrinking process is often slow, incomplete, or dependent on external conditions, which complicates cyclic operation and control. As a result, despite the high potential of osmosis-driven actuation, relatively few soft robotic demonstrations have been realized to date.^388^ In the following paragraphs, we will review innovative soft actuators and milestones, as well as soft robots that have represented significant advancements in terms of approach, design, material development, and fabrication technology.

The earliest claims of osmotic actuators emerged in the context of micropumps for biomedical engineering applications. In 1983, the development of an electro-osmotic cell for actuating an implantable insulin micropump was reported.^389^ This was followed in 1992 by the introduction of an osmotic microcapsule pump.^390^ Notably, 1992 also marked a pivotal milestone in the field: the demonstration of the first soft hydrogel material capable of osmotically driven actuation coupled to electrokinetics.^372^ In this work, the authors described an electrically driven artificial muscle fabricated from a water-swellable PAMPS hydrogel (dimensions: 1 mm thickness, 5 mm width, 20 mm length). The actuator functioned through an electrokinetic molecular assembly reaction of surfactant molecules on the hydrogel surface when immersed in water. When the actuator was immersed in a dilute solution of n-dodecyl pyridinium chloride (C_12_PyCl) containing sodium sulfate (3 × 10^–2^ M), it swelled 45 times compared to the dry weight, with a consequent stretching of the structure. By applying a 20 V DC voltage using a pair of planar carbon electrodes, the actuator could bend reversibly, with a cycle period of 10 s. The actuator could also walk as a function of the applied current, salt concentration of surfactants, and sodium sulfate, reaching a maximum velocity of 25 cm min^–1^. Although this was not a purely osmotic actuator, the hydrogel material exhibited osmotic swelling, while its reversible bending and locomotion were governed by electrokinetic surface phenomena under an applied electric field, thus highlighting the interplay between osmotic and electrokinetic mechanisms in soft actuation.

Similarly to the previously reported proof of concept, in 2014, Morales et al. reported a millimeter-scale gel walkers that undergo directional motion in response to electric fields in solution.^391^ This motion was realized using gels with opposite charges fixed onto the backbone of the polymers that comprise the gels. The anionic and cationic legs were composed of AAm/sodium acrylate (NaAc) copolymer and AAm/quaternized dimethylaminoethyl methacrylate (DMAEMA-Q) copolymer, respectively, and fabricated through laser writing. Gel walker experiments were conducted on a PDMS substrate and after immersion in 0.01 M, 0.05 M, and 0.1 M NaCl solutions, controlling the direction of the electric field (5 V cm^–1^). The highest propulsion velocity achieved was ∼ 2.5 mm min^–1^. Although this case was not a case of classical osmosis, polyelectrolyte-doped gels immersed in ionic solutions exhibited electrically induced osmotic swelling, where the applied electric field redistributed mobile ions to create osmotic pressure gradients, thereby driving swelling and deformation of the gel.

For fully salinity-responsive osmotic actuators, significant progress has been made at the hydrogel material level and the simplest bilayer bending actuators and volume-expansion actuators. Polymer networks with tailored ionic chemistries have been shown to undergo large and reversible volume changes in response to changes in ambient salt concentration, which results in mechanical deformation and force output. For example, Xiao et al. developed bilayer hydrogels made of a polycationic layer and a polyzwitterionic layer, which were capable of reversible, bidirectional bending from a bending angle of – 39° in water, to + 150° in 1 M NaCl solution.^392^ Later, this group further synthesized PNIPAM/poly­(3-(1-(4-vinylbenzyl)-1H-imidazol-3-ium-3-yl)­propane-1-sulfonate) (PVBIPS) bilayer hydrogels with improved salt-responsiveness, which could achieve a bending angle change from – 310° (water) to + 340° (1 M NaCl), with a speed of 1.1°–6.5°/s (at NIPAM:VBIPS ratio of 3:7).^393^ A salinity-adaptive gripper and a switch were demonstrated as the potential use of the osmotic bending actuators. More recently, research has been carried out on more sensitive salinity-responsive actuators, such as a hydrogel made of copolymerized polyelectrolyte and zwitterionic polymer that could reversibly swell in water and shrink in low ionic strength solutions (0.01 wt% NaCl in water).^394^ The resulting hydrogels were shown as intelligent liquid valves with sensing and actuating functions to regulate water quality.

The approaches reported so far refer mainly to casting and molding techniques able to do bending or basic expansion. For the development of more complex actuators, movements, and soft robots, 3D printing techniques could be used. Odent et al. developed a highly stretchable (up to 425%), tough (up to 53.5 kJ m^–3^), and resilient (up to 97% strain energy recovered at 100% strain) ionically conductive hydrogel that could be 3D printed using stereolithography (SLA) using riboflavin-triethanolamine photochemistry in water solution.^395^ The hydrogel was based on ammonium-containing PAAm and surface-modified sulfonated silica nanoparticles with a tensile modulus of 114 kPa and a SR of ≈ 4.2 after 2600 s. They also demonstrated an osmosis-driven gripper that could change from flat to closed within 10 min in dyed blue water and could recover when placed in ethanol. More recently, in 2024, Darkes-Burkey and Shepherd designed and developed the first 3D osmotically driven soft robot realized through computed axial lithography (CAL) (Figure 11a, panel I): an emerging single-step technology for manufacturing 3D sections, bypassing the traditional layered approach using tomography.^396^ In the work, they used a basic GelMA hydrogel osmotic actuator with an embedded endoskeletal system. GelMA was ideal for CAL because it was swellable and had reversible thermal gelation, enabling suspension of the endoskeleton during printing. Disks with 10 wt% of GelMA had the most swelling with 99.4% increase by mass after 7.5 h. The authors achieved a swelling-induced bending actuation of 60° after tuning the material formulation, design, and postprocessing, after 7 h of immersion in deionized water at 90 °C (Figure 11a, panel II). Although the time scale of the actuation was limited, this research represents a step forward in the development of 3D hydrogel-based soft robots driven by osmosis. Moreover, 2PP could be employed for the development of a 4D osmotic-driven microactuator at microscopic level. Ennis et al. developed a novel photoresist for the generation of fructose-responsive hydrogel microstructures based on a phenyl-boronic acid copolymer.^397^ Several microstructures (e.g., beams, flower-like structures) were realized with a height increase up to 234.5%, achieved using 5 mM of fructose in water.

**Figure 11:**
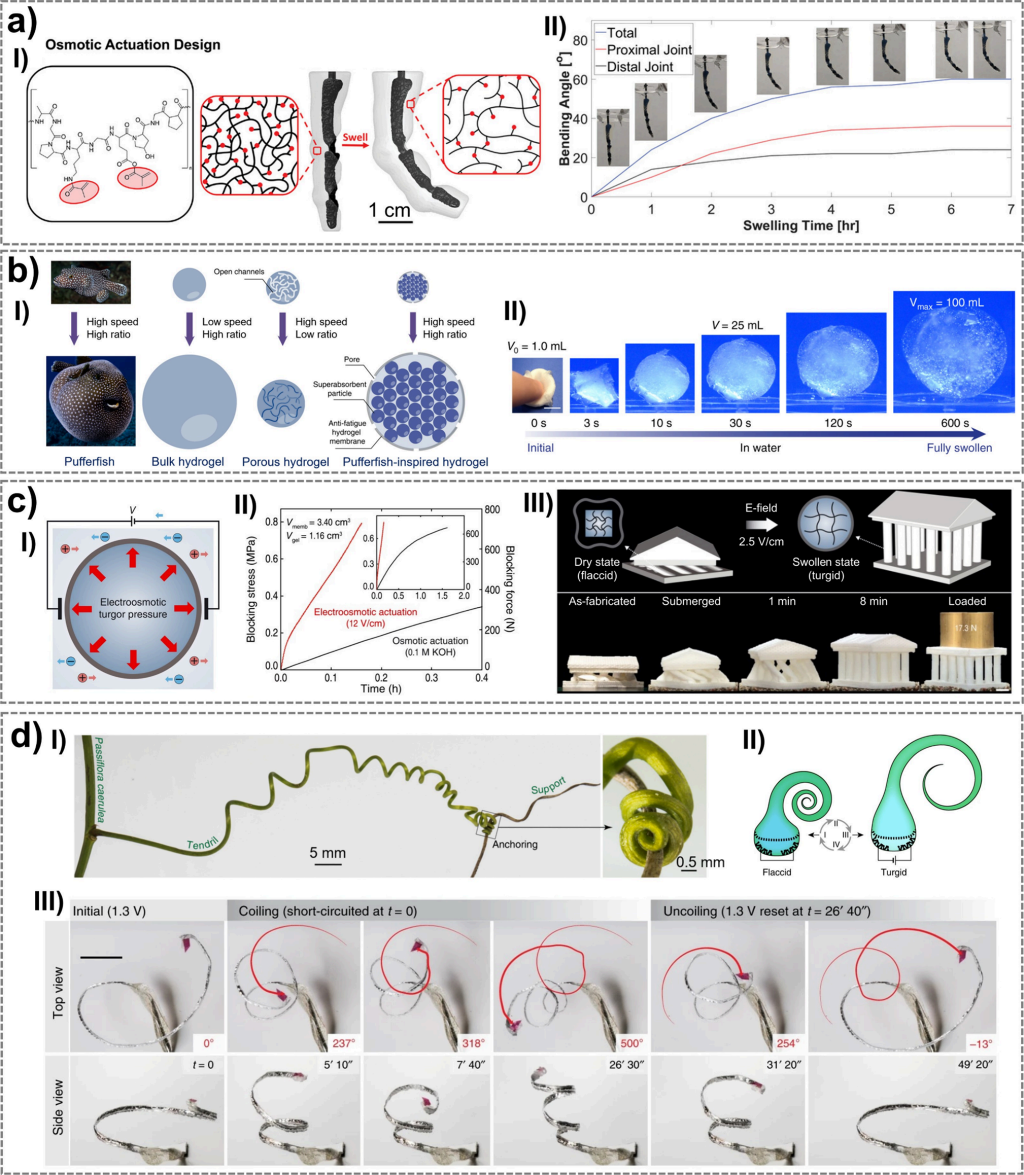
Osmotic-driven soft actuators and robots. a) GelMA hydrogel actuator 3D printed with CAL: I) GelMA monomer backbone with red circles indicating the MA groups that create chemical cross-links during UV radiation in the presence of a photoinitiator; II) timeline of the bending progression over 7 h. Reprinted with permission from ref 396 under CC BY 4.0, Copyright 2024 The Author(s). b) Pufferfish-like osmotic actuator: I) design of the pufferfish-inspired ingestible hydrogel device, which swells in water with both a high speed and a high ratio, II) time-lapse images of the hydrogel device swelling in water. Reprinted with permission from ref 378 under CC BY 4.0, Copyright 2019 The Author(s). c) Electroosmotic hydrogel turgor actuator: I) schematic illustration of the electroosmotic actuation process of a hydrogel turgor actuator; II) blocking stress versus time curves for the hydrogel turgor actuator actuated by osmosis and electroosmosis at zero stroke; III) rapid construction of the temple at 2.5 V cm^–1^ in 0.1 M KOH aqueous solution, and the completed temple endured two weights of 17.3 N force. Scale bar, 2 cm. Reproduced from Na et al., Science, DOI: 10.1126/science.abm7862 [2022], AAAS.^388^ d) Tendril-like soft robot: I) *Passiflora caerulea* tendril reaching and anchoring to an external support; II) artificial tendril actuation (schematic); III) tip rotation angle at selected times: the soft robot reversibly spanned ∼ 500° over ∼ 50 min. Reprinted with permission from ref 371 under CC BY 4.0, Copyright 2019 The Author(s).

In addition to the use of bulk hydrogels, hydrogel particles could also be employed for the development of osmosis-driven actuators. For example, Liu et al. developed a pufferfish-inspired hydrogel device, made of superabsorbent hydrogel particles of PAA, ∼ 450 μm in diameter, encapsulated in an antifatigue porous hydrogel membrane of PVA ∼ 750 μm in thickness and ∼ 200 μm in pore diameter (Figure 11b, panel I).^378^ The device could be ingested as a standard-sized pill (diameter of 1–1.5 cm), imbibed with water, and inflated (up to 100 times in volume within 10 min) into a large soft sphere (diameter of up to 6 cm, modulus of 3 kPa, Figure 11b, panel II). The device could maintain robustness under repeated mechanical loads (more than 26,000 cycles of 20 N force) over 2 weeks in vitro. In the future, it has the potential to be used as a material platform for soft medical devices or robots that closely interact with the digestive system in the human body. Besides medical applications, hydrogel particles can also be encapsulated into a piston structure to generate force output through osmotic pressure. Arens et al. developed an osmotic engine based on PAA hydrogel particles that could translate osmotic pressure (between water and 4.3 wt% NaCl seawater-like saline solution) into macroscopic mechanical force, achieving a maximum mean power of 0.23 W kg^–1^ dry hydrogel under an external load of 6 kPa.^398^ This device demonstrated fully sustainable osmotic actuation if the freshwater–saltwater chemical potential gradient is continuously regenerated by the surrounding environment (e.g., estuarine, tidal, or river–sea interfaces).

In general, stimuli-responsive hydrogel actuators suffer from weak actuation force and/or slow response speed. Despite substantial improvements in speed, actuation forces are, in general, limited because hydrogels are intrinsically soft.^388^ To improve the efficiency of purely osmotic actuation, electroosmosis can be applied by introducing an electric field. Electroosmosis refers to the steady and rapid movement of water through an electric double layer (EDL) present in charged porous material when subjected to an electric field. In polyelectrolyte hydrogels, fixed charges within the polymer network form this layer: counterions migrate through the polymer mesh, dragging water molecules that are then captured by the hydrophilic chains (Figure 11c, panel I). This active transport enables the hydrogel to swell much faster than through osmosis alone.^388^ The rate of electroosmotic flow (EOF) in a porous medium is influenced by the permittivity of the liquid, the electric field, the zeta potential of the medium, the porosity of the medium, the viscosity of the liquid, and the inlet area. For this purpose, Na et al. reported the development of a hydrogel-based actuator with a design that used turgor pressure and electroosmosis, which could achieve much higher actuation force (increased by a factor of 10^2^ to 10^6^, until 917 N) in a shorter time (9 min) than its purely osmotic counterparts (output similar force in 96 min, Figure 11c, panel II).^388^ The hydrogel was based on PSPA polyelectrolyte hydrogel and wrapped in a selective semipermeable membrane made of rayon and polyester blend. When immersed in 0.1 M KOH electrolyte and with an electric field ranging from 1 to 12 V cm^–1^, the hydrogel converted its inherent high osmotic pressure to a large actuation stress. The authors demonstrated the rapid underwater construction of 3D hydrogel shapes (Figure 11c, panel III) through electroosmosis at 2.5 V cm^–1^ in 0.1 M KOH aqueous solution.

In addition to hydrogels, semipermeable membranes have also been used for the development of soft actuators and robots. Starting from an osmotic actuation model,^399^ in 2014, Sinibaldi et al. reported a plant-inspired actuator (size ∼ 1 cm) able to produce ∼ 20 N force in ∼ 2 min using a concentration of NaCl as osmolyte (2 M).^370^ Differently from the previously reported examples made in hydrogel in this case the actuator was composed by: a structure in stainless steel; a reservoir chamber in plexiglass; an actuation chamber; a semipermeable forward-osmosis commercial membrane specifically designed for operation with NaCl with water permeability α_OM_ = 3·10^–13^ m s^–1^ Pa^–1^, rejection coefficient σ in the range 0.95–0.97, and very low performance degradation due to NaCl fouling elastomeric bulging disk. This study paved the way for the development of a tendril-like soft robot with variable stiffness bioinspired by *Passiflora caerulea* (Figure 11d).^371^ In the tendril-like soft robot, the osmotic membrane section of the artificial tendril was based on a porous PSF hollow fiber with 0.5 mm diameter, rated at 50 kD or 0.05 μm pore size, and PA film deposited on the inner wall. Sodium sulfate (Na_2_SO_4_) was chosen as an electrolyte at a low (0.1 M) concentration. The reverse osmotic actuation strategy was based on the electrosorption of ions on flexible porous carbon electrodes driven at low input voltages (1.3 V). The actuation consisted of ∼ 500° reversible rotation over 50 min and a 5-fold change of stiffness. The robot was conceived for soft robotics with biocompatible materials and safe voltages, based on electrochemically controlled osmosis. These examples adopted a hybrid-driven strategy in which osmosis provided the primary actuation force, while an externally applied electric field was used to regenerate the osmotic gradient, enabling continuous operation even in environments with fixed ionic strength. Although the electric field was supplied externally in current demonstrations, it could be generated from sustainable sources such as ambient energy harvesters, pointing to a viable pathway toward fully renewable hybrid osmotic systems.

In Table 7 we report a summary of osmosis-driven soft actuators and robots, with their functional materials, fabrication process, performance, and energetic sustainability classification. For this classification, hybrid-driven systems, where osmotic actuation is coupled with externally supplied energy for gradient regeneration, are identified separately. Pure osmotic systems capable of reversible operation under naturally occurring environmental ionic strengths up to approximately 0.6 M (e.g., freshwater to seawater conditions) are classified as fully sustainable, whereas those requiring higher but still environmentally plausible ionic strengths (0.6–1 M) are considered partially sustainable. In contrast, osmotic systems exhibiting only one-directional actuation or requiring non-naturally occurring ionic strengths or externally imposed solution cycling for reversibility are categorized as laboratory-level demonstrations.

**Table 7:** Soft Actuators and Robots Driven by Osmosis.

**Soft actuators and robots**	**Fabrication process and materials**	**Performance**	**Energetic sustainability classification**	**Applications**	**Ref**
**Multiarmed gripper**	Ammonium-containing PAAm, surface-modified sulfonated silica NPs, and riboflavin-triethanolamine photochemistry.	Stretchable up to 425%, toughness up to 53.5 kJ m^–3^, and resilience up to 97% strain energy recovered at 100% strain. Tested in deionized water: SR ≈ 4.2 after 2600 s.	Laboratory-level	Gripper	^395^
	Fabrication: 3D printing (SLA).	Osmosis-driven gripper: changing from flat to closed within 10 min in water, recovering in ethanol.			
**Salt-responsive hydrogel actuator**	PNIPAM/PVBIPS bilayer.	Bidirectional bending of the bilayer hydrogel. At NIPAM:VBIPS ratio of 3:7, bending angle changed from – 310° (water) to + 340° (1 M NaCl), with a speed of 1.1°–6.5°/s.	Partially sustainable	Gripper, adaptive switch	^393^
	Fabrication: molding, sequential radical polymerization.				
**Finger actuator**	GelMA hydrogel.	Tested in 90 °C deionized water: bending actuation of 60° angle change in 7 h.	Laboratory level	Endoskeleton systems in soft actuators	^396^
	Fabrication: 3D printing (CAL).				
**Pufferfish-inspired hydrogel device**	PAA superabsorbent hydrogel particles, encapsulated in an antifatigue porous hydrogel membrane of PVA.	Tested in deionized water: swelling volume ∼ 100 times, modulus of 3 kPa. Fatigue: over 26000 cycles of 20 N force over 2 weeks in vitro.	Laboratory level	Versatile platform for soft medical devices	^378^
	Fabrication: laser cutting of the membrane, gluing.				
**Osmotic engine**	PAA superabsorbent hydrogel particles.	Tested cyclic swelling in desalinated water and shrinking in seawater-like saline solutions (4.3 wt% NaCl).	Fully sustainable	Force actuator or engine	^398^
	Fabrication: free-radical polymerization for hydrogel synthesis, assembling for engine setup.	Maximum mean power: 0.23 W kg^–1^ dry hydrogel with an external load of 6 kPa.			
**Turgor actuator**	PSPA polyelectrolyte hydrogel, semipermeable membrane made of rayon and polyester blend. Osmolyte: KOH.	Purely osmotic: maximum actuation stress of 0.73 MPa in 96 min.	Hybrid-driven	Force actuator, underwater construction	^388^
	Fabrication: molding, laser cutting of the membrane, and gluing.	Electroosmotic: maximum actuation stress of 0.79 MPa in 9 min (electric field: 12 V cm^–1^).			
**Tendril-like soft robot**	Porous PSF hollow fiber membranes + PA film.	Actuation: ∼ 500° reversible rotation over 50 min and a 5-fold change of stiffness (requiring 1.3 V voltage for reversible motion).	Hybrid-driven	Bioinspired soft robotics	^371^
	Fabrication: synthesis and mechanical assembly.	Osmolyte: Na_2_SO_4_.			

In summary, osmotic actuators and robots can be broadly classified into two categories based on how the osmotic gradient is utilized and regenerated. Pure osmotic systems rely on passive swelling or deswelling driven by environmental chemical potential differences. While they often exhibit large deformation, their reversibility and cyclic operation are typically limited or not explicitly addressed at the system level. In contrast, electroosmotic systems employ externally applied electric fields to actively regenerate osmotic gradients, enabling faster and more controllable bidirectional motion, but they are hybrid-driven rather than fully environment-powered. Nevertheless, osmotic systems inherently possess the potential for adaptive deformation in response to environmental changes such as variations in humidity and salinity, which are features to be leveraged for passive sensing–actuation coupling and adaptive functions. In the future, hybrid strategies, including the integration of osmotic swelling with magnetically guided deformation,^400^ or electroosmotic actuation powered by solar energy harvesting (discussed in Section 4.4) or triboelectric energy harvesting (discussed in Section 7), offer promising pathways to gap between material-level osmotic actuation and practical, self-sustaining soft robotic platforms.

## 6 Soft Robots Powered by pH

In this section, we examine soft materials and robots driven by pH variations. We first analyze the environmental pH and physiological pH variations and the mechanisms by which nature can adapt in response to changes in acidity or alkalinity. We then explore stimulus-responsive materials that adapt to pH variations, concluding with a comparative analysis of soft robots actuated by pH-responsive materials.

pH is an indicator of the molar concentration of hydrogen ions, reflecting the balance between acidity and alkalinity on a logarithmic scale, defined as pH = – log_10_[a­(H^+^)].^401^ The scale ranges from 0 (highly acidic) to 14 (highly alkaline), with 7 as neutral. Environmental pH in natural water systems typically ranges from mildly acidic to weakly alkaline values, spanning from approximately pH 5 in clean rainwater to around pH 8 in oceans, as illustrated in Figure 12. This range can broaden substantially due to both natural and anthropogenic processes, including CO_2_ dissolution, acid rain (pH ≈ 4), carbonate mineral runoff, eutrophication, and agricultural or industrial discharges, which can locally raise pH to values approaching 9–10. Environmental pH strongly influences chemical equilibria and biological processes; for example, most freshwater fish thrive within a pH range of 6.5–9.0, while optimal nutrient uptake for many plants occurs at pH 5.5–6.5.^402^ Accordingly, pH values between approximately 4 and 10 can be considered environmentally accessible in natural or outdoor settings. In contrast, physiological pH in the human body is far more tightly regulated and spatially compartmentalized, spanning from strongly acidic gastric conditions (pH ≈ 2) to near-neutral or mildly alkaline environments in intestinal and extracellular fluids (pH ≈ 6–8). These context-dependent pH ranges define the realistic boundaries of pH variation encountered in environmental versus physiological systems.

**Figure 12:**
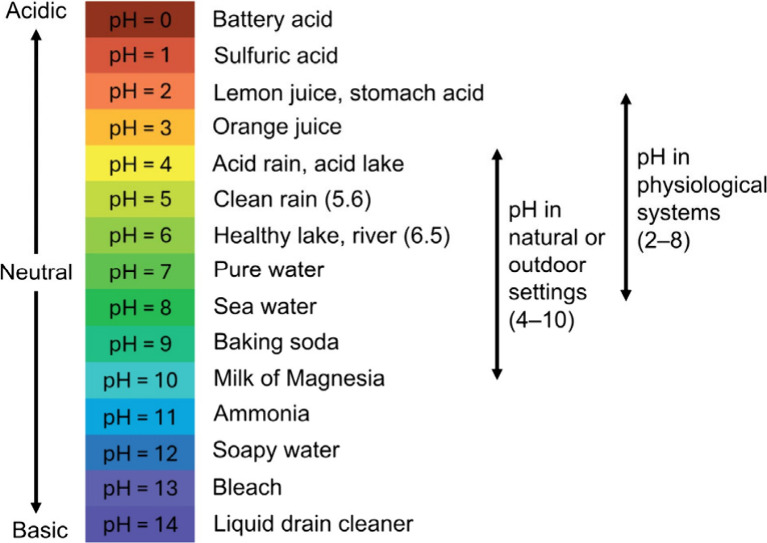
pH scale in environmental and physiological systems. Readjusted from ref. 405

While biological systems sense and respond to pH changes through complex biochemical pathways, synthetic systems can exploit pH variations more directly through material-level physicochemical responses. The following section reviews representative soft materials, actuators, and robotic systems whose operation is enabled by pH-responsive swelling and shrinking, commonly achieved using hydrogels that reversibly alter their volume in response to local pH conditions.^403,404^ Within this context, the relevant operational pH ranges naturally differ by application: outdoor or environmental pH-adaptive systems typically function within approximately pH 4–10, whereas biomedical and physiological applications are constrained to narrower pH windows, generally spanning pH 2–8.

### 6.1 pH-Responsive Materials

Actuators and robots capable of responding to pH are primarily based on polymers (mostly hydrogels) that can undergo shrinking and swelling with pH variations. Polymers that respond to pH are constructed using ionizable or acid-cleavable monomers within the polymer structures. Ionizable polymers generally consist of weak acids and/or bases that can be ionized by adjusting the solution’s pH, leading to the formation of polyanions, polycations, or polyzwitterions (Figure 13a, panel I). Conversely, polymers containing acid-sensitive groups can undergo cleavage triggered by acids or bases, resulting in the generation of a charged state (Figure 13a, panel II).^406,407^

**Figure 13:**
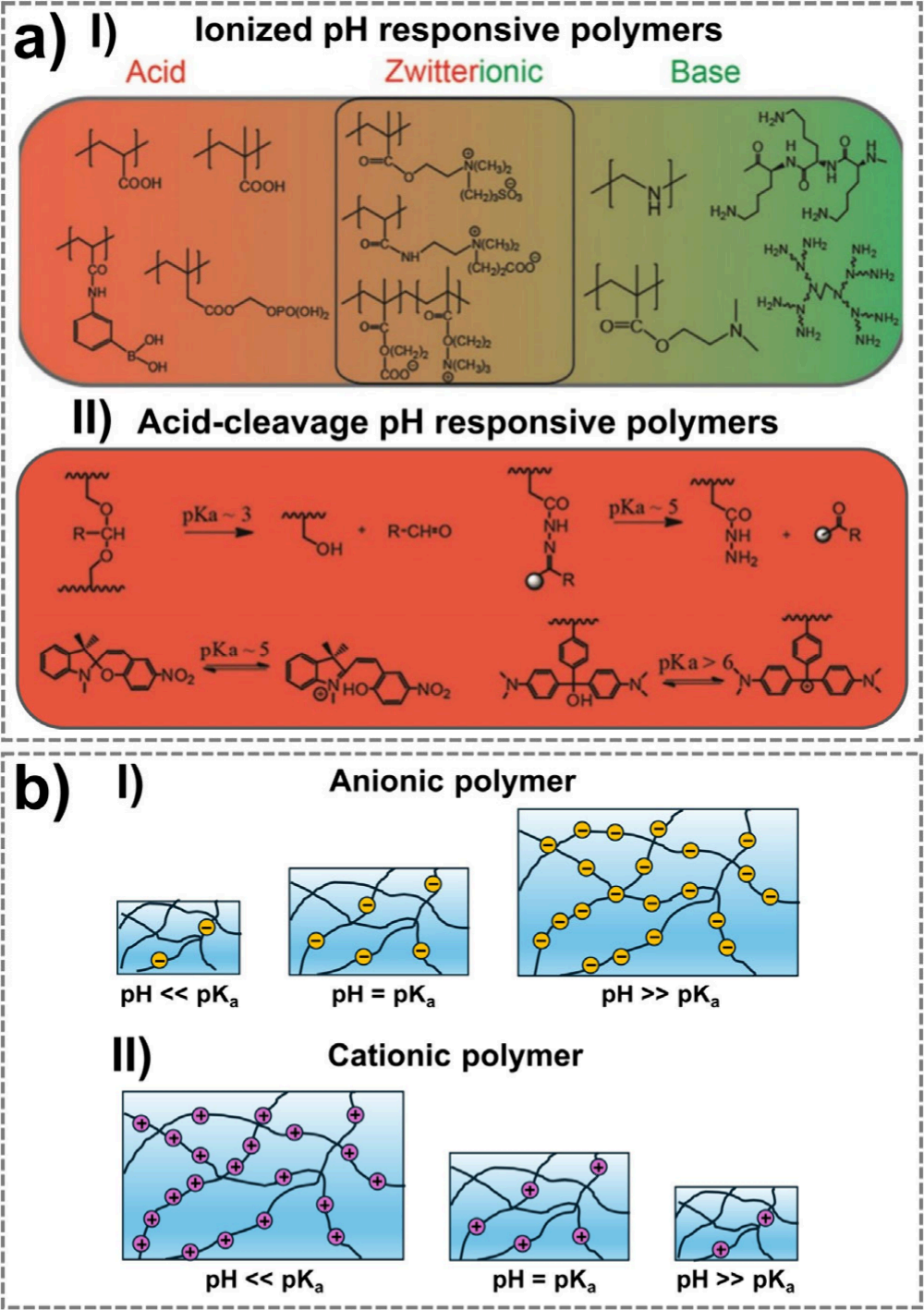
a) Representative pH-responsive polymers: I) ionized pH-responsive polymers; II) acid-cleavage pH-responsive polymers. Reprinted with permission from ref 406 Copyright 2019 Royal Society of Chemistry. b) Sketches showing the pH-driven shrinking/swelling behavior of: I) anionic; and II) cationic polymers.

The most widely used pH-responsive anionic polymers are made from acrylic acid (AAc), methacrylic acid (MAA), boronic acid, and their related derivatives. Anionic monomers have a distinct p*K*_a_, causing the resulting polymers to alter their solvation state and conformation when the pH exceeds the p*K*_a_. In general, when the polymer/hydrogel is immersed in a solution with pH ≫ p*K*_a_, the gel tends to deprotonate, exhibiting a negative charge and undergoing swelling due to electrostatic repulsion. Conversely, when pH ≪ p*K*_a_, the gel protonates, the charges decrease, and the gel tends to shrink (Figure 13a, panel I).^406^ The Young’s moduli of PAA hydrogels vary significantly based on their composition, cross-linking density, and the incorporation of reinforcing agents. For instance, the Young’s modulus for pure PAA hydrogels is approximately 90.2 kPa.^408^ The mechanical properties of PAA hydrogels can be tailored by adjusting factors such as cross-linking density and the incorporation of reinforcing agents. For instance, PAA hydrogels with nanocrystalline cellulose increased the Young’s modulus to about 619.1 kPa, enhancing mechanical strength.^408^

On the other hand, cationic polymers are typically composed of functional groups containing amines, such as amine, amidine, and pyridine. When primary, secondary, or tertiary amine groups become protonated (pH ≪ p*K*_a_), they acquire a positive charge with consequent swelling of the structure by electrostatic repulsion (Figure 13b, panel II). Quaternary amines inherently carry a permanent positive charge. Examples of cationic polymers include polyethylenimine (PEI), polyamidoamine (PAMAM), poly­(2-(*N*,*N*-dimethylamino)­ethyl methacrylate) (PDMAEMA), poly­(dimethyldiallylammonium chloride) (PDMAEMA), along with certain natural polymers.^406^

In addition to anionic and cationic polymers, zwitterionic polymers feature both anionic and cationic groups. These groups can be located on the pendant side chains of distinct comonomer units (referred to as polyampholytes) or within the same monomer unit (referred to as polybetaines). Early studies on polyampholytes date back to the 1950s, including examples such as PMMA-*co*-DMAEMA, PAA-*co*-4-vinyl pyridine (PAA-*co*-4VP), PAA-*co*-DMAEMA, among others.^406^

### 6.2 Material Selection Rationale

Materials for pH-driven soft actuators must exhibit strong and reversible responsiveness, undergoing significant swelling, contraction, or conformational changes in response to variations in acidity or alkalinity. Hydrophilicity and the presence of ionizable functional groups are essential to generate sufficient actuation, while mechanical properties must balance flexibility with structural integrity to avoid rupture during repeated pH cycles. For a detailed overview of the chemical and physical properties of pH-responsive polymers, we refer the readers to tables previously published in a comprehensive review.^409^

For outdoor and environmental applications, materials must withstand broad and uncontrolled pH fluctuations caused by anthropogenic activities or natural events, such as acidic rain (pH ≈ 4) or alkaline industrial discharges (up to pH 10–11). In these scenarios, chemical durability, resistance to leaching, and long-term mechanical robustness under cycling are key selection factors. Responsiveness should also remain effective under variable temperature and pollutant exposure, which are typical in natural and outdoor environments. Here, biodegradable pH-sensitive polymers, such as chitosan, guar gum, alginic acid, hyaluronic acid, carboxymethyl dextran, CMC, gelatin, and tertiary amine starch ether, are advantageous as they minimize ecological impact while enabling devices to function as sensors or adaptive regulators.^409^

When selecting materials for biomedical applications, additional criteria include biocompatibility, nontoxicity, and stability in physiological fluids. Materials should be tailored to the pH ranges of specific organs. For example, they need to have resistance to extreme acidity in the stomach (pH ≈ 2), sensitivity to near-neutral conditions in blood, or adaptability to fluctuating pH in inflamed or tumor tissues. Furthermore, degradation products must be harmless and readily metabolized or excreted, ensuring safe long-term performance. Natural and seminatural polymers such as chitosan, alginic acid, hyaluronic acid, carboxymethyl dextran, and gelatin are particularly promising for these contexts due to their intrinsic biocompatibility and functional versatility. While most examples presented in the following subsection rely on nonbiodegradable synthetic polymers, future designs will increasingly benefit from ionizable, pH-sensitive natural polymers. The adoption of biodegradable pH-responsive materials represents a critical step toward fully sustainable soft actuators and robots, enabling safe use in both the natural ecosystems and the human body.

### 6.3 pH-Driven Soft Actuators and Robots

One of the first examples of an actuator driven by multiresponsive stimuli, including pH, temperature, and light, was reported by Suzuki et al.^410^ The material was a copolymer hydrogel based on a cross-linked network of NIPAM, sodium acrylate, and a trisodium salt of copper chlorophyllin as chromophore. The phase transition behavior in response to pH change (5–9) was a typical first-order phase transition. From that research work, several other pH-based and pH-responsive actuators have been reported over the years, mainly based on hydrogels.^406,411–418^ The hydrogel’s ability to be actuated by pH variations, in addition to other stimuli, enhances its versatility for diverse applications in responsive systems and in complex and dynamic environments.

For this purpose, in 2008, it was reported the first example of a soft robot (Aquabot) based on hydrogel that performed multifunctional operations in aqueous environments driven by pH, temperature, or electric/magnetic field.^419^ The Aquabot had diverse muscle-like locomotive mechanisms as well as integrated organs, including body structures, sensors, and a drug-releasing system. In the case of pH responsiveness, the body of a millimetric Aquabot carrier was based on inert PEG material, while ionic electroactive polymers (electroactive hydrogels hydroxyethyl methacrylate, HEMA) were used for the responsive legs. The pH-responsive legs shrank at acidic pH (∼3) and swelled at basic pH (∼10). Using this expansion/contraction mechanism, the Aquabot could catch, drag (using an external magnet), and release a target. It was fabricated using a photopolymerization method on a PDMS microfluidic platform, and it was capable of sensing, and in perspective, it was conceived for sensing specific organisms and destroying them, preventing the spread of invasive species. Another multiresponsive hydrogel was reported by Thérien-Aubin et al. It was a flat gel sheet able to provide multiple 3D shape transformations triggered by temperature, pH, ionic strength, or CO_2_ supply (Figure 14a).^125^ The primary hydrogel (PG) was composed by poly­(acrylamide-co-butyl methacrylate-*co*-acrylic acid) P­(AA-*co*-BMA) while the binary gel 1 (BG1) was made of P­(AA-*co*-BMA)/poly­(methacrylic acid) (PMAA) and the binary gel 2 (BG2) was composed by P­(AA-*co*-BMA)/PNIPAM. The hydrogels were prepared using a photoinitiator and a cross-linking agent exposed to UV-irradiation through a photomask. Photopolymerization led to the formation of a BG in the light-exposed regions. The SR was determined in pure water as the ratio of gel dimensions at pH = 9.5 and pH = 4, and it was 1.05, 3.2, and 0.92 for PG, BG1, and BG2, respectively. The hydrogels also showed responsiveness to the NaCl concentrations. This work paved the way for producing multiresponsive and programmable adaptable materials with potential applications in soft robotics.

**Figure 14:**
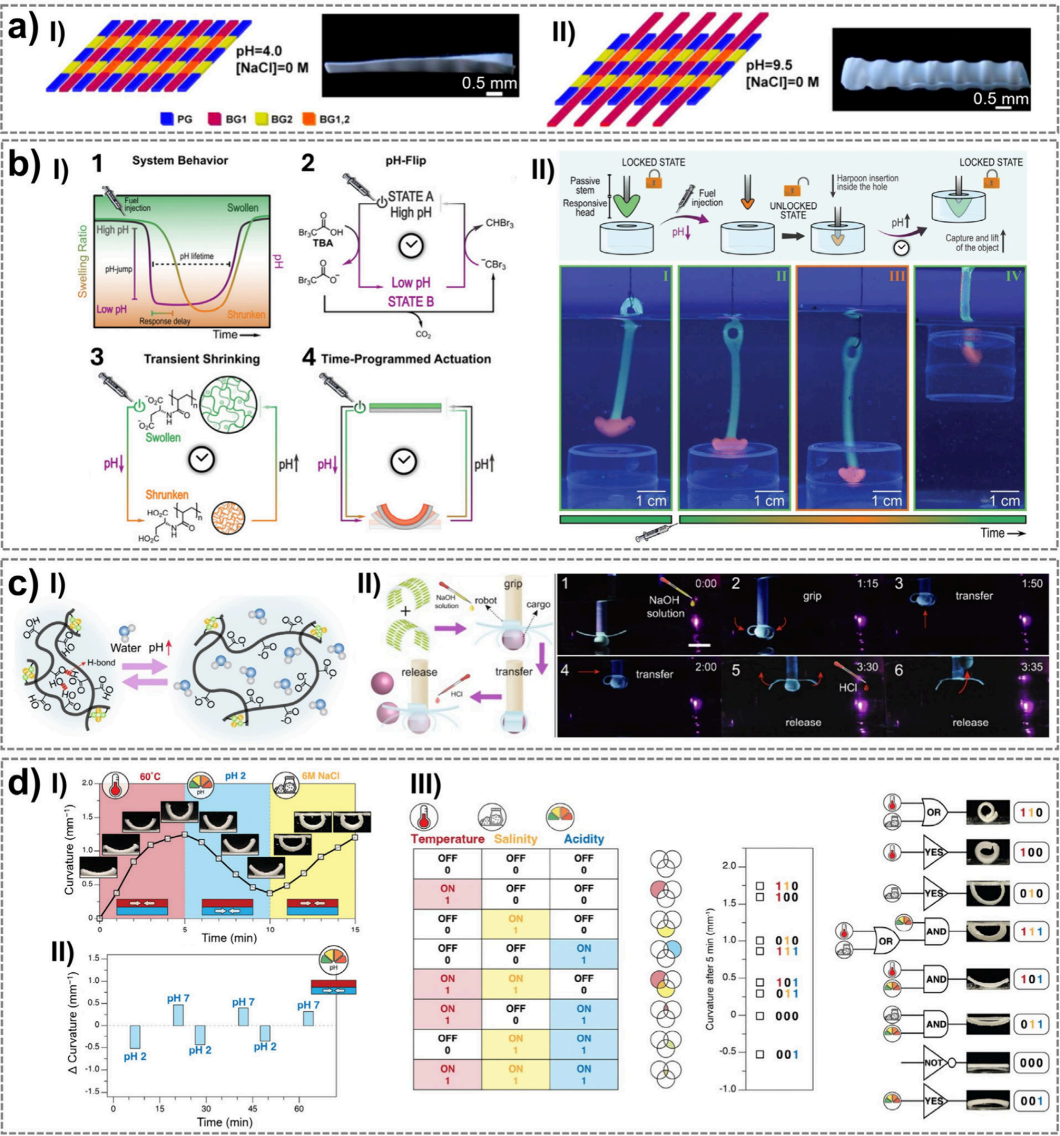
Soft actuators and robots driven by pH variations. a) Multiple shape transformation of gel sheets: I) at pH = 4 and [NaCl] = 0, the as-prepared hydrogel sheet is in a planar shape; II) at pH = 9.5 and [NaCl] = 0, the swelling of BG-1 regions leads to the formation of a long cylinder shape. Reprinted with permission from ref 125 Copyright 2013 American Chemical Society. b) An autonomously operating pH-responsive hydrogel with pH-flip of programmable lifetimes: I) system integration of one-component pH-flips with pH-responsive hydrogels; II) scheme describing the operation of the “fire and forget” harpoon device for autonomous grabbing of objects with small orifices. From left to right: harpoon being lowered over opening, harpoon head resting on opening and unable to spear object, once fueled, head contracts and passes through opening, harpoon being lifted and taking object with it after re-expansion of head. Carried out in 5.0 mM MOPS with 5.0 mM TBA fuel. Reprinted with permission from ref 420 under CC BY NC 4.0, Copyright 2022 The Authors. c) pH-responsive nanocomposites (GelWC) based on zwitterionic monomers and asymmetric CNCs: I) schematic presentation of the swelling/deswelling mechanisms of GelWC in response to pH; II) A GelWC microgripper can grab a spherical cargo by rolling its arms triggered by increasing pH. After transferring the cargo to a new location, the gripper arms are opened, and the cargo is released by decreasing the pH. Scale bar is 10 mm. Reprinted with permission from ref 421 under CC BY 4.0, Copyright 2023 The Author(s). d) Multiresponsive nanocellulose-based hydrogels with embodied logic: I) shape-morphing of a bilayer during sequential exposure to stimuli over time: 0 to 5 min at 60 °C, 5 to 10 min at pH 2, and 10 to 15 min at NaCl concentration of 6 M; II) cyclic actuation of bilayers under pH level variation (pH 2 to pH 7); III) logic operations performed by the bending of the bilayer in response to combinations of stimuli (temperature, salt concentration, and pH). Reprinted with permission from ref 422 under CC BY 4.0, Copyright 2024 The Author(s).

In addition to conventional hydrogels made of polyanions or polycations, hydrogels made with peptides hold significant importance due to their tunable biochemical and mechanical properties. Peptides enhance the hydrogel’s biocompatibility and biodegradability, enabling precise interactions with biological systems, making them ideal for tissue engineering, drug delivery, and regenerative medicine. Narupai et al. reported a 3D printed and biodegradable protein-based hydrogel developed and applied for programmable structural changes under the action of temperature, pH, or enzyme. The hydrogel was based on methacrylated bovine serum albumin (MA-BSA) as the main component for forming stable Pickering emulsions.^423^ The emulsion sensitive to the temperature was created using NIPAM, while the emulsion sensitive to the pH was created using DMAEMA. The emulsion formation resulted in an extrudable ink that was suitable for DIW 3D printing. The pH-sensitive hydrogel was used to fabricate a pH-responsive hydrogel that could reversibly swell and deswell at pH 10 and 2, respectively (40% change in size for 24 h swelling in each buffer). As demonstrator, the 3D printed multimaterial flower (comprised of pH and temperature functionalities) could achieve four different stable states based on the stimuli-responses of the hydrogels. The protein-based hydrogel was a clear example of smart biomaterials for future applications, including smart actuators, drug delivery vehicles, soft robotics, and biomedical devices. Another pH-responsive peptide based on methacrylated fluorescein isothiocyanate derivative (MA-FIID) was designed and realized by Xiang et al. using a PNIPAM backbone to construct, by simple photopolymerization, a bilayer and heterogeneous hydrogel actuators based on the assembly and disassembly of peptide molecules under different pH conditions.^424^ The hydrogels exhibited varying degrees of volume expansion, reaching 63% when exposed to 0.1 M NaOH. Reversible shape changes of hydrogel from 0.1 M NaOH to 0.1 M HCl solution and from 0.1 M HCl to 0.1 M NaOH lasted 25 min, tested under eight cycles, showing great potential for building intelligent biomaterials.

pH-sensitive microgels could be crucial in targeted drug delivery, as they enable precise release of therapeutic agents in response to the pH variations of specific environments, such as tumor tissues or the gastrointestinal tract. Wang et al. reported a smart hydrogel microactuator based on a bionic asymmetric structure inspired by the flytrap.^425^ The bionic microactuator was fabricated by femtosecond laser direct writing (FsLDW) of responsive photoresist containing DMAEMA for 2PP fabrication. When immersed in an acidic solution (pH = 1), tertiary amine groups underwent protonation, gaining positive charges. The resulting electrostatic repulsion between the charged molecular chains caused the polymer network to expand. This expansion facilitated the absorption of additional water molecules, leading to an increase in the hydrogel’s volume and subsequent swelling. Conversely, when exposed to an alkaline solution (pH = 13), the tertiary ammonium ions were deprotonated, reverting to their neutral form. This reduced the electrostatic repulsion and interchain spacing within the polymer, causing the hydrogel to contract and decrease in volume. The deformation and recovery times were 1.2 and 0.3 s, respectively. The *C*_*d*_ (change degree in surface area) was roughly 30% in the presence of an acidic solution. The microactuator was conceived for potential applications in soft robotics, microsensors, and microelectromechanical systems (MEMS).

Gradient structures in hydrogels have been extensively explored as a seamless approach to creating anisotropic hydrogel architectures. These structures eliminate the need for complex multistep fabrication methods and enable efficient shape deformation. Ye et al. developed a novel and straightforward structural programming approach to fabricate anisotropic hydrogels, drawing inspiration from the gradient distribution of dissolved oxygen in seawater and using natural oxygen as the driving force.^426^ The inhibitory effect of oxygen on radical-initiated polymerization, combined with its gradient diffusion behavior in water, facilitated the incorporation of a gradient density network into the hydrogel structure. Additionally, the differing inhibitory effects of oxygen on the radicals of AAc and DMAEMA, influenced by their varying substituent sizes and functional group compositions, contributed to the gradient ratio of the two units within the hydrogel matrix. Furthermore, phase-separated structures, driven by interchain electrostatic interactions of P­(AA-DMAEMA), were embedded in the anisotropic hydrogels during their preparation process. The solutions were poured into the PTFE mold and exposed to the oxygen environment for 10 min for oxygen dissolution in water. Different formulations of hydrogel were prepared depending on the different molar mass ratios of AA to DMAEMA monomers (1:3, 1:2, 1:1, 2:1, 3:1), respectively. Experimentally, the authors found that the 2:1 formulation had the lowest swelling ratio at pH 7 (∼1200%) and the highest swelling ratio at pH 12 (∼2200%) attributed to the electrostatic interactions between polymer chains. The hydrogel was then tested as a gripper, which had a tensile fracture strength of 0.10 MPa before swelling and 0.07 MPa after swelling. The gripper (mass 10.2 g) was able to wrap a piece of copper (mass 2.29 g) and lift it up in 2 min.

Traditional switching methods previously reported are based on manual adjusting of the pH up or down, but they are unable to achieve more intricate autonomous movements. Against this limitation, in 2022, Fusi et al. reported a new method of autonomous control for soft robotic actuators by integrating autonomous chemical controllers in the form of transient pH-flips with pH-responsive hydrogels using a self-decarboxylating acid with hydrogel-based actuators comprising both pH-responsive and non-pH-responsive hydrogel segments compartmentalized.^420^ Tribromoacetic acid (TBA) was chosen as a one-component pH-flip because the resultant transient acidic pH occurred on a suitable time scale (Figure 14b, panel I), while the hydrogel was based on aspartic acid *N*-acrylamide (A^3^) cross-linked in a 50:1 molar ratio with poly­(ethylene glycol)­diacrylate, (PEGDA 6K; MW = 6000 g mol^–1^). The p*K*_a_ value of poly­(A^3^) was ∼ 6.2. The addition of TBA caused a drop in pH below the p*K*_a_ value of the gel, leading to the protonation of the carboxyl groups, and a shrinking of the gel network due to the decrease of electrostatic repulsion between chain segments and less gel–solvent interactions. As the acid fuel decarboxylated and the pH rose, the gels reswelled. This could allow the development of controlled and reversible pH-driven actuators. Autonomous bilayer actuators controlled by transient pH flips and a harpoon device for autonomous grabbing of objects with small orifices (Figure 14b, panel II) were fabricated as demonstrators. The response times were very low, from a few hours to multiple days. The device was capable of “fire and forget” operation, usable in smart implants for the biomedical field.

In addition to programmability, self-healing properties in pH-responsive hydrogels are critical for enhancing their durability and functionality in biomedical and environmental applications. In 2023, Nasseri et al. reported the synthesis of pH-responsive hydrogel nanocomposites with predetermined microstructural anisotropy, shape-transformation, and self-healing properties.^421^ The hydrogel nanocomposite was made of zwitterionic monomers and asymmetric CNCs (Figure 14c). The hydrogel was a copolymerization of 3-dimethyl (methacryloyloxyethyl) ammonium propanesulfonate (DMAPS) and MAA in the presence of CNCs. In the DMAPS-MAA hydrogel, the CNCs were used as reinforcement materials. At high pH values greater than 4.7, the -COOH groups of MAA were ionized, and the charged -COO^–^ groups repel each other, leading to the swelling of the hydrogel, while at lower pHs this process was reversed. At pH 12, the maximum swelling degree *ΔL*/*L*_0_ = 70% was reached. The time scale of both shape deformation and recovery was around 5 min. The tested samples were prepared by casting, but the hydrogel showed extrudable properties compatible with perspective 3D bioprinting techniques. The hydrogel showed self-healing properties and had an elastic modulus of ∼ 30 kPa. For soft robotic applications, the authors developed a microgripper driven by pH changes. The proposed material and its engineering could be employed in the development of biomedical soft robots.

pH-sensitive hydrogels have numerous applications in the biomedical field due to their ability to respond to specific pH changes. Zhang et al. designed and developed a bionic robot drawing inspiration from the octopus, from its unique form and the adhesive capabilities of its tentacles.^427^ The device was specifically designed for precise stimulation and pH monitoring in the cervical region. The hydrogel actuator had three layers: 1) a middle layer made of copper particles for photothermal response; 2) a top responsive hydrogel layer sensitive to pH; 3) a bottom PDMS layer (inactive). At pH above 4.3, the AAc molecules in the hydrogel layer underwent ionization, resulting in an increased swelling compared to the hydrogel at pH 2.9, and showing a maximum swelling at pH 8.2. Through the integration of copper particles in the middle layer and the presence of inverse opal photonic crystals, the octopus-inspired actuator could also exhibit multifunctional actuation through photothermal actuation with a NIR light (2.6 W cm^–2^) and colorimetric sensing of the pH. The actuator could also be applied to a variety of medical applications.

The fabrication of biodegradable soft actuators that exhibit synergistic color and shape changes in response to environmental pH changes could provide real-time visual feedback alongside mechanical adaptability, enabling applications in areas such as soft robotics, wearable sensors, and biomedical devices, but remaines challenging. Shi et al. created a soft actuating gel made from carbon dots (CDs) chemically cross-linked with SA.^428^ The gel was coated on the PLA tape to form a gel/PLA bilayer. The gel showed fast, synergistic changes in both color and shape when exposed to pH variations, due to the protonation and deprotonation of the CDs. The colors were: turquoise-green in color at pH 7, but gradually changed to yellow-green, yellow, and orange-yellow as the pH was lowered to 5, 3, and 1, respectively, and to yellow, yellow-green, and green as the pH was raised to 9, 11, and 13, respectively. Similarly, the hydrogel also deformed in 20 s at pH 5, exhibiting the largest deformation, while decreasing deformation was recorded moving to pH 1 and pH 13. The soft actuators could carry out tasks like grasping and lifting, while also exhibiting self-color changes for camouflage and signaling purposes. At pH 5, grasping was observed in 10 s in response to pH. The grasping robot wrapped, grabbed, and transported a plastic block in approximately 29 s. The actuator was degradable and exhibited synergistic changes in color and shape, and it was expected to promote further research into sustainable soft actuators. In addition, Arsuffi et al. designed a partially bioderived composite material, responsive to multiple stimuli (such as temperature, pH, and salinity), enabling programmability in both space and time and computation of logic operations (Figure 14d).^422^ The material was based on PNIPAM or PAA alongside SA, and reinforced with CNC at 14 wt% and CNF at 1 wt%. It was processed through DIW 3D printing. Ionic cross-linking with Ca^2+^ was applied to the SA network, while photopolymerization was employed to cross-link either the PNIPAM- or the PAA-based networks. PAA/SA/CNC hydrogel shrank when submerged in acidic solutions with a pH below PAA’s p*K*_a_ (4.3), reaching nearly maximum shrinkage in less than 30 min. The shrinking rate of the PAA/SA/CNC hydrogel increased as the pH level decreased. At pH = 2 the water loss was roughly 75% after 1 h. The hydrogel was also responsive to NaCl concentrations: the shrinking rate of PNIPAM/SA/CNC hydrogel increased with salt concentrations in the solution. For example, after 1 h in a 6 M NaCl solution, the hydrogel showed a 32% reduction in water content compared to its swollen state. The bilayer developed from the two hydrogels was able to show several cycles of reversible pH actuation in an hour (Figure 14d, panel II). In addition, the multiresponsiveness of the bilayer enabled a multivalued logic gate behavior of the bending actuator, which converted the 8 logic combinations of the three stimuli (ON and OFF for each stimulus) into a single-valued curvature change (Figure 14d, panel III). This demonstrated the potential of using multiresponsive hydrogel actuators as physical, embodied logic gates in autonomous soft robotics. In Table 8 we report on the fabrication process, functional materials, performance under pH variations, energetic sustainability classification, and applications of pH-driven soft actuators and robots. For energetic sustainability, actuators and robots with an operation pH range in 4–10 for environmental applications or 2–8 for physiological applications are considered fully sustainable, while those with actuation pH requirements partially align with and outside of the sustainable pH range are considered as partially sustainable and laboratory-level, respectively. Systems that are pH-responsive but require external injection of pH changing media are categorized as hybrid-driven.

**Table 8:** pH-Responsive Soft Actuators and Robots

**Soft actuator and robot**	**Materials and fabrication process**	**Performance under pH variation**	**Energetic sustainability classification**	**Applications**	**Ref**
**Aquabot**	Body: PEG; legs: electroactive hydrogels HEMA.	HEMA legs shrink at acidic pH (≈3) and swell at basic pH (≈10).	Laboratory-level	Sensing organisms against invasive species.	^419^
	Fabrication: photopolymerization on a PDMS microfluidic platform.				
**Multifunctional shape-morphing sheet**	PG: P(AA-*co*-BMA); BG-1: P(AA-*co*-BMA)/PMAA; BG-2: P(AA-*co*-BMA)/PNIPAM.	SR in pure water as the ratio of gel dimensions at pH = 9.5 and pH = 4: 1.05, 3.2, and 0.92 for PG, BG1, and BG2.	Fully sustainable	Multiresponsive and programmable soft robotics.	^125^
	Fabrication: photoinitiator + cross-linking agent exposed to UV-irradiation through a photomask.				
**3D printed protein-based hydrogels**	pH-sensitive hydrogel: DMAEMA.	Swell and shrink at pH = 10 and 2, respectively (40% change in size for 24 h swelling in each buffer).	Laboratory-level	Drug delivery vehicles, soft robotics, and biomedical devices.	^423^
	Fabrication: 3D printing.				
**pH-responsive peptide hydrogels**	PNIPAM assembled with peptide molecules under different pH conditions.	Volume expansion of 63% in 0.1 M NaOH. Reversible in 8 cycles, each of 25 min.	Laboratory-level	Intelligent biomaterials.	^424^
	Fabrication: molding and photopolymerization under UV light.				
**Flytrap-inspired pH-driven 3D hydrogel**	Responsive photoresist containing DMAEMA.	Deformation and recovery time when pH changes between 1 and 13: 1.2 and 0.3 s. Surface area change of 30% in acidic solution.	Laboratory-level	Soft robotics, microsensors, and MEMS.	^425^
	Fabrication: FsLDW.				
**Gradient hydrogel**	AAc and DMAEMA hydrogels.	AAc to DMAEMA monomers (2:1): SR ≈ 1100% at pH = 7, SR ≈ 1600% at pH = 2, and SR ≈ 2200% at pH = 12.	Laboratory-level	Actuators, grippers.	^426^
	Fabrication: molding and exposition to O_2_ for 10 min.	Gripper (mass 10.2 g) can wrap copper (mass 2.29 g) and lift it up in 2 min.			
**Autonomous soft robots empowered by chemical reaction networks**	A^3^ cross-linked in a 50:1 molar ratio with PEGDA, and TBA as a one-component pH-flip.	Low response time: from a few hours to multiple days. The pH ranges from 3 to 7.	Hybrid-driven	“Fire and forget” operation for smart implants in biomedical fields.	^420^
	Fabrication: molding and photocuring				
**Programmable and self-healing hydrogel**	Zwitterionic DMAPS and MAA in the presence of CNCs.	Maximum swelling degree *ΔL/L*_0_ = 70% (at pH = 12)	Laboratory-level	Biomedical devices, grippers.	^421^
	Fabrication: casting.				
**CDs cross-linked hydrogel**	CDs-SA on PLA tape.	Color change and deformation in the pH range 1–9, with the maximum deformation occurring at pH 5.	Partially sustainable	Biodegradable and sustainable soft robots.	^428^
	Fabrication: chemical cross-linking and coating.				
**Multiresponsive nanocellulose-based hydrogels**	PNIPAM or PAA alongside SA, + 14 wt% CNC and 1 wt% CNF	At pH = 2, water loss ∼ 75% after 1 h. Reversible bending curvature change from – 0.5 mm^–1^ (pH = 2) to 0.5 mm^–1^ (pH = 7).	Fully sustainable physiologically, partially sustainable environmentally	Multiresponsive actuators, embodied logic gates in autonomous soft robotics.	^422^
	Fabrication: DIW.				

Although technologically and scientifically interesting and representing a sustainable energy source, we must note that, to the best of our knowledge, pH-driven soft systems are still limited to simple actuator designs or shape-morphing structures, which have not been realized as more practical soft robots with reversible motions. Most demonstrations reported above are at the proof-of-concept stage in laboratory conditions under controlled, anthropogenic pH variations. For environmental applications, the sensitivity of pH-responsive actuators and robots makes them particularly relevant for detecting and responding to environmental pollutants, such as acidic rain. In such scenarios, they could serve as adaptive indicators or even smart regulators capable of autonomously opening and closing to protect sensitive systems or modulate environmental exposure. While pH in healthy or polluted aquatic environments can vary in the range of 4–10, most currently reported actuators operate under more extreme acidic or alkaline variations that are considered partially sustainable or only laboratory-level. On one hand, this demonstrates good stability of the materials under more extreme pH conditions; on the other hand, it represents a practical limitation since such wide variations cannot be achieved rapidly in natural environments. For this purpose, future research on pH-responsive materials should focus on designing new materials capable of maximizing shrinking and swelling within the 4–10 pH range as sustainable actuating systems. Another field where pH-driven actuators and responsive robots may be particularly well-suited is the biomedical field, such as in drug delivery systems and grasping devices.^429^ For these purposes, the operation pH range of the soft systems must overlap with the physiological pH range 2–8 to be considered as sustainable and functional. The distinct pH in different organs and localized pH variations in pathological conditions like inflammation or tumor microenvironments offer opportunities for site-specific actuation or controlled therapeutic release. This organ- or site-selective responsiveness could enable precise targeting and minimize side effects compared to conventional delivery approaches.

## 7 Triboelectric Energy and Generators for Soft Robotics

### 7.1 Charging through Contact of (Soft) Materials

Robots continuously interact with their environments, and this includes transient contact between their soft, solid, or liquid materials and the robot’s surroundings. These simple interactions can be a source of static electricity and surface charges due to the phenomenon of contact and triboelectrification.^430–432^ The effect occurs on most material surfaces and is an energy conversion from a mechanical input to an electrical output with high potential to exploit the “crumbs of electricity” which would otherwise be lost. It is caused by a transfer of electrons, ions, liquid, and electric double-layer residues, or materials during the typically transient and nonequilibrium interaction.^431,433,434^ Consequently, it is a material-dependent phenomenon for which the pair of materials that come into contact is crucial, as it determines charge distribution and which material gathers net positive and which net negative charges. The triboelectric series orders materials in their tendency to charge with a certain net polarity and can be a useful guide for selecting triboelectric pairs.^435–437^

The materials of the contacting surfaces, and the way the mechanical contact occurs (sliding, tapping, rotation, etc.) can be engineered to systematically enhance charge formation, which is used in so-called triboelectric energy generators (TEGs) or triboelectric nanogenerators (TENGs), in the following, for simplicity, called TENGs.^438^ The devices harvest these charges by implementing an electrode in the vicinity of the charged surfaces into which the static surface charges are electrostatically induced.

### 7.2 Triboelectric Materials and Operation Modes

#### 7.2.1 Standard Triboelectric Materials

The materials in TENGs are, compared to other energy harvesting approaches, relatively simple. In most cases, halogenated, especially fluorinated or chlorinated polymers like fluorinated ethylene propylene (FEP), polytetrafluoroethylene (PTFE), polyvinylidene fluoride (PVDF), and PVC are common net negatively charging materials. However, one of the most important polymers in soft robotics, silicones like PDMS or EcoFlex, also tend to charge strongly negatively. As mentioned before, the pairing surface is crucial for increasing charge formation. Combining such net negatively charging polymers with materials that tend to charge positively, like PI, nylon, strongly increases charge formation and related voltage generation, etc.^433,434,439^ How a specific material pair enhances charge formation is still under discussion. The outcome of experimental and theoretical studies strongly depends on environmental conditions, contact forces, and materials involved, resulting in potential electron, ion, and material transfer during the transient contacts.^440^ Moreover, triboelectrification occurs on most material surfaces, as such also biological surfaces spontaneously charge significantly upon contact or may have interesting structures to be mimicked in artificial TENGs. Structures like skin or plant leaves have been used to improve TENGs.^441–445^ More examples of specifically tailored material systems are given in the following sections.

#### 7.2.2 Induction Electrode Materials

The dielectric and tribo-active materials on which the static surface charges form are deposited on application-specific electrode materials. Most electrodes can be used, and electrodes from copper, aluminum, gold, and other metals to hydrogels, transparent indium tin oxide films, conductive polymers, and ion-conductive living tissue have been employed.

#### 7.2.3 Mechanical Excitation and Operation Modes

Repetitive contact and separation are required between the material pair to form, measure, and use the surface charges produced by triboelectrification. The forces, frequency, and complexity of the motion depend on the source of mechanical energy. It is possible to exploit mechanical energy occurring in the direct environment of the TENG, like body motion (including heartbeats and breathing),^446,447^ airflow and wind,^448–450^ ocean waves,^451^ sound waves,^452,453^ raindrops,^454,455^ and many more, to convert them into electricity that can drive electronic devices, especially sensors, but also actuators or electrochemical systems. In the simplest case, a TENG consists only of one electrode and a triboelectric active material that is touched (single electrode mode, Figure 15a), e.g., sufficient for tactile sensing during a grasping motion. Dual electrode and rotational modes need more complex assemblies (Figure 15a). In the last years, multiple examples have been presented that make TENGs interesting as autonomous power sources for specific parts of soft robots. The operation modes, either integrated in the robot (often used for sensing) or as an external, tethered power supply (often used for actuation), are schematized in Figure 15b and detailed later. A great advantage is that in many cases, simple materials or already existing structures like the silicone body of a soft robot are suitable to achieve sufficient energy conversion and charge formation.

**Figure 15:**
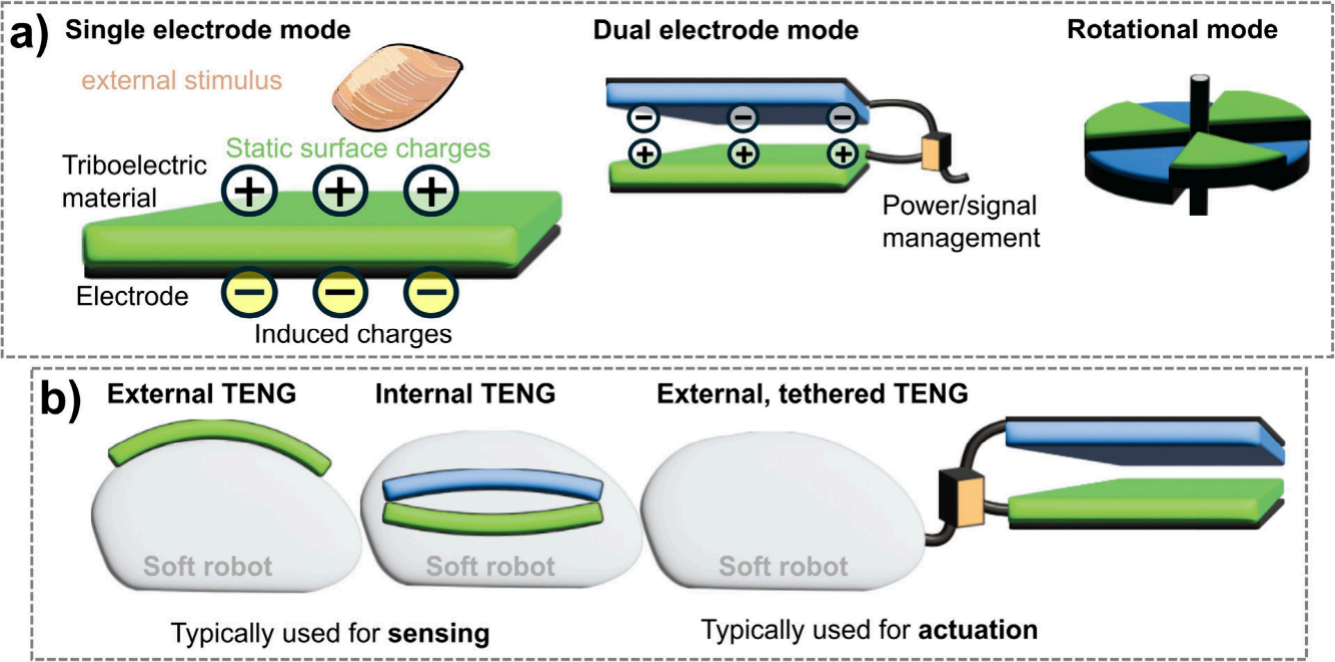
TENGs as environmental energy harvesters for robotics. a) Overview of the most common operation modes of TENG-based sensors and energy harvesters for robotics. b) Example of the most common integrations in robotics and soft robotics as external, skin-like TENGS, internal TENGS, and external, tethered TENGs for sensing and actuation.

### 7.3 Triboelectric-Powered Sensors for Soft Robots

TENGs can enable various sensing tasks in soft robots by exploiting the charges generated when the robot or its sensor components interact with the surrounding environment, and this section focuses specifically on TENG sensors integrated into robotic systems. The electrical charges generated can serve, in the simplest case, as a binary signal giving information on when a contact event is occurring in a single electrode TENG configuration. The output voltage is typically force-dependent, which also allows to derive quantitative tactile feedback. However, the sensing tasks can also be more complex and contain, for example, information on the contact force and pressure or even the materials due to the material-dependent charge formation. Even chemical sensing, like that of mercury detection down to 3 nM detection limit, integrated in a robotic finger, could recently be realized due to the change of charge and voltage generation when material properties change.^456^ Dynamic forces like wind can also be sensed by TENGs.^448^

For these sensing tasks, the generated voltages do not need to be specifically high (see max, output ranges in Table 9), but selected triboelectric materials, especially silicones or PTFE can easily generate high surface voltages. The high voltages are an advantage of TENGs that help to obtain sufficient sensing signals even for very small forces, such as from acoustic waves,^441,452,453^ eye motion,^457^ or liquid flow in microchannels,^458^ to mention only a few of many examples from the past decade. It is important to note that while triboelectricity generates the sensing signals, the readout circuitry typically still needs to be powered separately. Only relatively simple voltage readouts and wireless transmission protocols can be powered simultaneously by the TENG.

**Table 9:** Examples of the Diverse Applications and Performance of TENGs for Sensing in Robotics

	**Application (robotic use case)**	**Triboelectrically active materials**	**Electrode materials**	**Mechanical structure and mechanism**	**Max output voltage, current, and/or power**	**Sensitivity in specific applications**	**Ref**
**Biomedical**	Cardiac and blood pressure sensor, bioresorbable (biohybrid: in vivo sensor)	Nanostructured PLA/chitosan (4%)	Mg	Layered structure with an air spacer actuated by cardiac motion	Voltage 4.2 V @ 20 N, during in vivo operation a few mV	11 mV mmHg^–1^	^447^
	Hearing aid (auditory system for robots)	Ion-etched FEP	Au	Layered structure actuated by acoustic wave-induced vibrations	Voltage 1.2 V @ 100 dB sound pressure	110 mV decibel^–1^ at 100 to 5000 Hz	^453^
	Respiratory sensing, bioresorbable (biohybrid: in vivo sensor)	Nanostructured PLA/chitosan/SA films	Fe	Layered structure actuated by respiratory motion	Voltage 9.2 V @ 20 N, during in vivo operation, a few 10 mV	22.61 mV mmHg^–1^	^469^
	Sensor in a soft robotic stomach simulator	PI	Al, Cu, Ag paint	Zig-zag origami structure	Voltage ∼ 70 V	0.9535 V%^–1^ contraction of the stomach simulator	^470^
**Skin-like**	Tactile sensing (integrated in grippers)	Microstructured PDMS	Al	Single electrode mode TENG combined with a potentiometric sensor	Voltage ∼ 1 V	20 mV N^1–^ for triboelectric tactile sensing mode, 0.01–10 N range	^461^
	Texture sensing for soft fingers (integrated in grippers)	Buckling-textured EcoFlex 20 and Dragon skin 10	Cu	Single electrode mode TENG combined with a capacitive and electromagnetic resonance sensor	Voltage ∼ 4 V	Machine learning analysis of multiple sensor signals allowed selected object recognition with 98.43% accuracy	^471^
	Proximity and pressure-sensing skin (integrated in soft grippers and walkers)	EcoFlex 30	Ag flakes	Single electrode mode TENG	Voltage ∼ 30 V	0.29 kPa^–1^ (9.54 V kPa^–1^)	^472^
	Tactile sensing (stand-alone TENG electronic skin)	PTFE and poly diacetone acrylamide, hierarchical structure	Agar/PAAm hydrogel	Single electrode mode TENG in contact with PTFE	Voltage ∼ 250V	2.89 V kPa^–1^	^442^
	Tactile sensing (finger-worn stand-alone TENG sensor)	PDMS, PVDF-HFP, fingerprint-like microstructure	Ag nanowires	Single electrode mode TENG	Voltage ∼ 80 V @ 10 N, ∼ 10 V @ 0.2 N	5.84 V kPa^–1^	^473^
	Multifunctional tactile sensing (integrated in a glove and robotic hand)	PDMS, ZnO nanostructures	Graphene	Single electrode mode TENG for “material” sensing	Voltage up to ∼ 0.4 V	0.0146 V kPa^–1^ in contact with FEP film	^468^
	Tactile sensing, texture recognition, soft robotic skin (integrated in soft grippers)	Liesegang-patterned PVDF-HFP-TFE	PAAm hydrogel	Single electrode mode TENG, patterned surface used for texture recognition, combined with resistance sensing	Voltage up to ∼ 80 V	1.50 V kPa^–1^	^464^
	Tactile sensing, stretchable electronics (integrated in griper)	Silicone: Ecoflex 30	Liquid metal EGaIn	Single electrode mode TENG	Voltage 25 mV @ 0.2 N	10 mV kPa^–1^	^474^
	Tactile sensing, sound, and ultrasound sensing in air and underwater (integrated in glove)	Nanoporous microridged P(VDF-TrFE) and PDMS	Ag	Two-layer TENG, layer separation through microridges	Voltage up to 60 V, power ∼ 46.7 μW cm^–2^	0.55 V kPa^–1^	^441^
	Electronic skin, tactile sensing (stand-alone, tested on human hand)	PDMS Sylgard 184 and VHB 9469	PAAm-LiCl hydrogel	Single electrode mode TENG	Voltage up to 6 V @ 101.2 kPa	0.013 V kPa^–1^	^475^
**Soft grippers**	TENG for tactile and length sensing (integrated in grippers)	PTFE, silicone	Cu, Ni-fabric	Single electrode mode TENG with patterned electrode for multiple sensing points, AI signal interpretation	Voltage ∼ 1–3 V	∼97% object recognition accuracy (28 different shapes)	^476^
	Touch sensing, humidity-responsive (integrated in grippers)	PVP/PAA, MIL-88A metal organic framework nanofibrous film	Conductive fabric	Single electrode mode TENG	Voltage ∼ 54 V PTFE contact, ∼ 4 V PA contact @10 N, up to 1 V during touch sensing		^465^
	Tactile sensing (integrated in grippers)	PTFE, PI	Cu	Single electrode mode TENG with patterned electrode for multiple sensing points	Voltage up to ∼ 3 V		^477^
	Motion, bending, material sensing (integrated in grippers)	PDMS with customized composition	Printed Galinstan	Single electrode mode TENG	Voltage up to 25 V @ 112 kPa	0.308 V kPa^–1^, 14 nA kPa^–1^, 96% accuracy of movement detection	^478^
	Digital twin, tactile sensing, contact position sensing, object identification (integrated in grippers)	PTFE, PDMS	Cu, Ni-fabric	Single electrode mode TENG with patterned electrode for multiple sensing points, AI object recognition	Voltage 1–6 V during operation	98.1% gripped object recognition accuracy (16 objects)	^479^
**Various**	Flow and chemical sensor, touch sensor (stand-alone, tested in textiles)	PDMS	KCl solution, Pt	Liquid electrolyte flow through PDMS microtube	Tens of pA peak-to-peak current for KCl sensing		^458^
	Selective mercury (Hg^2+^) sensing, chemical pollutant sensing (integrated in robotic hands/fingers)	Te nanowires	Al	Te nanowires form HgTe nanowires in contact with Hg^2+^, this reduces the output signal from liquid-solid contact electrification	Voltage tens of mV	3 nM Hg^2+^ detection limit, linear range 10 nM to 10 μM	^456^
	Tactile sensing antenna (integrated in robotic insects)	Silicone sponge, Ecoflex 30	Ag nanowires	Lightweight TENG sponges on the tip of antenna electrodes sense touch with objects	Voltage ∼ 1 V	1.84 V N^–1^ for forces < 0.8 N	^467^

#### 7.3.1 TENG Sensors Integrated in Robotic Devices

Table 9 gives examples of TENG sensors that have been integrated in robotic and soft robotic devices, sorted by application fields: biomedical, skin-like, soft-gripper, and other examples. The table gives an essential overview of applications and use cases in soft robotics, the exploited triboelectrically active materials as well the electrode materials, the mechanical structure and motion mechanism, the resulting maximum output voltage range, current and/or power metrics, and the sensitivity in their specific application. Further examples of triboelectric sensors have recently been reviewed.^433,434,459,460^ The majority of examples in Table 8 provide just a proof-of-concept sensing task in a soft robotic application scenario. Few studies really show performance in application-relevant scenarios and over typical operational times. Moreover, TENGs are often combined with other sensors, like resistive, capacitive, and potentiometric sensing, to add additional functionality and improve reliability and sensitivity. Figure 16a shows an interesting example of a multilayer structure with combined TENG and potentiometric sensing that was used to mimic the slow and fast adapting components of touch sensing by skin.^461^ Using a combination of sensing approaches that use different but complementary mechanisms can improve the performance, either backing each other up or extending the sensing capabilities to multiple signals.

#### 7.3.2 Materials in TENG Sensors

The examples cited in Table 9 show that a wide variety of functional polymers and composites have been explored for the fabrication of TENG sensors, aiming to enhance charge generation, flexibility, and sensitivity. Commonly used elastomers include PDMS (in various microstructured, nanopatterned, or composite forms), Ecoflex (20 and 30), Dragon Skin 10, and silicone sponges, which provide mechanical compliance and surface patternability. Fluoropolymers such as PTFE, FEP, PVDF, and PVDF copolymers are frequently employed for their strong electron-withdrawing properties, often combined with hierarchical or biomimetic surface structures to increase effective contact area. Additional strategies include incorporating inorganic nanostructures (e.g., ZnO, Te nanowires) or metal–organic frameworks (e.g., MIL-88A) to increase surface charge density or for material-specific effects as detailed below, and using biopolymer-based films such as nanostructured PLA/chitosan or PLA/chitosan/SA to achieve biodegradable designs.

#### 7.3.3 External Conditions Affecting the Sensing Performance

Triboelectric charging is on most materials, especially affected by external conditions like humidity (increases adsorbed water and air conductivity, leading to reduction in charge build up and quicker charge dissipation), surface history, material wear, and other difficult-to-control external factors. Consequently, especially long-term performance under application-relevant conditions, particularly for more complex tasks such as material recognition, still requires further fundamental understanding and further research. An approach is encapsulating the TENG^462^ and isolating it from the environment can help to reduce environmental effects, but this makes the assembly more complex. Due to the intricate charge formation in TENGs, approaches to consistently characterize TENG sensors are still lacking, but this would enable better comparison between existing devices and help define new performance goals.

#### 7.3.4 Materials Engineering Improving TENG Sensors

A way to counterbalance performance uncertainty and add further functions is materials engineering. Among various functionalization techniques to improve TENG performance, which have been reviewed by Wang et al.,^463^ we highlight here particular examples for a robotics context. Tailored patterning combined with chemical functionality is an approach to enhance the TENG sensing performance, in addition to the combination with other sensing principles as mentioned above. Chemically interesting approaches like Liesegang patterning produce fingerprint-like ridges in soft materials (Figure 16b), obtained through reaction-diffusion-mediated nonequilibrium-growth of conductive polyacrylamide/K_2_CrO_4_ hydrogels. These patterns create surface features that enhance triboelectric signal formation but also introduce features that assist the fingerprint-like sensing of surface structures in robotic grippers.^464^ Moreover, another exciting approach is using materials that change their triboelectric charging behavior upon reaction with heavy metal ions, like Te nanowires. Especially, the presence of Hg^2+^ decreases the output voltage of the TENG, which was recently combined as chemical sensors on a robotic hand for water analysis and other applications (Figure 16c).^456^ Because many different materials can generate triboelectric charging, TENG sensing offers greater flexibility than other sensing techniques. This makes it easier to integrate additional functions, such as material-based humidity-driven actuation, into the same system. As depicted in Figure 16d, this combination has recently been shown in a humidity-responsive gripper combined with a TENG-based tactile sensing.^465^ It is also straightforward to miniaturize the sensors, for example, using bioinspired approaches like whiskers^466^ and antennas, and this has been used to build an integrated, single-electrode-mode contact sensor in robotic insects^467^ such as shown in Figure 16e. Obviously, the signal interpretation in all TENG sensors is important, and often adequate referencing is needed to obtain reliable signals. Signal analysis can be assisted with artificial intelligence (AI), which could improve sensing performance and enable more complex tasks like object recognition using the material-dependency of the triboelectric charging and surface structure dependency of the signal frequency.^468^ We focused these highlights on TENG sensors already integrated in robotic devices, but most TENG sensors in the literature are still standalone or single proof-of-concept devices. Despite the straightforward implementation, further research is clearly required to make TENG sensing reliable in more complex robots, in long-term, and in unstructured environments.

### 7.4 Actuation Driven by Triboelectricity for Robotic Systems

Sensing by TENGs is relatively straightforward and has been the primary focus of the exploitation of TENGs in robotics and soft robotics, but it has also been demonstrated that TENGs can directly power actuation.

#### 7.4.1 Dielectric Elastomer Actuators (DEAs)

A high-voltage TENG can produce outputs of several kV, and this can be tethered to DEAs.^480–482^ DEAs are often realized by acrylic films and compliant electrodes based on carbon or silver grease. Powering complex motion such as crawling (Figure 16g),^483–485^ liftoff of a microaerial robot (Figure 16f),^486^ and pumping^487^ has been shown using TENGs.

#### 7.4.2 Other Electromechanical Actuators

Besides DEAs, other electroactive polymer actuators, such as ionic polymer–metal composites (IPMCs) have been powered by an externally tethered TENG, for which rather a charge transfer of ∼ 9 μC than high voltages was required to obtain motion of the thin film actuators.^488^ The system could, for example, grasp an object. Moreover, the charges generated by TENGs can also drive liquid manipulation^489^ and electrohydrodynamic pumping.^490^ The latter was recently impressively used to power soft robotics, for example, directly powering a pneumatically driven soft gripper.^490^ It is important to note though, that in all these examples, an external TENG is tethered to the robot. The integration of TENG-based actuation within the robot body remains a challenge to be addressed in future work.

#### 7.4.3 Materials in TENGs for Actuation

The materials used in the TENGs mentioned above are often standard, commercial polymers like often used FEP, PTFE, PI (Kapton), PA (nylon) frequently treated with reactive ion etching to increase the charge yield. A major issue is sufficient voltage and charge generation, and further improvements in materials triboelectric charge yields could make external TENGs smaller and easier to integrate.

#### 7.4.4 Mechanical Excitation of TENGs for Actuation

As mentioned above, in recent proof-of-concept studies, TENGs have not yet been integrated directly into the robot body. In these early-stage demonstrations, the TENGs are not driven by the robot’s own motion or by mechanical excitations acting directly on the robot. Instead, they are typically powered by external TENGs that are tethered to the actuators or the robot. From an energetic sustainability perspective, this reliance on externally driven excitation places most existing TENG-powered soft robotic actuators in the hybrid-driven or laboratory-level category. Even if some TENG-powered sensors entirely produce the sensor signal in a self-powered manner in the environment (e.g., in vivo in animals, on plants, etc., see Table 9), the signal read-out, signal transfer, and analysis are often powered by additional energy sources that are not fully sustained by the energy harvested from the surroundings. While TENGs can harvest energy from environmental sources such as wind, ocean waves, or body motion, fully integrated devices that use TENGs as an “embodied energy source”,^491^ harvesting environmental mechanical energy to power robotic actuation, to the best of our knowledge, have not yet been demonstrated. Achieving this will require overcoming key challenges, particularly developing TENGs capable of producing sufficiently high voltage and charge outputs from the irregular and inconsistent motions typical of environmental energy sources. Some research works are combining other actuation sources, such as humidity-responsive materials with TENG sensing, as mentioned before (Figure 16d).^465^ Moreover, it was indeed shown that a robot whose actuation is driven by other actuation strategies may also collect electricity through its body’s interaction with the environment and transfer it to other devices.^492^ Thus, new materials are needed that enable high output TENGs from environmental energy, ideally integrated in the robot as well as materials, less sensitive to being affected by environmental conditions as well as, combinations of multifunctional systems that enable autonomous actuation together with TENGs.

### 7.5 Biohybrid Triboelectric Systems: Driven by Living Organisms

Because triboelectric charging occurs on most materials, biological surfaces in organisms can be directly exploited as active components of TENGs.

#### 7.5.1 Biohybrid TENGs

Biohybrid TENGs integrate living organisms such as plants or animals as part of the energy harvesting systems, leveraging natural movements and the surface properties of tissue, skin, or leaf surfaces. Several TENGs have been used in vivo for harvesting biomechanical energy of heartbeats, breathing motion, etc.,^447,452,469,495–497^ mostly for sensing. TENGs can also be externally powered through the tissue, e.g., by using ultrasound as an energy source that can be externally applied (example is given in Figure 16h).^452^ The organisms’ interfaces and surfaces are thereby often acting as the tribo-active material that charges. In some cases, such as with plants (as detailed below), the plant surface is used for triboelectric charge generation, while the ion-conductive tissues serve as electrodes where the triboelectric charges are electrostatically induced. This approach reduces the complexity of the TENG assembly while increasing the eco-friendliness of the materials used.

#### 7.5.2 Plant-Based TENGs

TENGs based on plants can generate electricity from the passive motion of leaves vibrations due to wind or raindrops.^449,498–500^ It was shown that wind at a low speed of ∼ 3 m/s moving plant foliage can be harvested and continuously power a wireless humidity and temperature sensor for several days (Figure 16i, left panel shows the implementation).^494^ Other plant-hybrid systems implement energy harvesting from wind and raindrops approaching devices, which can autonomously harvest energy outdoors.^494,498,499,501–504^ Indeed, while air humidity can reduce solid–solid contact electrification and TENGs output power, water droplets like rain droplets can specifically be used for charge formation, as also the liquid–solid contact creates significant surface charges and voltages (reaching kV from single droplets).^505,506^ This was exploited as an additional energy source.

Yet, for plant-based TENGs, some material considerations need to be made. Soft, flexible, transparent systems that do not harm the plants are required. At the same time, the systems should enable suitable mechanical excitation, for example, their dynamics in wind. If such properties are met, such plant-hybrid systems can provide energy sources for plant-wearable sensors or even systems that can deliver molecules to plants for precision agriculture, as recently shown in a proof-of-concept.^493^ Moreover, an advantage of using plant leaves directly as half-electrode of a TENG (typically in combination with materials like silicones, or FEP) is the intrinsic micronano-structures on the surfaces, e.g., by arrangement of epidermis cells and epicuticular waxes that create sites for effective charge generation. Indeed, such leaf surface structures are even replicated in artificial TENGs to improve power outputs.^444,445^

#### 7.5.3 Addressing Impacts on Organisms and the Environment

Despite these advantages, this technology faces a general challenge that goes beyond energy-harvesting itself, namely, how to install and maintain artificial components on a living organism over the long-term without adversely affecting it. The organism should remain fully functional during operation, which includes preventing mechanical damage from the applied materials rather than relying solely on the self-healing capacity of living tissues. For example, it was observed that coating all leaves of a plant on their upper (adaxial) side, which contains fewer stomata essential for transpiration, with silicone caused no evident effects on plant health even after one year.^499^ Here, in particular, soft materials and soft robotics provide tools for safer organism-device interaction. In the case of plants, systems should also be transparent to not hamper the photosynthetic activity of the leaf, and at the same time, systems must be lightweight.^507^

Moreover, the residues of such energy harvesting systems may have consequences for the direct environment and the entire ecosystem in which the system is applied. The correlation between such devices and ecosystems is not fully understood, although it is clear that toxic or polluting residues must be avoided. As a starting point, for outdoor applications, high-performance materials would be desired that are additionally biodegradable, that do not degrade during operation, but e.g., together with the leaf when it drops and naturally degrades. Such high-tech material systems with tailored degradability in the environment must still be developed. That this is possible show some in vivo examples of transient electronics and TENGs.^508,509^

#### 7.5.4 Simplicity as a Key Strategy

Moreover, simplicity represents a key design strategy. Recent studies have shown that even a single electrode can be sufficient to harvest electricity from raindrops impacting a leaf.^501^ It is also known that leaves become electrically charged upon contact with raindrops through liquid–solid contact electrification.^510^ Further research that combines simplicity with functional performance, while also improving the environmental friendliness of these systems, could enable their deployment in precision agriculture, environmental monitoring, and other plant-hybrid technologies.^511^ An example in this direction is the previously mentioned plant-hybrid wind energy harvester, which powers a molecular delivery system capable of administering precision treatments to plants (Figure 16i, right panel).^493^

**Figure 16:**
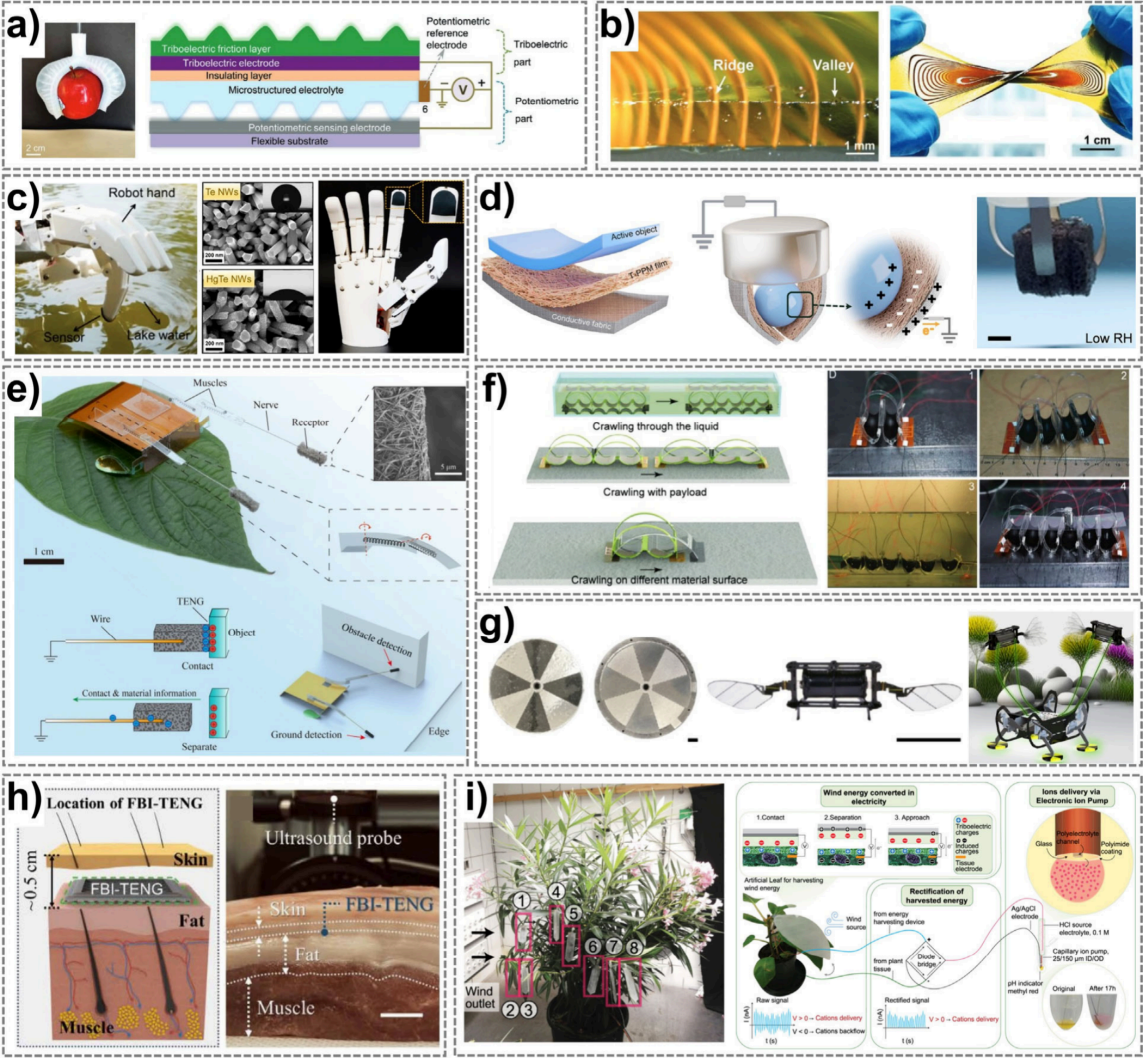
Examples of structures and applications of TENGs as sensors and energy harvesters in robotics and related applications. a) Potentiometric sensing combined with TENG sensing, the combination of TENGs with other sensing principles often enhances multimodality, reliability, and sensitivity of the sensor. Reprinted with permission from ref 461 Copyright 2020 John Wiley & Sons. b) Harnessing chemistry to obtain fingerprint-like structures of TENG sensors, exploiting Liesegang patterns to improve sensing capabilities. Reprinted with permission from ref 464 Copyright 2020 John Wiley & Sons. c) Chemical sensing by a TENG on a robotic finger using the variation of liquid–solid contact electrification of telluride nanowires after reacting with heavy metal ions. Reprinted with permission from ref 456 Copyright 2023 American Chemical Society. d) Combination of humidity-responsive actuation of a gripper and TENG sensing. Reprinted with permission from ref 465 Copyright 2023 American Chemical Society. e) Insect-inspired robot with extremely lightweight TENG sensors in the antennas. Reprinted from Nano Energy, Vol 114, Zhu et al., Self-powered bionic antenna based ontriboelectric nanogenerator for micro-robotic tactile sensing, Page 108644, Copyright 2023, with permission from Elsevier.^467^ f) A leech-inspired soft robot with segmented DEA muscles and triboelectric suckers for amphibious movement, climbing, and load-carrying, powered by an external tethered TENG. Reprinted with permission from ref 485 Copyright 2025 John Wiley & Sons. g) The central image shows a winged microaerial robot whose liftoff could be fully powered by a tethered rotary TENG whose structure is shown on the left. Reprinted from Nano Energy, Vol 126, Lee et al., Liftoff of a soft-actuated micro-aerial-robot powered by triboelectric nanogenerators, Page 109602, Copyright 2024, with permission from Elsevier.^486^ h) An ultrasound-driven transient, degradable TENG that can be powered through tissue. Reproduced from Lee et al., Science Advances, DOI: 10.1126/sciadv.abl8423 [2022], AAAS.^452^ i) Wind-driven plant hybrid TENGs that use the plant as part of the energy conversion to power wireless environmental sensors (left panel) and a molecular delivery system by wind (right panel). Reprinted with permission from refs 493,494 under CC BY 4.0, Copyright 2024 IOP Publishing.

### 7.6 Material Selection Rationale and Research Outlook

In the following, we focus on additional aspects of TENGs in soft robotics and related applications, which can be regarded as design tips and future research directions.

#### 7.6.1 Material Selection Rationale for TENGs

The triboelectric effect occurs on most materials as mentioned above, but some materials and material combinations perform better than others. We provide a practical guide for roboticists and non-materials scientists on how to select, optimize, and use materials in TENGs to convert ambient and environmental mechanical energy into electricity, rather than offering a comprehensive review of possible TENG materials and their specific properties, which has been covered elsewhere in multiple publications.

1). Materials: As mentioned throughout the section, a general rule of thumb for creating a TENG is to pair materials that tend to acquire net negative surface charges with materials that tend to acquire net positive charges, forming an effective contact pair. Electrodes are placed beneath these tribo-active materials to enable electrostatic induction and collect the charges generated during contact-separation. In most cases, the negatively charging materials are fluorinated polymers or silicones, while the positively charging counterparts include polyamides, polymethyl methacrylate (PMMA), polyurethane, or metals such as aluminum. Examples of more specific material combinations are listed in Table 9 and given in the qualitative and quantitative triboelectric series described earlier.^435–437^ For soft robotics, silicones like PDMS might be the first choice as negatively charging materials. Their performance can be enhanced through surface micro- and nanostructuring to increase the effective contact area, surface chemical modification (e.g., grafting fluorinated end groups) to improve charge affinity, and physical techniques such as ion etching to induce additional surface charges.^512^ Furthermore, optimizing the dielectric layer thickness while minimizing dielectric breakdown and charge leakage can significantly increase the achievable charge density.
2). Mechanics: The contact motion, determined by mechanical design, is a creative aspect but also a critical factor governing the performance of single- or multielectrode TENGs operating in vertical, rotational, or sliding modes. This motion directly affects the electrical output by influencing parameters such as contact area, separation distance, applied force, surface damage, and frequency.
3). Application scenarios: Where the TENG will be operated plays a crucial role (in structured or unstructured environments, in air, in water, etc.). As highlighted earlier, environmental factors like humidity, along with other factors such as material history and time-dependent changes like degradation, e.g., of delicate surface nanostructures, are critical considerations for reliable TENG operation. Surface wettability of the materials is important since the presence of surface-adsorbed water layers can significantly reduce charge generation (also in droplet-based TENGs).

Table 10 summarizes the key parameters that guide the option for materials. Often, more complex approaches may be necessary. More challenging is, for example, selecting tribo-active materials when they are required to perform multiple functions, for example, combining actuation strategies such as humidity-driven^465^ or magnetic actuation^492^ with TENG-based sensing. In these cases, the material choice is driven by multifunctionality and cannot be generalized.

**Table 10:** Summary of Crucial Design Parameters for Selecting and Designing Materials to Create TENGs for Powering Robotic/Soft Robotic Components, Sensors, and Applications

**Tribo-active negatively charging materials (examples)**	**Tribo-active positively charging materials (examples)**	**Surface treatments enhancing charge yields**	**Contact parameters affecting charging and yields**	**Environmental conditions affecting charging**	**TENG-powered soft robotic components and applications**
- Fluorinated polymers: FEP, PTFE, PVDF	- PA	- Micro- and nanostructuring	- Contact area	- Humidity and air dielectric properties	- Sensors: tactile, motion, expansion, bending, vibration, texture, (surface) chemistry (nature of contact material). Typical output: voltage mV to a few V, power n-μW cm^–2^
- Silicones: PDMS, EcoFlex, custom variations	- PU	- Chemical functionalization, e.g., fluorination and amination	- Separation distance and speed	- History of surfaces and degradation	- Actuation: powering DEA, electrohydrodynamic, IPMCs. Typical output: voltage kV range, power mW cm^–2^
- PI	- PMMA	- Reactive ion etching	- Contact force and pressure	- Surface contamination	
	- Skin	- Adjusting Young’s modulus to increase the contact area	- Contact frequency and contact-separation time		
	- Plant cuticle		- Surface wettability (especially for liquid–solid contacts)		

#### 7.6.2 Electrical Circuits and Power Management

How the power produced by TENGs is managed plays an additional crucial role. In many reported examples, one of the best options is a standard bridge rectifier as a simple passive component to convert the AC output of TENGs into DC. More specialized power management circuits can further influence and enhance the amount of harvestable charge^513,514^ as well as the intrinsic capacitance of the TENG.^515^ The performance of circuits and power management systems is often highly dependent on operating frequency and other application-specific factors. For sensing applications, TENGs typically produce electrical outputs ranging from millivolts to a few volts, with power levels in the nW to μW cm^–2^ range. In contrast, actuation tasks require higher-performance TENGs capable of delivering outputs in the kilovolt range and power densities on the order of mW cm^–2^. Consequently, whether high-performance tribo-active materials are needed depends on the specific application scenario. Because TENG output is highly sensitive to mechanical and environmental conditions, performance can vary substantially with the energy source used. Therefore, in most cases, the power output must be evaluated for each specific application and cannot be treated as a generalizable design parameter: it ultimately depends on where, how, and for how long the TENG is operated.

#### 7.6.3 Lab Testing Vs Real-World Conditions

Typical TENG performance is evaluated by measuring open-circuit voltage, short-circuit current, transferred charge, and output power under controlled mechanical actuation (defined force, frequency, and contact area), often complemented by durability cycling and environmental stability tests. These measurements, together with estimates of surface charge density, provide a basis for comparing materials and designs under reproducible conditions. The parameters used for testing TENG materials should closely reflect the conditions under which they will be applied. For instance, material performance is often evaluated using impact forces of several tens to hundreds of newtons, far higher than those typically generated by environmental energy sources driving the contact-separation motion. While efforts have been made to standardize TENG material characterization, such protocols are still often tightly controlled during lab tests to ensure reproducibility. Therefore, it is essential to also evaluate designed TENGs under their intended application conditions.

#### 7.6.4 Bioinspiration

TENGs are not inherently bioinspired technologies. However, bioinspired approaches enable improving TENGs^445^ and assist the development of soft-robot-compatible designs, such as skin-inspired and skin-like TENG implementations for tactile sensors (see skin-like TENG sensors in Table 9). Moreover, mimicking biological materials and their micro- and nanostructures, such as fish skin or the cuticle of superhydrophobic leaves, has been shown to improve TENG performance. For example, the voltage produced by a lotus-leaf microstructure-inspired TENG increased up to 5.8 times from ∼ 55 to 320 V.^444^ Further examples demonstrate that nature-inspired design approaches can enhance TENG performance,^445^ but the full potential of natural systems and materials to influence and inspire TENG designs remains largely unexplored. For instance, while leaf surface structures enhance charging, plant organs with higher lignin content, such as wood, reduce charging.^516^ This understanding has driven the development of antistatic additives for polymers, which are essential in industries where excessive charge buildup and electrostatic discharges can cause damage.^516^

#### 7.6.5 Biodegradable TENGs

Effort is also being made in the direction of creating biodegradable TENGs, using bioresorbable and biodegradable materials.^508,509,517^ Although these materials generally produce lower power output, they offer the added functionality of biodegradability, an option not feasible with the often-used high-performance fluorinated TENG materials, thereby expanding the range of possible applications. A focus is on achieving transient biomedical sensors, and power generators, but also environmentally degradable systems are extremely relevant. A fundamental step is finding alternatives to fluorinated polymers as triboelectric negative materials: for example, polylactic acid derivates have shown promising results^518^ but still have not achieved the charge densities of polymers with fluorinated groups.

#### 7.6.6 Liquid–Solid TENGs

The contact of liquids, particularly that of water and solid surfaces, has recently been increasingly used to harvest energy through liquid–solid TENGs.^454,519^ The voltage by single droplets can be quite remarkable and reach the kV range^506^ and charge densities of mC m^–2^. Liquid–solid TENGs have shown great potential as sensors of flow, leakage, and bubbles in tubes,^454,520^ which could also be interesting in the context of soft robotics. Moreover, systems that, for example, combine solid–solid and liquid–solid TENGs have a multisource energy harvesting capability, such as harvesting wind and rain signals, compensating at the same time the often observed decrease in power output of solid–solid TENGs of wet surfaces.^498,521,522^

#### 7.6.7 Summary and Future Directions

In the future, it will be necessary to test and, if necessary, adapt TENGs in (soft) robotic application-specific scenarios that analyze the effect of a dynamic, changing environment and modify the structure of the TENG, either artificial or biohybrid, and tailor it to the actually occurring environment and energy source in a way that reliable long-term performances are possible. Possible strategies are possible through material design, theoretical approaches, especially mechanical design, and adaptation, for example, to living organisms,^507^ to extend the application range and successfully harvest biomechanical energy. Moreover, further integration of TENGs in robotics and especially soft robotics is required through the as mentioned engineering and testing of TENGs under relevant conditions as well as through making use of the multifunctionality of material properties that allow actuation, sensing, and power generation, to achieve reliable physical intelligence in materials and intelligent matter capable of leveraging the environment as a power source and widening the functions of robots.^523^

## 8 Wind-Dispersed Fliers

In this section, we examine wind-dispersed fliers and clarify their inclusion within the scope of soft robotics adopted in this review. Wind-dispersed fliers are attractive interests in energetically sustainable soft robotics because they enable autonomous deployment and motion by directly coupling structural compliance and geometry design with environmental airflow, without onboard power or centralized control.

Unlike many soft robotic systems based on intrinsically soft or stretchable materials, many wind-dispersed fliers can be fabricated from conventionally rigid materials. Their effective compliance arises primarily from geometric and structural design, such as thin and high aspect-ratio films, porous structures, and fibrous architectures, rather than from low intrinsic material modulus. In this context, soft robotic behavior can also be functionally defined by distributed compliance, passive or stimulus-responsive deformation, and embodied physical intelligence, rather than by material softness alone.^2,524,525^ Under this definition, wind-dispersed fliers represent a geometry-enabled extension of soft robotics.

Within this functional definition, wind-driven seed and fruit dispersal mechanisms provide concise bioinspired design principles for such fliers (Figure 17). Winged seeds/fruits have thin (<0.2–0.4 mm) wings that maximize air resistance while minimizing weight, exhibiting diverse shapes and sizes across species.^526–528^ One example is samaras, which exploit mass distribution and asymmetric wing geometry to achieve stable autorotation and prolonged descent, illustrating how aerodynamic control can be embedded in structure alone.^528–530^ On the other hand, parachute-like fruits, such as dandelions, use a pappus of plumes arranged to maximize drag and facilitate horizontal dispersal.^531,532^ They have higher drag coefficients at low Reynolds numbers due to the circular pappus geometry and bristle connections that stabilize a separated vortex ring (SVR),^531^ while exhibiting humidity-responsive shape changes that modulate dispersal behavior. These examples highlight how morphology can simultaneously encode sensing, actuation, and control through passive interaction with environmental flows.

**Figure 17:**
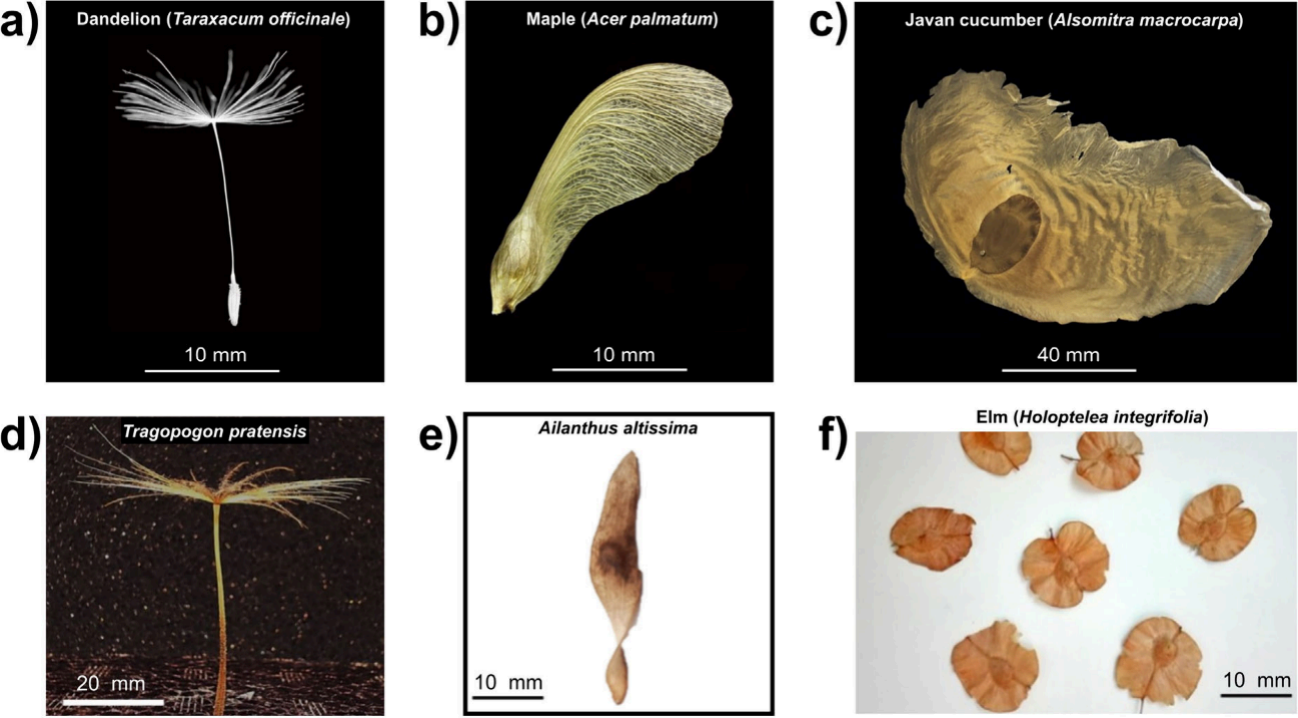
Natural seeds/fruits as inspiration for the development of artificial fliers. a) Dandelion (*Taraxacum officinale*) fruit. b) Maple (*Acer palmatum*) samara. c) Javan cucumber (*Alsomitra macrocarpa*) fruit. a–c) Reprinted from Current Biology, Vol 32/5, Viola and Nakayama, Flying seeds, Page R204, Copyright 2022, with permission from Elsevier.^538^ d) *Tragopogon pratensis* seed. Reprinted with permission from ref 534 under CC BY 4.0, Copyright 2024 The Author(s). e) *Ailanthus altissima* seed. Reprinted with permission from ref 526 Copyright 2023, The Author(s). f) Elm (*Holoptelea integrifolia*) fruits. (Image source: Adityamadhav83, “Seeds of Indian Elm (*Holoptelea integrifolia*) collected from Bolarum Bazar, Hyderabad”, 2018. Accessed via CC BY-SA 4.0.

Within this framework, purely passive fliers represent a borderline case of structural autonomy, whereas environmentally actuated fliers incorporating hygroscopic or photothermal elements establish a clear sensing–actuation loop mediated by environmental stimuli. These wind-dispersed systems have attracted growing interest for applications such as aerial seeding and distributed environmental monitoring, where autonomous deployment and scalability are critical.^526,527,533–537^ The following subsections review the materials, structural designs, and representative wind-dispersed flier systems, with particular emphasis on sustainable options for large-scale dispersion.

### 8.1 Materials for Wind-Dispersed Fliers

The materials used in wind-dispersed fliers are primarily selected to achieve light self-weight, structural integrity, and durability, rather than intrinsic softness. In contrast to classical soft robotic systems based on elastomers or hydrogels,^2^ the fliers reviewed in this section are typically fabricated from conventionally rigid polymeric materials such as PI, PLA, PE, or layered polymer composite films. These materials generally exhibit elastic moduli in the gigapascal range, reflecting their intrinsically nonsoft mechanical nature.

Despite this intrinsic rigidity, wind-dispersed fliers achieve effective mechanical compliance through geometric and structural design. By exploiting high aspect ratios, thin membranes, porous microstructures, and hierarchical architectures, these systems exhibit sufficiently low bending stiffness to undergo large, continuous deformations under aerodynamic loading. As a result, their macroscopic mechanical behavior is compliant and continuum-like, enabling passive shape adaptation and safe interaction with the environment independent of the intrinsic material modulus. This geometry-enabled softness is well established in related fields such as origami- and kirigami-inspired robotics, where structure or pattern design is used to bridge the gap between rigid materials and soft robotic behavior.^539,540^

#### 8.1.1 Electronic Materials

Historically, the first class of materials used for seed/fruit-inspired fliers was based on electronic materials, including conductors like metals, semiconductors in functional components, substrates like printed circuit boards (PCBs), and photonic materials in LEDs. Among all, flexible PCB materials such as PI and conductive metal traces are the most used materials for fabricating the structural components of wind-dispersed, artificial, electronic fliers. PI is a high-temperature resistant polymer with outstanding mechanical properties (Young’s modulus of 1.5–3.0 GPa and tensile strengths of 70–100 MPa),^541^ dielectric properties, and chemical inertness, making them highly suitable for flexible PCBs where etching and soldering are required. For artificial fliers, PI was chosen because of its high specific strength, flexibility, and toughness.^542^ Examples of PI being used in artificial fliers include *Acer* samara-inspired fliers using PI with etched copper plating,^542–544^ dandelion fruit-like fliers using laser cut PI with integrated solar cells and electronics,^545^ and electronic fliers using encapsulated silicon nanomembrane nMOS transistors in a PI film on top of an epoxy-based SMP layer.^536^

#### 8.1.2 (Bio)­degradable Materials

In the last 5 years, a big effort was made toward a more sustainable solution by using biocompatible and/or biodegradable polymers, considering potential dispersal of a large amount of fliers in the environment. Biocompatible materials do not cause harm to living organisms when administered or absorbed at certain specific amounts,^546^ and biodegradable materials can be decomposed by microorganisms under aerobic and/or anaerobic conditions.^546^ Since the wind dispersal properties of passive and artificial fliers mainly depend on the geometry and aerodynamic designs rather than the intrinsic material properties, many widely available, easily processable materials can be chosen as the structural material for the fliers.

PLA, an aliphatic polyester usually made with α-hydroxy acids and derived from renewable resources such as corn starch,^546^ was used as the structural material for 3D printing fliers, such as these inspired by *Acer* samaras.^527,547^ PLA is one of the most used bioplastics in the 3D printing industry, due to its excellent processability, in the form of filaments for filament extrusion 3D printers. The mechanical properties of PLA include a Young’s modulus ranging from 1–4 GPa and an ultimate strain of 4%–7%. Biodegradation of PLA mainly depends on temperature (typically 55–60 °C) and available enzymes in the soil, and involves hydrolysis of the ester bond.^548^ The reported degradation in landfill/compost/soil ranges from 20–1000 μm/year.^549^ However, the hydrolytic degradation can be paired and sped up by thermal degradation, photodegradation, and microbial degradation.^550^ PLA has a low oral toxicity and can be considered environmentally safe: oral LD_50_ in rats is 5000 mg/kg.^551^ In addition to neat PLA filaments, blending 10 wt% of lanthanide fluorescent microparticles into PLA through a corotating twin-screw extruder equipped with a 2 mm diameter nozzle can achieve sensorized PLA filaments for the 3D printing of air-dispersed temperature sensors.^546^

Poly­(lactic-*co*-glycolic acid) (PLGA) is a copolymer of PLA and polyglycolic acid (PGA), and it is also biocompatible and biodegradable.^552^ Compared to PLA, PLGA is more amorphous, with reduced Young’s modulus as the glycolic acid ratio increases. PLGA biodegrades faster than PLA, by hydrolysis of its ester linkages into its constituent monomers, lactic acid and glycolic acid, through bulk or heterogeneous erosion, in aqueous environments.^512^ As an example of application in fliers, it has been used to fabricate biodegradable 3D micro, meso and macro fliers.^533^

Potato starch is an edible and biodegradable material that consists of tightly packed amylose and amylopectin polysaccharides.^553^ It is commercially available as wafer paper, made of potato starch, vegetable oil, and water. Thanks to its thin thickness and processability, it can be used as the wing for biodegradable artificial fliers. For instance, potato starch wafer paper was used for the fabrication of a glider inspired by *Alsomitra macrocarpa*, through cutting and molding.^537^

Cellulose acetate (CA) is a linear polysaccharide that refers to any acetate ester of cellulose, which is chemically modified from cellulose.^554^ Depending on the degree of substitution (DS), CA gains thermoplastic processability while remaining partially biodegradable: more hydrophilic and biodegradable for lower DS (<1); simpler dissolution and slower biodegradation for higher DS (>2.5). CA (DS 2–2.5) is insoluble in water but soluble in organic solvents such as acetone, DMSO, and DMF until 30% w/w, and it has a Young modulus of 1.5 GPa.^555^ For the biodegradation of CA, the first step is the hydrolysis of the acetyl groups, which requires the action of microorganisms with esterase enzymes, while the cellulose backbone is biodegraded by organisms through cellulase enzymes.^556^ CA fibers are biodegradable in moist soils, and they deteriorate after 2 months and are destroyed after 4–9 months.^556^ Toxicity experiments in rats revealed that after a maximum oral administration of 5000 mg/kg body weight/day for 94–96 days, no evidence of an adverse effect was recorded.^557^ For its usage in artificial fliers, researchers developed a formulation to fabricate porous and lightweight CA structures: dissolving CA in acetone at 30% w/w and mixing it with alkaline lignin powder or Na_2_CO_3_ crystals at various concentrations, to form a printing ink for DIW.^526,534^ After DIW, the printed fliers underwent a leaching process in water to remove lignin or Na_2_CO_3_, resulting in a porous CA structure. This process has been applied to fabricate fliers inspired by *Ailanthus altissima* and *Tragopogon pratensis*.^526,534^

#### 8.1.3 Stimuli-Responsive Materials

To achieve robotic fliers with actively controlled flight performance, other materials like stimuli-responsive materials are also employed. For example, thermo-responsive LCEs (Section 2.1.2) can be combined with photothermal materials and be used in fliers to introduce light-modulated shape morphing and flight behavior change. Specifically, an LCE mixture for light-triggered morphing fliers consisted of 0.3 mmol RM82, 0.115 mmol 6-amino-1-octanol, 0.115 mmol dodecylamine, and 2.5 wt.% 2,2-dimethoxy-2-phenylacetophenone (Irgacure 651), which was polymerized with UV light, exhibiting a Young’s modulus of 20 MPa at room temperature.^558^ Similar to LCEs, liquid crystal networks (LCNs) are cross-linked polymer networks of liquid crystal units, but with higher cross-link density and more glassy behavior. Photochemical LCNs, such as the one containing 52 mol% 4-methoxybenzoic acid 4-(6-acryloyloxyhexyloxy)­phenyl ester, 18 mol% 4­[4­(6-acryloxyhex-1-yl)­oxyphenyl]­carboxybenzonitrile, 21 mol% diacrylate cross-linker 1,4-bis-[4-(6-acryloyloxyhexyloxy)­benzoyloxy]-2-methylbenzene, 6 mol% 4,4’-bis­[9-(acryloyloxy) nonyloxy]­azobenzene and 1.5 mol% of photoinitiator, can be used for light reactive films in the development of fruits-inspired fliers to implement angle variation of their wings or pappus.^559^

Another way to induce deformation through external excitations is to build bilayers with stimuli-responsive materials, such as photothermal and hygroscopic materials. Specifically, photothermal gold nanorods were combined with a PI/LDPE bilayer film to develop a parachute flier that could open and close the pappus,^560^ and MXene/PE bilayer actuators were integrated into shape-morphing fliers with multiresponsiveness, including humidity, IR, temperature, volatile organic compounds (VOCs), and voltage.^561^

### 8.2 Material Selection Rationale

Lightweight polymers are ideal starting points for fruits/seeds-inspired fliers thanks to their ease of 3D printing and shaping into thin wings or aerodynamic structures. Ultralight thin films based on polymers such as PLGA, CA, or PI can serve as aerodynamic surfaces after stretching, effectively mimicking the thin structures found in natural wind-dispersed fruits/seeds. At the same time, additive manufacturing of thermoplastic materials like PLA, PCL, or CA enables the creation of lattice structures that introduce controlled porosity, significantly reducing mass while maintaining the necessary aerodynamic surface area and mechanical stability. In addition, to ensure ecological sustainability, especially for dispersal at large scales, naturally biodegradable materials (e.g., CNCs, PHA, PCL, etc.) are highly valuable. This is particularly relevant in the case of artificial fliers, which are typically designed to be dispersed into the environment for environmental monitoring purposes and not to be retrieved. Beyond passive dispersal, there is also growing potential in employing stimuli-responsive materials, enabling adaptive flight behaviors or externally controlled modifications for robotic fliers. However, many existing responsive systems rely on nondegradable polymers (e.g., LCEs, LCNs), creating a sustainability challenge. Therefore, using biodegradable alternatives for thermal- or light-responsive systems, such as bioderived photothermal materials, biodegradable SMPs and bilayers, represents an important direction for environmentally responsible flier design.

### 8.3 Wind-Dispersed Artificial Fliers

Artificial structures bioinspired by flying fruits/seeds can be dispersed by unmanned aerial vehicles (UAVs) over targeted areas using wind as the dispersing agent. Building wireless sensor networks for gathering environmental data, in the framework of the Internet of Things (IoT), is one of the applications to tackle the climate change crisis.^562^ For this purpose, single-winged samaras were used as a bioinspiration source to build self-deployable carriers integrated with electronic sensors.^543,542,544^ Low-cost and lightweight sensors for fire detection embedded in a PCB were incorporated in artificial samara fliers.^543,542,544^ Researchers needed an effective strategy for soft landing of the fire sensor, so they took advantage of the passive flight of samaras, which allows them to achieve a low terminal velocity and a safe impact on the ground.^543,542,544^ The same concept was exploited for the development of sensors for in situ monitoring of atmospheric parameters.^563,564^ Carrier fliers, inspired by samaras and parachute fruits/seeds for achieving low descent speeds, were equipped with sensors for air temperature, air pressure, relative humidity, and wind speeds, supplied with a global positioning system (GPS) for coordinates and timestamps.^563,564^ In the work of Iyer et al., the battery power supply, the sensing circuit for temperature, humidity, and ambient light, were substituted with solar cells and an energy harvesting circuit.^545^ Bioinspiration of their flying system was drawn by the parachute fruit of the dandelion and by the elm fruit.^545^ This choice was made to exploit the low descent speed typical of parachute fruits and to achieve an upright landing, fundamental for solar harvesting. This resulted in a device that could descend at a speed of 0.87 m s^–1^, travel for 50–100 m in low wind conditions, and land in an upright position 95% of the time. It was also shown that the flight dynamics of the artificial flier could be modulated by varying the porosity and diameter of the artificial pappus, demonstrating that a certain degree of control on the travel distance could be achieved. The sensorized flier could wirelessly measure temperature, humidity, light, pressure, magnetic fields, and acceleration. Yet, these solutions all rely on electronics, which, even though provides market ready sensors and accurate measurements, if not properly disposed, can lead to e-waste that cannot be decomposed and often contains heavily toxic materials.^565^

An active effort toward biodegradable fliers for environmental sensing was provided by Rogers’s group.^533,535,536^ They first developed bioinspired fliers made of PI and SMP of different size scales, from macro to micro, embedded with electronic and colorimetric sensors.^536^ The design of the fliers was derived from*Tristellateia australasiae* fruit, a multi-winged samara that has an autorotational vertical flight. Analytical, computational, and experimental studies of aerodynamics were made to optimize the aerodynamic behavior of the bioinspired structures. Yoon et al. introduced biodegradable materials (PLGA and cellulose) for the fliers, and incorporated colorimetric sensors for pH, heavy metal concentrations, and UV exposure, alongside humidity and temperature (Figure 18a).^533^ Design of the fliers was also inspired by *Tristellateia australasiae*, but different geometries for achieving parachute flight were explored, tested, and characterized. Field tests were made to demonstrate the deployment by drones of these fliers and the colorimetric reading of the various parameters via digital image capture. In addition, Kim et al. developed a hybrid flier system, fabricated through a process of controlled buckling of PLGA.^535^ These structures were inspired by samaras and parachute seeds and could generate the same aerodynamic outputs as the natural systems, which are the leading edge vortex (LEV) and SVR, respectively.^535^ The aerodynamics of these fliers was optimized by theoretical, computational, and experimental approaches. The proposed fliers could carry various payloads such as bioresorbable, colorimetric gas sensors and LEDs, for diverse scenarios in remote sensing.

**Figure 18:**
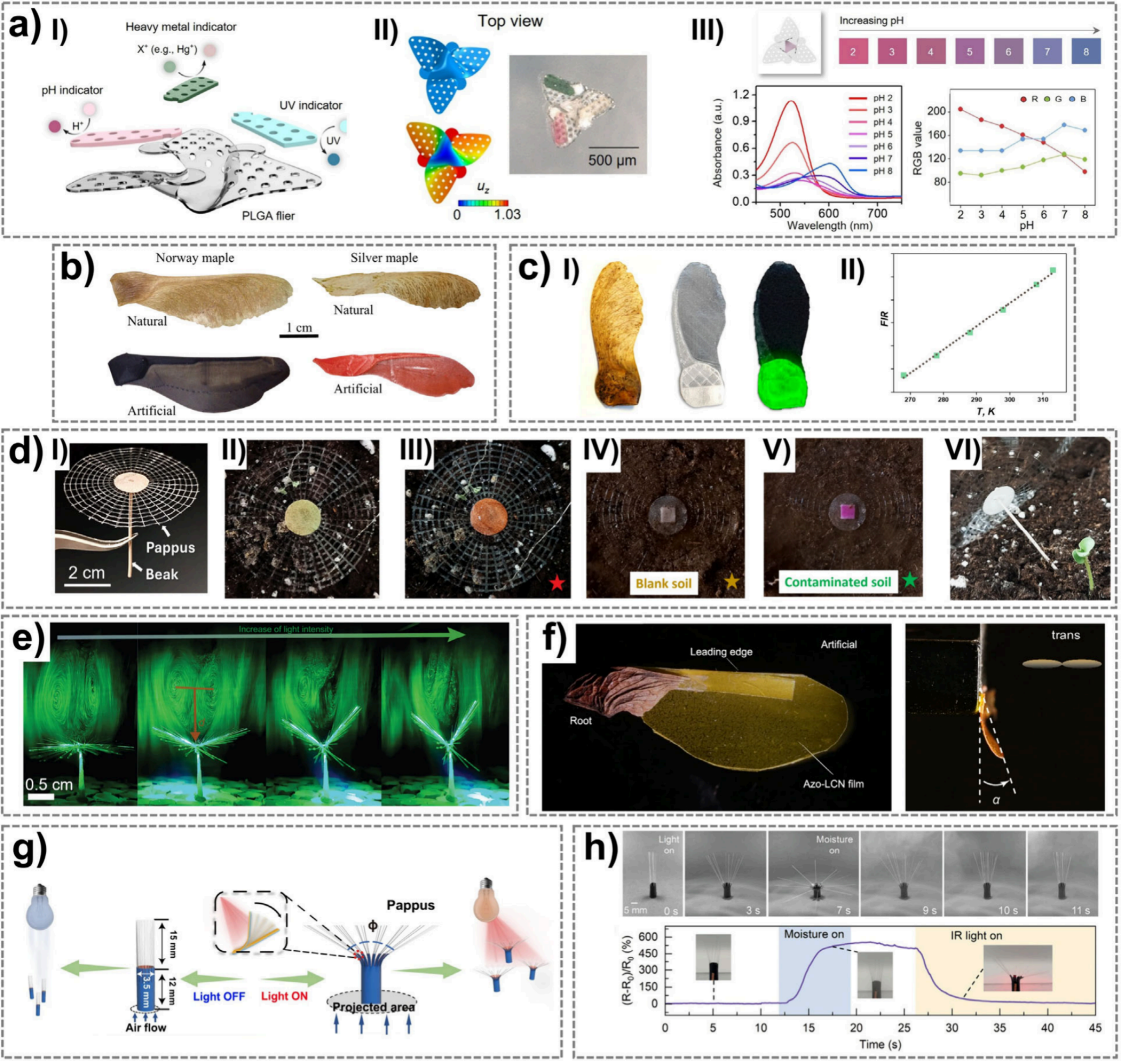
Bioinspired, wind-dispersed artificial fliers. a) Biodegradable *Tristellateia* flier for colorimetric sensing: I) schematic illustration of the device with a PLGA body and colorimetric assays of chemical indicators supported by cellulose; II) top view optical micrographs of a 3D colorimetric microflier with corresponding geometries predicted by finite element analysis; III) pH-dependent color changes between pH 2 and pH 8, analyzed from UV–visible spectroscopy and RGB analysis of digital images. Reproduced from Yoon et al., Science Advances, DOI: 10.1126/sciadv.ade3201 [2022], AAAS.^533^ b) Natural and 3D printed versions of both *Acer platanoides* and *Acer saccharinum*. Reprinted with permission from ref 547 under CC BY 4.0, Copyright 2021 MDPI. c) 3D printed *Acer* for luminescent sensing: I) *Acer campestre*: natural (left), fluorescent under daylight (middle), and fluorescent under NIR laser in the dark (right); II) measured FIR as a function of temperature of the fluorescent flier. Reproduced from Cikalleshi et al., Science Advances, DOI: 10.1126/sciadv.adi8492 [2024], AAAS.^527^ d) 3D printed *Tragopogon* for environmental monitoring: I) picture of the artificial *Tragopogon*; II) pH indicator integrated with the artificial pappus on the soil; III) pH indicator color change after dropping an acid solution (pH = 4.2); IV, V) nitrate indicator integrated with the artificial pappus on noncontaminated soil (IV) and nitrate-contaminated soil (V); VI) growth of a mustard plant after 6 days of incubation, from aerial seeding of an artificial flier coupled with a mustard seed. Reprinted with permission from ref 534 under CC BY 4.0, Copyright 2024 The Author(s). e) Light-driven dandelion-inspired microfliers. Photos of the vortex ring pattern change and angle change upon light illumination (left to right, 100, 150, 200, 300 mW cm^–2^) on an artificial flier. Reprinted with permission from ref 558 under CC BY 4.0, Copyright 2023 The Authors. f) Photochemically responsive polymer films enable tunable gliding flights. Reprinted with permission from ref 559 under CC BY 4.0, Copyright 2024 The Author(s). g) Light-driven dandelion-inspired microfliers with light-controlled drag force and falling velocity. Reprinted with permission from ref 560 under CC BY 4.0, Copyright 2023 The Author(s). h) Multistimuli responsive dandelion-inspired flier, with snapshots of the rapid opening and closing process of the flier in response to IR stimulus and subsequent moisture exposure. Reprinted with permission from ref 561 under CC BY 4.0, Copyright 2023 John Wiley and Sons.

The importance of materials selection in the making of artificial fliers is evident in light of tackling climate change. Soft robots and fliers systems should go in the direction of zero-footprint, biodegradable eco-robotics, and combine structural and functional capabilities, such as sensing, self-healing, and self-terminating.^565^ Transient fliers made from fully biodegradable and non-fossil-based materials that do not turn into hazardous e-waste at the end of their life cycle would constitute an ideal solution for environmental sensing applications.^537^ In this direction, Wiesemuller et al. showed the design and fabrication of a gliding flier inspired by *Alsomitra macrocarpa*, made of potato starch paper and coupled with a gelatin/cellulose-based hygroscopic actuator that opens when it rains to expose a pH paper sensor for colorimetric reading of the rain pH.^537^

The feasibility of using 3D printing as an effective method of fabrication of fliers was shown by Nave et al.^547^ They drew inspiration from *Acer platanoides* and *Acer saccharinum* to design and 3D print their artificial counterparts using PLA, using a biomimetic approach (Figure 18b). They characterized the morphology and aerodynamics of natural and artificial *Acer* fruits and did field experiments of dispersal, demonstrating the effectiveness of such solutions for perspective applications in environmental monitoring. As one step further, in the work of Mazzolai’s group, an air-dispersed sensor inspired by *Acer* samaras, was 3D printed in a single step, by using a PLA filament mixed with fluorescent microparticles (Figure 18c, panel I).^527^ Aerodynamic properties similar to those of the natural samaras were achieved, especially the same descent speed (∼ 1 m s^–1^), and dispersion tests were made by releasing the flier from a drone at 3 m to investigate the travel distance at a wind speed of 1 m s^–1^.^527^ The fluorescent emission (fluorescence intensity ratio, FIR), triggered by a laser, was calibrated with the application of measuring the temperature of topsoil (Figure 18c, panel II). In order to further decrease the environmental impact, a biodegradable and porous material was developed based on CA, that could be used for 3D printing of wind-dispersed systems.^526^ First, a flier inspired by *Ailanthus altissima* was developed,^526^ which had the characteristic of tumbling flight, meaning that it could travel even in the absence of wind.^566^ It was then coupled with a CNC photonic crystal for colorimetric measuring of relative humidity.^526^ Using the same porous material, a biodegradable flier inspired by the parachute fruit of *Tragopogon pratensis* was developed (Figure 18d), which provided a larger area on the pappus to accommodate colorimetric sensors for pH (Figure 18d, panels II and III) and nitrates (Figure 18d, panels IV and V).^534^ The flier was also tested as a carrier for mustard seeds to investigate perspective aerial seeding applications (Figure 18d, panel VI).

Other external energy sources have been explored in combination with wind to enhance the flight control of bioinspired artificial systems, effectively transforming passive fliers into actively controlled aerial robots. Modulating the spatial configuration of the flier can alter both descent speed and flight mode. For example, adjusting the angle of samara-inspired wings or the orientation of the pappus in parachute fliers offers a means of dynamic control. The possibility of using light-responsive polymers to build light-modulated fliers was demonstrated recently.^558–561^ In this direction, Zeng’s group reported the development of a flier, inspired by the dandelion fruit and replicating its aerodynamic behaviors, highlighted by the presence of SVR (Figure 18e).^558^ The actuator was made of an LCE, which could morph under visible light irradiation by closing and opening the parachute pappus.^558^ A closed pappus led to a faster diving descent in contrast to that of the slow parachuting in the open configuration, as demonstrated by aerodynamic studies of parachute fruits.^532,567^ Light-driven control of takeoff, flight, and landing constituted an additional level of maneuverability, beyond simply using wind.^558^ In a later work, they used photoresponsive azobenzene LCN to build single-winged soft robots/fliers inspired by *Acer* samaras, which could reversibly morph their shape under irradiation of UV and visible light (Figure 18f).^559^ Aerial dispersion could be modulated by light both in indoor and outdoor environments. The scalability of the light-guided fliers was investigated, and other geometries, inspired by *Alsomitra macrocarpa* and parachutes, were explored.

In another work by Chen et al., light-driven microfliers, also inspired by dandelion fruits, were fabricated by using bilayer film actuators made of PI and LDPE with embedded photothermal gold nanorods (Figure 18g).^560^ The flight achieved was very similar to that of dandelion fruits, showing the formation of SVR. The flight behavior could be modulated in midair by light through changing the pappus angle.^560^ A similar dandelion-inspired soft robot/flier was developed by employing a bilayer MXene/PE actuator, which was sensitive to humidity, temperature, applied voltage, IR light, and selective VOCs.^561^ In addition, the fliers showed self-sensing, as the resistance of the MXene layer changed with variations of humidity and IR light (Figure 18h).^561^ Table 11 summarizes the state of the art of the wind-dispersed fully passive and stimuli-responsive artificial fliers discussed in this section, with emphasis on the intrinsic modulus and geometry design of their constituent materials, flier size/mass, fabrication process, aerodynamic performance, functions, energetic sustainability classification, and environmental impact. When considering the energetic sustainability classification, those fliers requiring electronics or external inputs to enable sensing or morphing functions are considered as hybrid-driven systems, while all other passive systems are fully sustainable.

**Table 11:** Summary of Wind-Dispersed Passive and Stimuli-Responsive Fliers

**Bioinspiration**	**Materials, geometry design and fabrication process**	**Aerodynamic performance**	**Functions**	**Energetic sustainability classification**	**Environmental impact**	**Ref**
**Passive fliers**						
Samara (*Acer nigrum*)	A thin (150–600 μm), wing-shaped flexible PCB made of PI (E ≈ 1.5–3 GPa).^541^ Length: 12 cm; mass: 15 g.	Descent speed: 0.65 m s^–1^; rotation: 110 rpm.	Monitoring: temperature, humidity, pressure, bushfire.	Hybrid-driven	Not biodegradable	^542–544^
	Fabrication: hot forging and etching of PCB.		Electronic wireless communication through an antenna.			
Samara (*Acer platanoides)*	Samara-shaped thin (50 μm), flexible wing made of PLA (E ≈ 1–4 GPa).^548^ Length: 5 cm; mass: 183.6 mg.	Descent speed: 1.28 m s^–1^.	None	Fully sustainable	Nontoxic, partially biodegradable	^547^
	Fabrication: 3D printing.					
Dandelion (*Taraxacum officinale)*	Dandelion-shaped thin (7.5–25 μm), porous, flexible pappus made of PI (E ≈ 1.5–3 GPa)^541^ and metals for electronics. Diameter: 10–50 mm; mass: 20–70 mg.	Descent speed: 0.3–2.3 m s^–1^.	Monitoring: temperature, humidity, light, pressure, magnetic fields, and acceleration.	Hybrid-driven	Not biodegradable	^545^
	Fabrication: laser cutting, post-integration of electronics.		Wireless electronics with an antenna and solar cells.			
*Tristellateia australasiae*	Aerodynamic surfaces made of thin (∼5 μm), soft SMP (E = 2 MPa) layer and thin (∼3 μm), flexible PI (E ≈ 1.5–3 GPa)^541^ layers.	Descent speed: 0.2 m s^–1^ for mesoflier of 7.6 μg.	Monitoring: fine dust pollution.	Hybrid-driven	Not biodegradable	^536^
	Diameter: 40 mm (macroflier); 2 mm (mesoflier); 0.4 mm microflier. Mass: < 180 mg (macroflier); 5–20 μg (mesoflier); 0.3–1.3 μg (microflier).					
	Fabrication: etching and conventional electronic integration.					
*Tristellateia australasiae*	Aerodynamic surfaces made of a thin (∼60 μm), flexible PLGA (E = 1.37 GPa) layer and a thin (∼80 μm), flexible cellulose (E = 340 MPa) layer.	Descent speed: 0.7–1 m s^–1^.	Monitoring: humidity, pH, light exposure, and heavy metals.	Fully sustainable	Biodegradable	^533^
	Diameter: ∼ 4.2 mm; mass: 1.5 mg.					
	Fabrication: laser ablation, bonding, 3D shaping via heating, dyes integration via vacuum filtration on the cellulose layer.					
*Alsomitra macrocarpa*	Alsomitra-shaped wings made of potato wafer paper (E ≈ 150–200 MPa,^568^ thickness not reported). Sensing/actuation component made of a bilayer of thin (30 μm) CNF/gelatin (E = 3.2–14.6 GPa) layer and an ultrathin (1.5 μm) shellac layer.	Gliding speed of the natural seed 0.3–0.7m s^–1^.	Monitoring: pH.	Fully sustainable	Biodegradable	^537^
	Mass: 1.25–1.75 g; maximum width 140 mm.					
	Fabrication: plotter cutter, pressing into a 3D shape.					
Samara (*Acer campestre*)	Samara-shaped thin (50 μm), flexible wing made of PLA (E ≈ 1–4 GPa)^548^ and incorporated lanthanide microparticles. Mass: 55 mg; flier length: 29 mm.	Descent speed: 1.04 m s^–1^.	Monitoring: temperature.	Fully sustainable	Nontoxic, partially biodegradable	^527^
	Fabrication: 3D printing.					
Samara (*Ailanthus altissima*)	Samara-shaped thin (200 μm), porous wing made of CA (E = 1.5 GPa)^555^ and lignin (leaching material). Mass: 22 mg; length: 49 mm.	Descent speed: 0.64 m s^–1^.	Monitoring: humidity.	Fully sustainable	Biodegradable	^526^
	Fabrication: DIW printing and leaching.					
Parachute and autorotating seeds	Thin (∼60 μm), flexible parachute made of PLGA (E = 1.37 GPa).^533^ Size from cm (macro) to μm (micro).	None	Monitoring: temperature, humidity, pH, UV, gas.	Fully sustainable	Biodegradable for the parachute	^535^
	Fabrication: buckling for 3D shape.					
Parachute (*Tragopogon pratensis*)	Thin (∼20 μm), porous, flexible parachute made of CA (E = 1.5 GPa)^555^ and lignin (leaching material). Mass: 53.8 mg; length: 44.5 mm.	Descent speed: 0.65 m s^–1^	Monitoring: pH and nitrate.	Fully sustainable	Biodegradable	^534^
	Fabrication: DIW printing and leaching.					
**Stimuli-responsive fliers**						
Dandelion (*Taraxacum officinale)*	Flexible and fibrous pappus made of a thin (5 μm), soft LCE film (E = 20 MPa) and fabric filaments.	Descent speed: 0.46–0.66 m s^–1^.	Morphing: bending of the pappus upon visible light illumination.	Hybrid-driven	Not biodegradable	^558^
	Diameter: 1.5–2 cm; mass: ∼ 0.73 mg.					
	Fabrication: trimming, UV curing.					
Dandelion (*Taraxacum officinale)*	Flexible and fibrous pappus made of a bilayer consisting of a 8 μm thick PI (E ≈ 1.5–3 GPa)^541^ layer and a 5 μm thick LDPE (E ≈ 0.14–0.3 GPa)^569^ layer, with embedded photothermal gold nanorods.	Descent speed: 0.41–0.98 m s^–1^.	Morphing: bending of the pappus upon NIR light illumination.	Hybrid-driven	Not biodegradable	^560^
	Flier mass: 4 mg.					
	Fabrication: cutting and sticking.					
Samara (*Acer genus*)	Samara-shaped wing made of 20 μm thick azobenzene LCN (E ≈ hundreds of MPa).^570^	Descent speed: 0.98 m s^–1^.	Morphing: bending of the wing upon UV/vis light illumination.	Hybrid-driven	Not biodegradable	^559^
	LCN wing size: 2.5 cm; mass: 33.1 mg.					
	Fabrication: cutting and sticking.					
Dandelion (*Taraxacum officinale)*	Flexible and fibrous pappus made of a bilayer consisting of a 4.25 μm thick MXene (E ≈ 28 to 72 GPa)^292,293^ layer and a 22 μm thick PE (E ≈ 0.14–0.3 GPa)^569^ layer; glass fibers as the extensions on the fibrous pappus.	Descent speed: 0.63 m s^–1^.	Morphing: bending of the pappus upon IR light, humidity, temperature, applied voltage, and selective VOCs.	Hybrid-driven	Not biodegradable	^561^
	Flier diameters: 970 μm, 2.5 mm, and 9 mm					
	Fabrication: cutting and sticking.					

In summary, artificial fliers bioinspired by seed or fruit dispersal mechanisms offer a promising approach for environmental monitoring, precision agriculture, and ecosystem preservation. The ability to passively ride air currents with minimal energy consumption makes them particularly attractive for large-scale deployment in remote or inaccessible areas. However, many challenges, such as limited flight control, inconsistent dispersal patterns, and short operational lifespans, must be addressed to fully harness their potential.

Future advancements in bioinspired artificial fliers will likely focus on improving aerodynamic efficiency, advancing biodegradable and stimuli-responsive materials, and integrating lightweight sensing and communication systems. Additionally, incorporating artificial intelligence, swarm coordination, and energy-harvesting mechanisms may enable adaptive flight behaviors, autonomous data collection, and self-sustaining sensor networks for real-time environmental assessment.

## 9 Conclusions, Open Challenges, and Future Directions

In this review, we surveyed the materials and strategies enabling soft robots powered by diverse sustainable energy sources abundantly available in nature, including heat, humidity, sunlight, osmotic gradients, pH, triboelectricity, and wind. Across all categories, the integration of responsive and multifunctional materials is key to embedding physical intelligence in soft robots for passive and autonomous adaptivity to natural stimuli,^24^ or harvesting, converting, and storing usable energy for untethered, continuous robotic functions. The following paragraphs summarize the stimuli-responsive and multifunctional materials for each energy source, with a brief discussion about their performance, suitable applications, and current limitations.

*Thermo-responsive materials* enable soft robots to harness natural temperature variations for actuation, with one-way SMAs and SMPs particularly suited for deployable structures due to their large deformation and force output. Many formulations can operate within the typical environmental range (−20–60 °C), making autonomous deployment and shape programming feasible in outdoor environments or in the human body. For reversible motions, two-way SMAs, bilayer actuators, thermo-responsive LCEs, and hydrogels offer promise. Some two-way SMAs and bilayers can provide sharp, low-hysteresis responses in the environmental temperature range, while thermo-responsive hydrogels are capable of large but slow reversible deformations under sufficient humidity supply. LCEs are high-performance reversible thermal actuation materials, but their operation temperature range is beyond practical environmental applications. From a life-cycle sustainability perspective, biodegradable SMPs, biobased PCMs, and biodegradable/bioderived bilayers are the most environmentally compatible candidates, while SMAs, LCEs, and many synthetic hydrogels lack degradability. Future efforts should focus on tailoring transition temperatures to ambient ranges, minimizing hysteresis for cyclic operation, and advancing recyclable or biodegradable formulations.

*Hygroscopic materials* provide soft robots with the ability to deform under humidity variations, with performance governed by both intrinsic material properties and morphology design. Key intrinsic parameters such as CHE, SR, and adsorption kinetics determine actuation amplitude and response speed, but they vary widely across material classes. Wood and natural polysaccharides like cellulose, chitosan, and alginate offer a sustainable balance of swelling capability, mechanical robustness, and biodegradability, making them attractive for environmentally deployed devices, though their response rates are often modest. In contrast, nanomaterials such as MXenes and GO provide higher swelling or faster adsorption, with the added advantage of multistimuli responsiveness, but their long-term environmental compatibility remains uncertain. Morphology engineering through aerogels, hydrogels, or porous composites can significantly amplify water uptake and accelerate cycling, though typically at the cost of reduced mechanical strength and fatigue resistance. Future directions should focus on tailoring both material chemistry and structure to achieve fast, reversible responses under realistic humidity fluctuations, with the consideration of combining other environmental energy sources for improved water desorption.

*Photothermal materials* allow soft robots to directly harness sunlight for actuation by converting light into heat, where both energy conversion efficiency and mechanical resilience determine long-term performance. High-efficiency nanomaterials such as MXenes and CNTs offer broadband absorption and excellent thermal conductivity, enabling rapid heating and cooling cycles essential for reversible actuation. However, since these materials are typically integrated with thermo-responsive or hygroscopic components in composite architectures for actuation, compatibility and processability are critical for achieving reliable deformation without compromising mechanical robustness. While many synthetic nanomaterials provide unmatched efficiency, their environmental persistence raises concerns. In contrast, bioderived absorbers such as lignin or CINPs offer sustainable and biodegradable alternatives, though often with lower performance. Moving forward, optimizing composite design to balance conversion efficiency, mechanical durability, and ecological compatibility will be central to advancing sunlight-driven soft robotic systems.

*Osmotic materials* enable actuation in soft robots by exploiting osmotic potential difference (solute concentration gradients) to drive large and reversible swelling, with hydrogels (such as PAAm and PAMPS) and semipermeable polyelectrolytes standing out for their strong water uptake and mechanical compliance. In theory, such systems can operate reversibly by responding directly to environmental osmotic potential changes; however, practical limitations such as small environmental osmotic gradient, imperfect membrane selectivity, ion trapping within hydrogels, and hysteresis make fully reversible operation more difficult to achieve than heat, humidity, or sunlight-driven systems. From a life-cycle sustainability perspective, biodegradable hydrogels such as PVA and GelMA, together with benign osmolytes like organic acids, NaCl, or KCl, offer safe and eco-compatible options for deployment. Future progress will rely on refining hydrogel-osmolyte systems to improve durability, environmental compatibility, and combining other driving forces like electric fields to enhance efficiency and reversibility.

*pH-responsive materials* can serve as a foundation for environment-adaptive actuators by exploiting ionizable functional groups to trigger swelling or contraction under varying acidity or alkalinity. Their performance depends on combining strong hydrophilicity and pH sensitivity with sufficient mechanical robustness to endure repeated cycling without rupture. Widely studied synthetic polymers such as PAA and PDMAEMA offer tunable responsiveness and well-defined chemistry, but their limited biodegradability restricts sustainable applications. For outdoor and environmental use, materials must also maintain functionality under uncontrolled pH fluctuations, resist leaching, and withstand pollutants or temperature variations; in this regard, biodegradable polymers such as chitosan, alginic acid, hyaluronic acid, or CMC are promising alternatives. In biomedical contexts, biocompatibility and the safety of degradation products are paramount, with naturally derived and seminatural polymers offering tailored degradability and responses across diverse physiological pH ranges from the stomach to blood or tumor tissues.

*TENGs* offer a pathway to autonomous, untethered soft robotic systems through energy harvesting of ambient mechanical energy, in contrast to the previous categories, where sustainable energy sources are directly used to convert into mechanical actuation. In TENGs, material selection is central to their performance. Effective contact pairs typically combine negatively charging materials such as fluorinated polymers or silicones with positively charging counterparts like PA, PMMA, PU, metals, or biological surfaces, while surface micro/nanopatterning, chemical modification, and dielectric optimization can further boost charge density. Although in principle most materials exhibit triboelectric effects, reliable performance in practice is strongly influenced by environmental conditions, with humidity, surface degradation, and charge leakage often reducing output. These challenges are compounded when tribo-active layers must also fulfill additional roles, such as actuation or sensing, where multifunctionality rather than maximized triboelectric output drives material choice. Looking ahead, the development of robust, environmentally compatible tribo-active materials for energy harvesting, combined with parallel development of flexible/stretchable circuits, energy storage, and electrical actuation components, will be crucial for translating TENGs from laboratory devices into sustainable, field-deployable power sources for soft robotic sensors and actuators.

*Wind-dispersed fliers* draw inspiration from natural seeds and fruits, relying on lightweight polymers and architected geometries to achieve passive aerial dispersal. Materials such as PLGA and PI can be processed into ultrathin aerodynamic surfaces, while additive manufacturing of thermoplastics like PLA or CA enables lattice structures with controlled porosity that reduce weight without sacrificing aerodynamic surface area or stability. For large-scale environmental deployment where retrieval is impractical, biodegradable options such as CNCs, CA, PHA, or PCL are especially attractive to minimize ecological impact. Beyond passive flight, hybrid stimuli-responsive systems offer adaptive or externally controlled flight behaviors, but many current designs depend on not fully degradable materials like LCEs, LCNs, and MXenes. Moving forward, the integration of biodegradable responsive materials, such as bioderived photothermal absorbers, biodegradable SMPs, and bilayer composites, will be crucial for developing aerial robotic systems that combine efficient flight mechanics with environmental responsibility.

The main advantage of using sustainable energy sources in soft robotics is the reduced dependency on nonrenewable energy sources, contributing to a more environmentally sustainable approach and operating with minimal environmental impact. By utilizing natural and renewable energy, such as sunlight, wind, or temperature variations, soft robots reduce their dependence on conventional energy sources like electricity directly or indirectly produced from fossil fuel sources, making them suitable for deployment and distribution in ecosystems, remote areas, or environments with limited power supply. Many sustainable energy sources, such as sunlight, ambient temperature and humidity variations, or related to chemical potentials, are free, abundant, and hypothetically inexhaustible. This could allow soft robots to operate continuously for long periods without the need for recharging, enhancing efficiency and autonomy. This capability could be particularly appealing for applications such as autonomous environmental monitoring, search and rescue, and even space exploration. The analysis of state-of-the-art literature in this review highlights that, while significant discoveries have been made over the past 20–30 years in materials technology, soft actuators, and soft robots powered by sustainable and environmental energy sources, this field remains in its early stages from a technological and market perspective.

Viewed through the energetic sustainability framework adopted in this review, the current state of sustainable energy-powered soft robotics varies markedly across different energy forms. Systems driven by ubiquitous and easily accessible environmental stimuli, such as humidity, sunlight, and wind, have already demonstrated operation under fully sustainable conditions in some notable cases. In contrast, soft robots powered by temperature variations or gradients, osmotic and pH-driven processes, or triboelectric energy harvesting largely remain at laboratory or hybrid-driven stages, as their operation often depends on restricted environmental ranges, externally imposed gradients, or artificial intervention. These laboratory-level demonstrations nonetheless play a critical foundational role by establishing and validating material chemistries, actuation mechanisms, and structural design principles that inform future transitions toward environmentally realistic and autonomous operation.

Assessing TRLs is essential for evaluating the maturity of these technologies, ranging from early-stage research (TRL 1) to full commercialization (TRL 9).^571^ According with the analysis reported in this review the highest TRLs for each category of soft robots are: temperature-powered soft robots at maximum TRL 4 with the tubular rolling robot^180^ and the crawling robot driven by reversible snap-buckling;^116^ humidity-powered soft robots at TRL 7 with the autonomous self-burying seed carriers for aerial seeding being tested outdoor for 7 continuous days, with natural rainfalls;^199^ sunlight-powered soft robot at TRL 5 with an inchworm protype being demonstrated to crawl on a leaf under natural sunlight variations in a short time period;^352^ osmosis-powered soft actuators at maximum TRL 4 and soft robots at maximum TRL 3 for all the reported research tested under laboratory conditions; pH-driven soft actuators at maximum TRL 4 and soft robots at maximum TRL 3 for all the demonstrations tested under laboratory conditions; TENG-powered sensors at TRL 4 with a minority tested in relevant environments (TRL 5–6), but TENG-powered robots only at maximum TRL 3–4; wind-dispersed passive fliers at TRL 7 with the dandelion inspired battery-free wireless devices with sensors deployed and tested in outdoor environment.^545^

This analysis shows that even though significant scientific progress has been made, numerous obstacles remain before these innovations can be successfully implemented in real-world and commercial applications. The future of sustainable energy-powered soft robots will be influenced by progress in materials science, robotics, and engineering, which will be critical to bypass current limitations and increase their TRLs. A central constraint is that most passive, sustainable systems achieve repeated motion only by exploiting temporal variations in environmental stimuli, such as day-night temperature cycles, sunlight fluctuations, or changes in chemical potentials, thereby functioning primarily as adaptive devices rather than continuously powered robots. To achieve sustained motion under constant environmental input, more sophisticated design strategies are needed. Examples include leveraging spatial gradients of energy sources,^321^ integrating energy harvesting-storage systems to buffer intermittent supply, or adopting self-excited oscillators like LilBot that transform steady inputs into cyclic actuation.^355^ Furthermore, bistable or multistable structures and snapping mechanisms offer additional pathways for low-energy, electronics-free control by enabling rapid energy release and dynamic amplification.^116,277,572^ Together, these approaches highlight how creative material and structural design will be key to bridging the gap between adaptive environmental actuation and truly autonomous, continuous soft robotic operation.

Another limitation of soft robots powered by sustainable energy sources is their low force output, compared to rigid robots, which arises from their intrinsic mechanical properties and the constraints of energy conversion. In this context, the use of natural materials such as wood could represent a promising approach for humidity-driven soft robots offering a significantly higher modulus of approximately 10 GPa.^199^ Similarly, hydrogel actuators driven by sustainable energy sources generally suffer from weak actuation forces and slow response speeds due to their intrinsic softness and diffusion rate. In this context, the combination of multiple driving mechanisms from different stimuli and energy forms could be developed to enhance efficiency. For example, in the case of osmotic actuators, the integration of an electric field to combine turgor pressure and electroosmosis achieved significantly higher forces (up to 917 N) and faster actuation (3.5 N s^–1^).^388^

In most cases, the development of new smart multifunctional materials^24^ and (nano)­composites can improve the ability of soft robots to convert renewable energy sources more efficiently into usable energy. These materials should also exhibit durability, self-healing properties,^573^ and adaptability to diverse environmental conditions to ensure practical real-world implementation. Given that many soft robots powered by sustainable energy are designed for autonomous exploration of environments in large quantities, it would be necessary for them to be made also with biocompatible and (bio)­degradable materials, as it could be difficult to retrieve them. For this purpose, the use and integration of biodegradable or bioderived materials,^17^ such as cellulose-derived materials, PHA, or even edible materials,^574^ could further reduce the environmental impact of soft robots by enabling their natural degradation after use, thus reducing waste accumulation.

Ultimately, the synergy between material innovations, biodegradability, and sustainable energy could drive the transition from laboratory demonstrations to fully functional robots with high TRLs, playing a crucial role in the mitigation of climate change and supporting several of the UN’s Sustainable Development Goals (SDGs), both through a more responsible use of resources with an impact in SDG-7 (affordable and clean energy), SDG-12 (ensure sustainable consumption and production patterns) and SDG-13 (climate action), but also actively, by finding possible applications in environmental monitoring, reforestation, and remediation with an impact in SDG-14 (life below water) and SDG-15 (life on land).^575^

## †

s.mariani@fpscience.it; Fondazione Pisana Per La Scienza ONLUS, Via Ferruccio Giovannini, 13, 56017 San Giuliano Terme (Pisa), Italy

## §

kliton.cikalleshi@mpikg.mpg.de; Max Planck Institute of Colloids and Interfaces, Am Mühlenberg 1, 14476 Potsdam, Germany

## φ

luca.cecchini@roma1.infn.it; INFN Sezione di Roma P.le Aldo Moro, 2 c/o Dipartimento di Fisica “Sapienza” Università di Roma 00185 – Rome, Italy

## Abbreviations

°C: Degree Celsius
2D: Two-dimensional
2PP: Two-photon polymerization
3D: Three-dimensional
4D: Four-dimensional
A^3^: Aspartic acid *N*-acrylamide
a-Si: Amorphous silicon
*A*_*f*_: Austenite finish temperature
AAc: Acrylic acid
AAm: Acrylamide
NaAc: Sodium acrylate
ABA: Triblock architecture
AgNPs: Silver nanoparticles
AL: Active layer
AVFL: Artificial Venus flytrap
BG1: Binary gel 1
BG2: Binary gel 2
BL: Body length
CA: Cellulose acetate
CAL: Computed axial lithography
C_d_: Change degree in surface area
CDs: Carbon dots
CDs-SA: Carbon dots chemically cross-linked with sodium alginate
CHE: Coefficient of hygroscopic expansion
CICC: CINPs/CNF composite
CIGS: Copper indium gallium diselenide
CINPs: Cuttlefish ink nanoparticles
CLP: Candle soot-LCE/PDMS
CMC: Carboxymethyl cellulose
CNC: Cellulose nanocrystal
CNF: Cellulose nanofiber
CNT: Carbon nanotube
CST: Critical solution temperature
CTE: Coefficients of thermal expansion
CVD: Chemical vapor deposition
DEA: Dielectric elastomer actuator
DIW: Direct ink writing
DLP: Digital light processing
DMAc: Dimethylacetamide
DMAEMA: 2-(*N,N*-dimethylamino)­ethyl methacrylate
DMAEMA-Q: Acrylamide/quaternized dimethylaminoethyl methacrylate
DMAPS: 3-dimethyl (methacryloyloxyethyl) ammonium propanesulfonate
DMF: *N,N*-dimethylformamide
DMSO: Dimethyl sulfoxide
DSSCs: Dye-sensitized solar cells
EDL: Electric double layer
EOF: Electroosmotic flow
FA: Formamide
FEP: Fluorinated ethylene propylene
FsLDW: Femtosecond laser direct writing
G/GO: Graphene/graphene oxide
GelMA: Gelatin methacrylate
GelWC: 3:1 DMAPS:MAA
GO: Graphene oxide
GPS: Global positioning system
HEMA: Hydrogels hydroxyethyl methacrylate
HFA: Hexafluoroacetone
HFIP: Hexafluoro-iso-propanol
IL: Inactive layer
IoT: Internet of Things
IPMCs: Ionic polymer–metal composites
IPTS: Isocyanatopropyltriethoxysilane
IR: Infrared
LCE: Liquid crystalline elastomer
LCN: Liquid crystal networks
LCP: Liquid crystal polymer
LCST: Lower critical solution temperature
LD_50_: Lethal dose 50
LDPE: Low-density polyethylene
LED: Light-emitting diode
LEV: Leading-edge vortex
LSPR: Localized surface plasmon resonance
MAA: Methacrylic acid
MA-BSA: Methacrylated bovine serum albumin
MA-FIID: Methacrylated fluorescein isothiocyanate derivative
MBA: Methylenebisacrylamide
MEMS: Microelectromechanical systems
*M*_f_: Martensite finish temperature
MFA: *N*-methylformamide
MMA-co-PEGDMA: Methyl methacrylate-*co*-poly­(ethylene glycol) dimethacrylate
MPO: Myeloperoxidase
Mt: Morillonite
MXSA: MXene/sodium alginate composite
MWCNT: Multiwalled carbon nanotube
MXCC: MXene-cellulose composites
NIR: Near-infrared
NiTi: Nickel-titanium
NMMO: *N*-methylmorpholine-*N*-oxide
nMOS: Negative metal-oxide-semiconductor
NMP: *N*-methyl-2-pyrrolidone
NPs: Nanoparticles
NR: Not reported
OWSME: One-way shape-memory effect
P­(AA-*co*-BMA): Poly­(acrylamide-*co*-butyl methacrylate-co-acrylic acid)
P­(AA-DMAEMA): Poly­(acrylic acid-*co*-2-(*N,N*-dimethylamino)­ethyl methacrylate)
p­(HEMA-*co*-AA): Poly­(2-hydroxyethyl methacrylate-co-acrylic acid)
P­(VDF-TrFE): Poly­(vinylidenefluoride-*co*-trifluoroethylene)
PA: Polyamide
PAA: Poly­(acrylic acid)
PAA-*co*-4VP: Poly­(acrylic acid)-*co*-4-vinylpyridine
PAA-*co*-DMAEMA: Poly­(acrylic acid)-*co*-poly­(2-(*N,N*-dimethylamino)­ethyl methacrylate)
PAAm: Polyacrylamide
PAMAM: Polyamidoamine
PAMPS: Poly­(2-acryloamido-2-methyl propane) sulfonic acid
PC: Polycarbonate
PCB: Printed circuit board
PCE: Photothermal conversion efficiency
PCL: Poly­(ε-caprolactone)
PCM: Phase change material
PDADMAC: Poly­(dimethyldiallylammonium chloride)
PDMAEMA: Poly­(2-(*N,N*-dimethylamino)­ethyl methacrylate)
PDMS: Polydimethylsiloxane
PE: Polyethylene
PEG: Poly­(ethylene glycol)
PEGDA: Poly­(ethylene glycol)­diacrylate
PEI: Polyethylenimine
PEO: Polyethylene oxide
PET: Poly­(ethylene terephthalate)
PFSA: Perfluorosulfonic acid ionomer
PG: Primary gel
PGA: Poly glycolic acid
PHA: Polyhydroxyalkanoates
PHBV: Poly­(3-hydroxybutyrate-*co*-3-hydroxyvalerate)
PI: Polyimide
PLA: Poly­(lactic acid)
PLGA: Poly­(lactic-*co*-glycolic acid)
PMMA: Polymethyl methacrylate
PMMA/SPEG: Poly­(methyl methacrylate)/star poly­(ethylene glycol)
PMMA-*co*-DMAEMA: Poly­(methyl methacrylate)-*co*-poly­(2-(*N,N*-dimethylamino)­ethyl methacrylate)
PMMA-*g*-PEG: Poly­(methyl methacrylate)-*graft*-poly­(ethylene glycol)
PNAGA: Poly­(*N*-acryloyl glycinamide)
PNIPAM: Poly­(*N*-isopropylacrylamide)
PPO: Poly­(propylene oxide)
PPy: Polypyrrole
PSCs: Perovskite solar cells
PSF: Polysulfone
PSPA: Poly­(3-sulfopropylacrylate) potassium salt
PEDOT:PSS: Poly­(3,4-ethylenedioxythiophene: polystyrene sulfonate
PTFE: Polytetrafluoroethylene
PTMG: Poly­(tetramethylene glycol)
PU: Polyurethane
PV: Photovoltaic
PVA: Polyvinyl alcohol
PVDF-HFP: Poly­(vinylidene fluoride)-hexafluoropropylene
PVDF-HFP-TFE: Poly­(vinylidene fluoride-hexafluoropropylene-tetrafluoroethylene)
PVP: Polyvinylpyrrolidone
RH: Relative humidity
SA: Sodium alginate
SBC: Styrenic block copolymer
SBS: Poly­(styrene-*b*-butadiene-*b*-styrene)
SEBS: Polystyrene-*b*-poly­(ethylene-*co*-butylene)-*b*-polystyrene
SEM: Scanning electron microscopy
SF: Silk fibroin
SLA: Stereolithography
SMA: Shape-memory alloy
SMP: Shape-memory polymer
SR: Swelling ratio
SVR: Separated vortex ring
*T*: Temperature
TBA: Tribromoacetic acid
TBAH: Tetra­(n-butyl)­ammonium
TEGs: Triboelectric energy generators
TENGs: Triboelectric nanogenerators
*T*_g_: Glass transition temperature
*T*_m_: Melting temperature
*T*_NI_: Nematic–isotropic transition temperature
TRL: Technology readiness level
*T*_trans_: Transition temperature
TWSME: Two-way shape-memory effect
UAVs: Unmanned aerial vehicles
UCST: Upper critical solution temperature
UV: Ultraviolet
VOCs: Volatile organic compounds
